# Draft genome sequence of *Solanum aethiopicum* provides insights into disease resistance, drought tolerance, and the evolution of the genome

**DOI:** 10.1093/gigascience/giz115

**Published:** 2019-10-01

**Authors:** Bo Song, Yue Song, Yuan Fu, Elizabeth Balyejusa Kizito, Sandra Ndagire Kamenya, Pamela Nahamya Kabod, Huan Liu, Samuel Muthemba, Robert Kariba, Joyce Njuguna, Solomon Maina, Francesca Stomeo, Appolinaire Djikeng, Prasad S Hendre, Xiaoli Chen, Wenbin Chen, Xiuli Li, Wenjing Sun, Sibo Wang, Shifeng Cheng, Alice Muchugi, Ramni Jamnadass, Howard-Yana Shapiro, Allen Van Deynze, Huanming Yang, Jian Wang, Xun Xu, Damaris Achieng Odeny, Xin Liu

**Affiliations:** 1 BGI-Shenzhen, Beishan Industrial Zone, Yantian District, Shenzhen 518083, China; 2 China National GeneBank, BGI-Shenzhen, Jinsha Road, Shenzhen 518120, China; 3 State Key Laboratory of Agricultural Genomics, BGI-Shenzhen, Shenzhen 518083, China; 4 BGI-Qingdao, BGI-Shenzhen, Qingdao 266555, China; 5 Uganda Christian University, Bishop Tucker Road, Box 4, Mukono, Uganda; 6 Biosciences Eastern and Central Africa (BecA) – International Livestock Research Institute (ILRI) Hub, P.O. Box 30709, Nairobi 00100, Kenya; 7 African Orphan Crops Consortium, World Agroforestry Centre (ICRAF), United Nations Avenue, Nairobi 00100, Kenya; 10 University of California, 1 Shields Ave, Davis, CA, USA; 11 Mars, Incorporated, 6885 Elm Street, McLean, VA 22101, USA; 12 International Crops Research Institute for the Semi-Arid Tropics (ICRISAT) – Eastern and Southern Africa, P.O. Box 39063, Nairobi 00623, Kenya

**Keywords:** *Solanum aethiopicum*, African eggplant, *Solanum anguivi*, LTR-Rs, biotic stress, drought tolerance

## Abstract

**Background:**

The African eggplant (*Solanum aethiopicum*) is a nutritious traditional vegetable used in many African countries, including Uganda and Nigeria. It is thought to have been domesticated in Africa from its wild relative, *Solanum anguivi. S. aethiopicum* has been routinely used as a source of disease resistance genes for several Solanaceae crops, including *Solanum melongena*. A lack of genomic resources has meant that breeding of *S. aethiopicum* has lagged behind other vegetable crops.

**Results:**

We assembled a 1.02-Gb draft genome of *S. aethiopicum*, which contained predominantly repetitive sequences (78.9%). We annotated 37,681 gene models, including 34,906 protein-coding genes. Expansion of disease resistance genes was observed via 2 rounds of amplification of long terminal repeat retrotransposons, which may have occurred ∼1.25 and 3.5 million years ago, respectively. By resequencing 65 *S. aethiopicum* and *S. anguivi* genotypes, 18,614,838 single-nucleotide polymorphisms were identified, of which 34,171 were located within disease resistance genes. Analysis of domestication and demographic history revealed active selection for genes involved in drought tolerance in both “Gilo” and “Shum” groups. A pan-genome of *S. aethiopicum* was assembled, containing 51,351 protein-coding genes; 7,069 of these genes were missing from the reference genome.

**Conclusions:**

The genome sequence of *S. aethiopicum* enhances our understanding of its biotic and abiotic resistance. The single-nucleotide polymorphisms identified are immediately available for use by breeders. The information provided here will accelerate selection and breeding of the African eggplant, as well as other crops within the Solanaceae family.

## Background

The African eggplant, *Solanum aethiopicum* (NCBI:txid205524), is an indigenous non-tuberiferous Solanaceae crop that is mainly grown in tropical Africa [1], especially in Central and West Africa. *S. aethiopicum* is hypervariable [2, 3] and is generally classified into 4 groups: Gilo, Shum, Kumba, and Aculeatum. Gilo is the most important group and has edible fruits, while Shum has small and bitter fruits. Kumba is used as a leafy vegetable, while Aculeatum is used as an ornamental [3, 4] or as rootstock because of its excellent disease resistance [5]. The African eggplant is reported to have anti-inflammatory activity [6], and its roots and fruits have been used to treat colic, high blood pressure, and uterine complications in Africa [6].

Although *S. aethiopicum* is one of the most important cultivated eggplants in Africa [7, 8], it remains an “orphan crop” because research and breeding investments are lagging behind other Solanaceae relatives, such as *Solanum lycopersicum* (tomato), *Solanum tuberosum* (potato), and *Solanum melongena* (edible eggplant). Consequently, there have been few robust genomic resources, such as a well-annotated reference genome. Genomics-assisted breeding is an effective approach that would facilitate the breeding of orphan crops such as the African eggplant. Previous attempts to develop molecular markers for *S. aethiopicum*, using the *S. melongena* genome as a reference, have been unsuccessful because of compromised accuracy [9]. An alternative approach that uses genome editing has been successfully deployed in other Solanaceae crops, including *Physalis pruinose* [11, 12], but cannot be implemented in *S. aethiopicum* because of its lack of well-annotated reference genome and gene sequences.

The African eggplant serves as a gene reservoir for other economically important crops within the Solanaceae family. Thanks to its cross-compatibility with *S. melongena* [4, 10] and its outstanding resistance to various pathogens, including *Fusarium, Ralstonia*, and *Verticillium* [5, 11–13], *S. aethiopicum* has been used to develop rootstocks [13] or improve the disease resistance of *S. melongena* [14]. Because the genomic basis of resistance in *S. aethiopicum* is poorly understood, it can be time-consuming to use it as a donor in such interspecific crosses. Mapping resistance genes and then developing markers associated with these genes might resolve this challenge. The development and expansion of resistance genes is usually accompanied by the amplification of long terminal repeat retrotransposons (LTR-Rs). A typical example is shown in the Solanaceous hot pepper (*Capsicum annuum*), in which a burst of LTR-Rs substantially mediated the retrotransposition of nucleotide-binding, leucine-rich repeat-related (NLR) genes, leading to the expansion of resistance genes [15]. LTR-Rs are abundant in plant genomes, including Solanaceae crops such as *Nicotiana sylvestris* (∼38.16%) [16], pepper (>70.0%) [17], potato (62.2%) [18], tomato (50.3%) [19], and petunia (>60%) [20]. The role of LTR-Rs in the *S. aethiopicum* genome remains unknown, and whether the resistance seen in *S. aethiopicum* is a result of LTR-R amplification remains to be investigated. The generation of a reference genome for *S. aethiopicum*, as well as for other orphan crops, is urgently needed to advance their research and breeding.

Here, we report a draft whole-genome assembly and annotation for *S. aethiopicum*. We found 2 amplifications of LTR-Rs that occurred ∼1.25 and 3.5 million years ago (MYA), resulting in the expansion of resistance genes. We also resequenced 2 *S. aethiopicum* groups, “Gilo” and “Shum," and *S. anguivi* at a high depth (∼60×) and identified 18,614,838 single-nucleotide polymorphisms (SNPs), 34,171 of which are located within resistance genes. Subsequently, we generated a pan-genome of *S. aethopicum*. The genomic data provided in this study will greatly advance research and breeding activities of the African eggplant.

## Data Description

We sequenced the genome of *S. aethiopicum* using a whole-genome shotgun approach. A total of 242.61 Gb raw reads were generated by sequencing the libraries with insert sizes of 250 and 500 bp, and mate-pair libraries with sizes ranging between 2,000 and 20,000 bp, on an Illumina Hiseq 2000 platform. The filtered reads used for downstream analysis are shown in Supplementary Table 1. *k*-mer (*k* = 17) analysis [21] revealed the *S. aethiopicum* genome to be diploid and homozygous, with an estimated genome size of 1.17 Gb (Supplementary Fig. 1). “Clean reads” amounting to 127.83 Gb (∼109×) were used to assemble the genome using Platanus [22] (see Methods). A final assembly of 1.02 Gb in size was obtained, containing 162,187 scaffolds with N50 contig and scaffold values of 25.2 and 516.15 kb (Table 1 and Supplementary Table 2), respectively. Our results reveal that the *S. aethiopicum* genome is larger than that of other *Solanum* genomes, including tomato (0.76 Gb) and potato (0.73 Gb) [18, 19], but it has a comparable guanine-cytosine (GC) ratio (33.12%) (Supplementary Table 3).

**Table 1:** Statistical data for the *Solanum aethiopicum* genome and gene annotation

Parameter	Value
Scaffolds	
Number	162,187
Total length	1.02 Gb
N50	516.1 kb
Longest	2.94 Mb
Contigs	
Number	231,821
Total length	936 Mb
N50	25.2 kb
Longest	366.2 kb
GC content	33.13%
Number of genes	34,906
Average/total coding sequence length	1104.3 bp/38.5 Mb
Average exon/intron length	265.8 bp/613.1 bp
Total length of transposable elements	805.7 Mb (78.23%)

Repetitive elements, predominantly transposable elements (TEs) (Supplementary Table 4), occupied 811 Mb (78.9%) of the sequenced genome. Most annotated TEs were retrotransposon elements, including long terminal repeats (LTRs), short interspersed nuclear elements, and long interspersed nuclear elements. Together these retrotransposons made up 75.42% of the assembly. DNA transposons accounting for 2.87% of the genome were also annotated (Supplementary Table 4).

Protein-coding gene models were predicted by a combination of homologous search and *ab initio* prediction. The resulting models were pooled to generate a final set of 34,906 protein-coding genes. Predicted gene models were, on average, 3,038 bp in length, with a mean of 3.15 introns. The mean length of coding sequences, exons, and introns was 1,104, 265, and 613 bp, respectively (Table 1, Supplementary Table 5, Supplementary Fig. 2). As expected, these gene features were similar to those of other released genomes, including *Arabidopsis thaliana* [23] and other Solanaceae crops including *S. lycopersicum, S. tuberosum, C. annuum*, and *N. sylvestris* [16, 18, 19, 24] (Supplementary Table 5). We further assessed the annotation completeness of this assembly by searching for 1,440 core embryophyta genes (CEGs) with BUSCO, version 3.0 [25]. We found 80.4% CEGs in this assembly, with 77.8% being single copies and 2.6% being duplicates (Supplementary Table 6). We also annotated the non-coding genes by homologous search, leading to the identification of 128 microRNA, 960 transfer RNA (tRNA), 1,185 ribosomal RNA (rRNA), and 503 small nuclear RNA (snRNA) genes (Supplementary Table 7).

We annotated 31,863 (91.28%) proteins for their homologous function in several databases. Homologs of 31,099 (89.09%), 26,319 (75.4%), and 20,932 (59.97%) proteins were found in TrEMBL, InterPro, and SwissProt databases, respectively (Supplementary Table 8). The remaining 3,043 (8.72%) genes encoded putative proteins with unknown functions.

## Analyses

### Genome evolution and phylogenetic analysis

**Figure 1:**
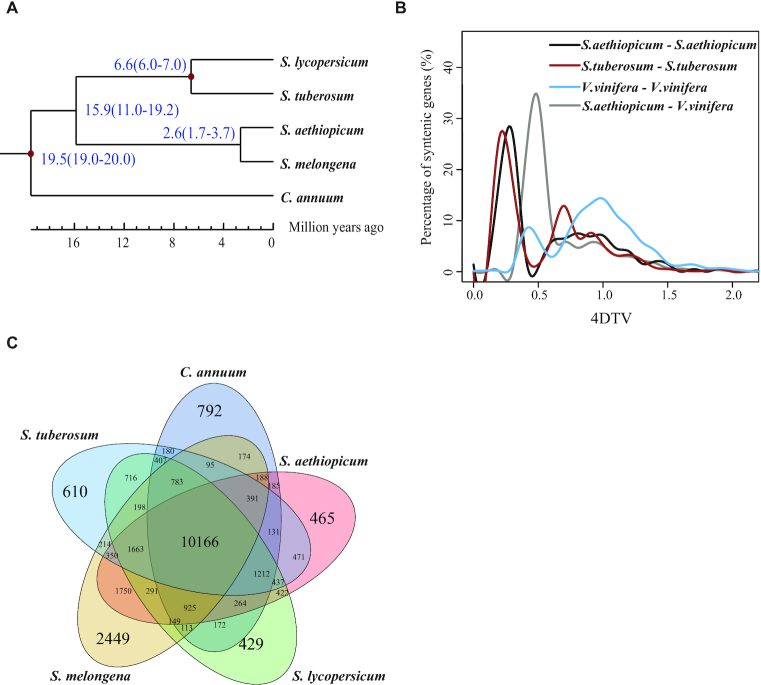
Comparative analysis of the *Solanum aethiopicum* genome. (**A**) Phylogenetic analysis of *Solanum melongena, S. lycopersicum, S. tuberosum, S. aethiopicum*, and *Capsicum annuum* using single-copy gene families. The species differentiation time between *S. aethiopicum* and *S. melongena* was 2.6 million years. (**B**) Distribution of 4DTv distance, which showed 2 peaks ∼0.25 and 1 (black line), representing 2 whole-genome duplication events. (**C**) Venn diagram showing overlaps of gene families between *S. melongena, S. lycopersicum, S. tuberosum, S. aethiopicum*, and *C. annuum*. A total of 465 gene families were unique to *S. aethiopicum* and 10,166 were common to the genomes of the 5 species. *V. vinifera: Vitis vinifera*.

By comparing with 4 other sequenced Solanaceae genomes (*S. melongena, S. lycopersicum, S. tuberosum*, and *C. annuum*), 25,751 of the *S. aethiopicum* genes were clustered into 19,310 families using OrthoMCL (version 2.0) [26], with an average of 1.33 genes each. Single-copy genes shared by these 5 genomes were concatenated as a supergene representing each genome and were used to build a phylogenetic tree (Fig. 1A). The split time between *S. aethiopicum* and *S. melongena* was estimated to be ∼2.6 MYA. McScanX [27] identified 182 syntenic blocks. We detected evidence of whole-genome duplication (WGD) events in this genome by calculating the pairwise synonymous mutation rates and the rate of 4-fold degenerative third-codon transversion (4DTV) of 1,686 paralogous genes in these blocks. The 4DTV distribution plot displayed 2 peaks, at ∼0.25 and 1, indicating 2 WGDs (Fig. 1B). The first 1 (peak at 1) represents the ancient WGD event shared by asterids and rosids [28], while the second WGD event is shared by Solanaceae plants. This suggests that its occurrence predates the split of Solanaceae.

### Evolution of gene families

OrthoMCL [26] clustering of genes from *S. aethiopicum, S. melongena, S. lycopersicum, S. tuberosum*, and *C. annuum* identified 25,751 gene families. Among these, 465 gene families were unique to *S. aethiopicum* and 10,166 were common (Supplementary Table 9, Fig. 1C). As expected, the number of shared gene families decreased as a function of evolutionary distance between *S. aethiopicum* and the selected species (Supplementary Table 10). For example, *S. aethiopicum* shared 15,723 gene families with *S. melongena*, compared with only 13,461 genes shared with *C. annuum*. To further investigate the evolution of gene families, we identified expanded and contracted gene families. Compared with *S. melongena*, 437 gene families were expanded; most expanded gene families were found to be involved in biological processes related to drought or salinity tolerance or disease resistance, including defense response (GO:0006952), response to oxidative stress ( GO:0006979), glutamate biosynthetic processes ( GO:0006537), and response to metal ions ( GO:0010038) (Supplementary Table 11). No gene families were contracted when comparing with *S. melongena*.

### Amplification of LTR-Rs

LTR-Rs made up ∼70% of the genome and accounted for 89.31% of the total TEs in *S. aethiopicum* (Supplementary Table 4). Consistent with previous studies of LTR-Rs, most LTR-Rs were classified as being in *Ty3/Gypsy* (82.36% of total LTR-Rs) and *Ty1/Copia* (14.90% of total LTR-Rs) subfamilies. The proportion of *Ty3/Gypsy* in *S. aethiopicum* is comparable to that reported in the hot pepper genome (87.7% of *Ty3/Gypsy*) [24]. To investigate the roles of LTR-Rs in the evolution of *S. aethiopicum*, we detected 36,599 full-length LTR-Rs using LTRharvest [29] with the parameters “-maxlenltr 2000, -similar 75” and LTRdigest software [30]. We further analyzed their evolution, activity, and potential biological functions.

**Figure 2:**
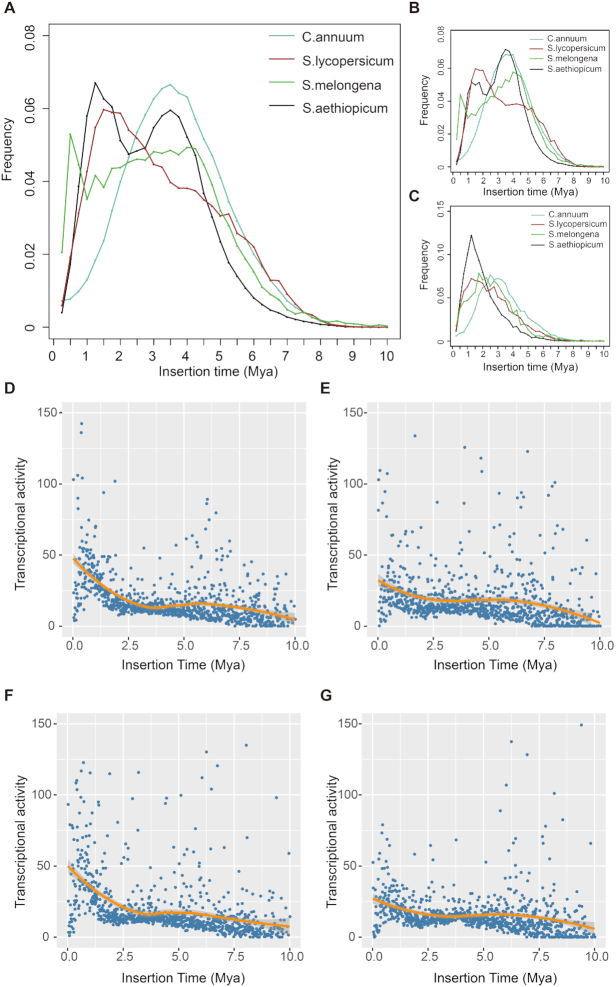
Long terminal repeat retrotransposon (LTR-R) insertion time distribution and the expression level of LTR-Rs in different tissues. Insertion time distribution of total LTR-Rs (**A**), *Ty3/Gypsy* LTR-Rs (**B**), and *Ty1/Copia* LTR-Rs (**C**) of *Capsicum annuum, Solanum melongena, S. lycopersicum*, and *S. aethiopicum*. The x- and y-axes, respectively, indicate the insertion time and the frequency of inserted LTR-Rs. Expression levels of LTR-Rs in flower (**D**), fruit (**E**), leaf (**F**), and root (**G**) tissues.

The age of each LTR-R was inferred by comparing the divergence between the 5′ and 3′ LTR-R, using a substitution rate of 1.3e−8 year^−1^ site^−1^ [31]. Two amplifications of LTR-Rs were found in *S. aethiopicum*, while only 1 was detected in tomato and hot pepper (Fig. 2A). The early amplification occurred at ∼3.5 MYA, coincident with the LTR-R burst found in *C. annuum* [15] (Fig. 2A). The second amplification was at 1.25 MYA, coinciding with the LTR-R burst in the tomato genome [19] (Fig. 2A). Although the time of LTR-Rs amplification is vertically coincident between different species, they occurred separately in each genome since the ancestor of *S. aethiopicum* diverged from that of hot pepper and tomato ∼20 and 4 MYA, respectively (Fig. 1A). These results imply that environmental stimulators shared between these species during their evolution could have triggered the amplifications observed. We also estimated the amplification time of *Ty3/Gypsy* and *Ty1/Copia* LTR-Rs and found 2 peaks at ∼1.25 and 3.5 MYA for Gypsy LTR-Rs (Fig. 2B) but only 1 peak (∼1.25 MYA) for *Ty1/Copia* LTR-Rs (Fig. 2C). Compared with the amplification time of *Ty3/Gypsy* and *Ty1/Copia* LTR-Rs in different species, we observed that the insertion time of *Ty1/Copia* LTR-RTs in *S. aethiopicum* and tomato were earlier than that of *S. melongena* and hot pepper. On the contrary, the insertion time of *Ty3/Gypsy* LTR-RTs (∼3.5 MYA) in *S. aethiopicum* was consistent with the insertion time of hot pepper (Fig. 2B and C).

To investigate the activities of these LTR-Rs, we measured their expression levels by using RNA-sequencing (RNA-seq) data from different tissues (see Methods). Younger LTR-Rs were expressed at higher levels than those of older LTR-Rs. We detected 2 peaks of LTR-R activity, at positions corresponding to the 2 rounds of LTR-R insertions (Fig. 2D–G). The slight shift of the former peaks indicates that the activities degenerated more slowly than the LTR-R sequences (Fig. 2D–G). The LTR-R activities varied across these tissues. The degeneration of LTR-R activities was slower in fruits and roots than those in flowers and leaves (Fig.   2D). This pattern was also confirmed by the varied activity of each LTR-R across these tissues (Supplementary Fig. 3A), implying that these LTR-Rs have different roles in development.

### Increased resistance is facilitated by LTR-R amplification

**Figure 3:**
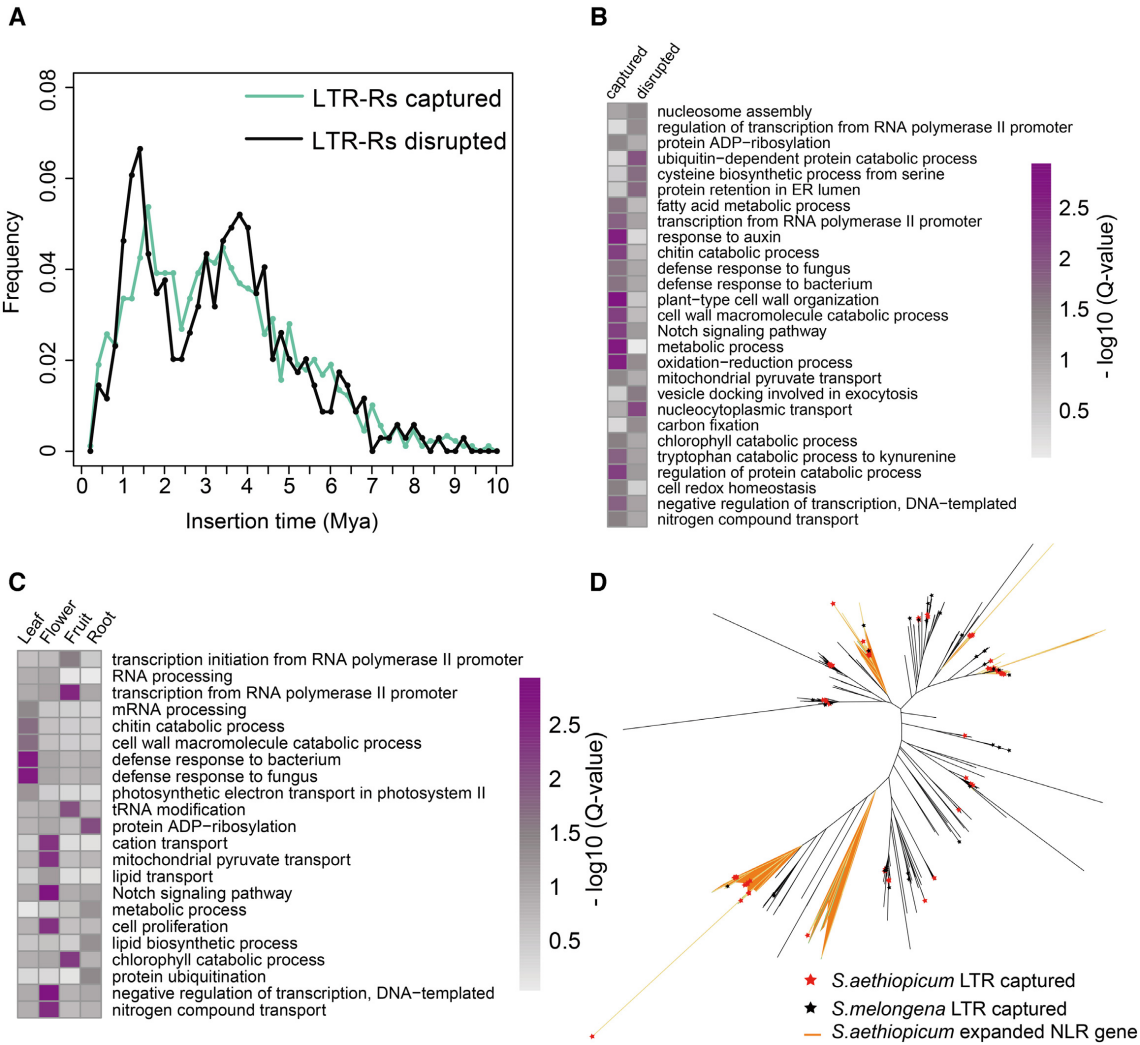
LTR-R captured and disrupted genes. (**A**) The distribution of ages of LTR-R captured and disrupted genes. (**B**) GO enrichment analysis between the LTR-R captured and disrupted gene set. (**C**) GO terms enriched in LTR-R captured genes that are specifically and highly expressed in various tissues, including leaf, flower, root, and fruit. (**D**) Phylogenetic tree of the nucleotide-binding, leucine-rich repeat-related (NLR) gene in *Solanum aethiopicum* and *S. melongena*. ADP: adenosine diphosphate; ER: endoplasmic reticulum.

We identified 1,156 LTR-R captured genes and 491 LTR-R disrupted genes. The insertion time of LTR-R captured and LTR-R disrupted genes both ranged between 1.5 and 3.5 MYA (Fig. 3A), showing a pattern similar to the insertions of whole LTR-Rs (Fig. 2A). These results suggest that LTR-R–mediated gene disruption and capture occurred simultaneously. We further classified the LTR-R captured genes into Gene Ontology (GO) categories and performed GO enrichment analysis. GO terms related to disease resistance including “defense response to fungus (GO:0006952)," “chitin catabolic process (GO:0006032)," “chitinase activity (GO:0004568)," “chitin binding (GO:0008061)," “cell wall macromolecule catabolic process (GO:0016998),” and “defense response to bacterium (GO:0042742)” were overrepresented in the LTR-R captured genes (Fig.   3B, Supplementary Table 12), suggesting that they may be involved in enhancing disease resistance.

We also analyzed the expression of genes captured by LTR-Rs. It was intriguing to find that most of these genes were active in only 1 tissue (Supplementary Fig. 3B). Among these genes, 159 (13.75%), 105 (9.08%), 106 (9.16%), and 129 (11.15%) were specifically and highly expressed in root, leaf, flower, and fruit, respectively. The genes captured by LTR-Rs that were specifically active in leaf tissues were significantly enriched in functions relating to disease resistance (Supplementary Table 13). The biological processes and molecular activities related to disease resistance mentioned above were overrepresented in these genes (Fig.   3C). The high expression level of resistance genes in leaves would arm the plant with stronger resistance to pathogens. On the contrary, these GO terms were not enriched in the genes that were specifically and highly expressed in leaves. Instead, as expected, “photosynthesis” and “photosystem I” were significantly overrepresented (Supplementary Table 14). The discrepancy between these 2 gene sets highlights the contribution to resistance of LTR-R captured genes.

Proteins containing nucleotide-binding, leucine-rich repeat domains (NB-LRRs) are major components that are responsible for defense against various phytopathogens [32]. The NB-LRR family is highly expanded in plants, with numbers ranging from <100 to >1,000 [33, 34]. As NB-LRR genes are often co-localized with LTR-Rs [35], we inspected their genomic locations in the *S. aethiopicum* genome. Because proteins containing the nucleotide-binding (NB) site can also confer disease resistance, we searched for all the NB-containing genes in the genome. As a result, we identified 447 NB-containing genes in the genome, among which 62 (13.9%) NB-containing genes co-localized with LTR-Rs were identified as LTR-R captured genes. The phylogenetic tree shows a substantial expansion of NB-containing genes after the amplification of LTRs in *S. aethiopicum* (Fig. 3D). A similar expansion was also observed in *S. melongena*. However, the number was remarkably fewer than in *S. aethiopicum*, probably because of the limited number of LTR-Rs in the *S. melongena* genome (Supplementary Table 15).

### Polymorphisms in different *S. aethiopicum* groups

We resequenced 60 *S. aethiopicum* genotypes in 2 major groups, Gilo and Shum, and 5 accessions of *S. anguivi*, the progenitor of *S. aethiopicum* [36]. We generated ∼60 Gb raw data (60×) (Supplementary Table 20) and identified 18,614,838 SNPs and 1,999,241 indels, with an average of 3,530,488 SNPs for each accession (Supplementary Table 16). On average, there were 18,090 SNPs and 1,943 indels per megabase. Among them, 424,509 (2.06%), 815,217 (3.95%), and 19,374,353 (93.99%) were located in exons, introns, and intergenic regions, respectively (Table 2). There were 267,710 SNPs that resulted in amino acid sequence changes by introducing new start codons, premature stop codons, or nonsynonymous substitutions (Table 2). We also identified 1,255,302 structural variations (SVs). Of the detected indels, 177,711 (8.89%) were located in genic regions, among which 2,977 caused frameshift changes and, therefore, resulted in amino acid sequence changes that may have led to gene malfunctions. Furthermore, 106,377 SVs were identified in genic regions, including 53,736 (50.51%) deletions, 34,368 (32.31%) insertions, and 8,872 (8.34%) duplications.

**Table 2:** Statistical data for single-nucleotide polymorphisms and indels of 65 accessions

Type	Class	No. (%)
SNPs	Exon	392,160 (2.11)
	Intron	669,855 (3.60)
	Intergenic	17,552,823 (94.29)
	Synonymous	126,172 (0.68)
	Nonsynonymous	267,710 (1.44)
	**Total**	**18,614,838**
Indels	Exon	32,349 (1.62)
	Intron	145,362 (7.27)
	Intergenic	1,821,530 (91.11)
	Frame shift	2,977 (0.15)
	**Total**	**1,999,241**

**Figure 4:**
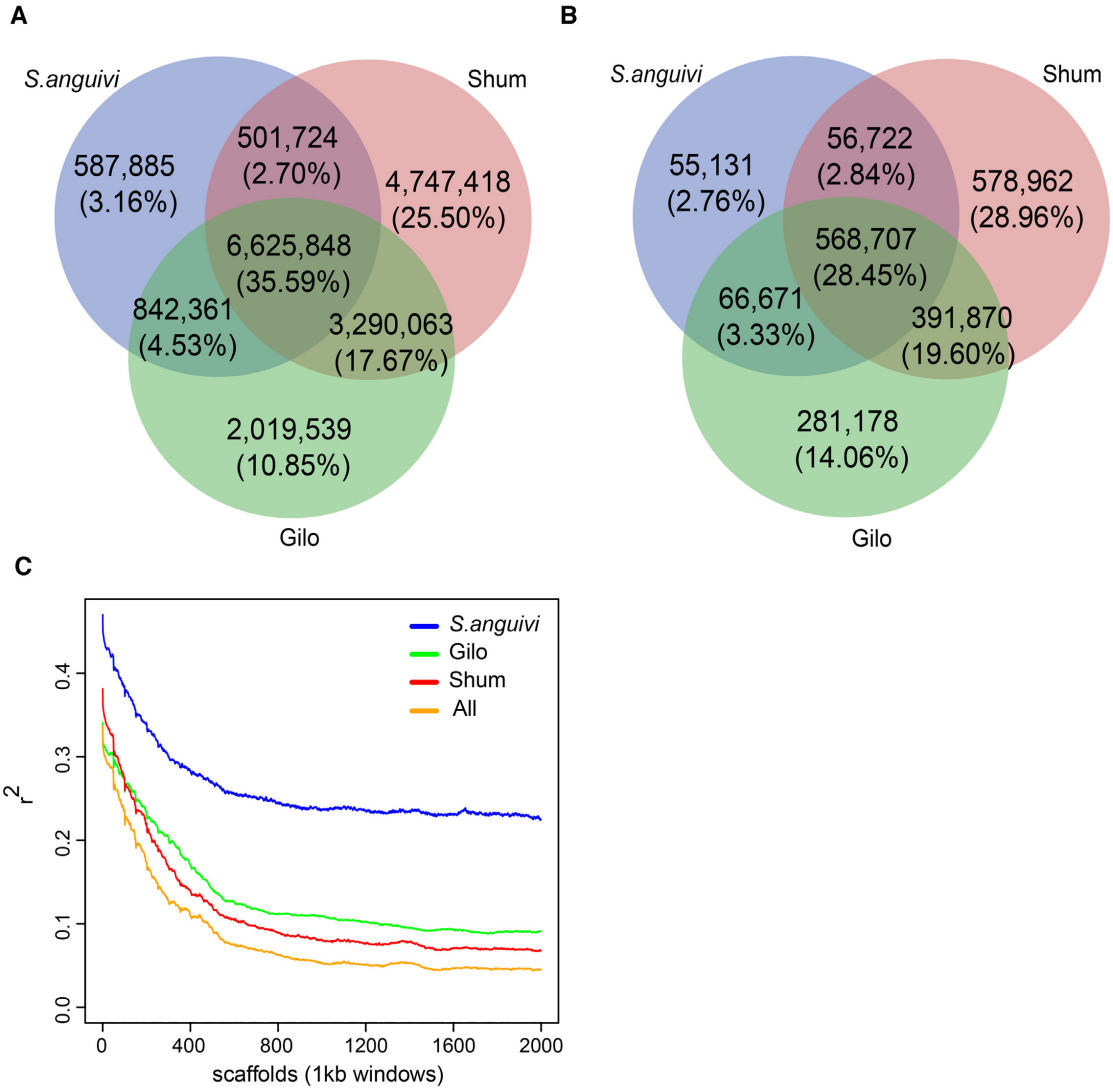
Single-nucleotide polymorphisms (SNPs), indel, and linkage disequilibrium (LD) decay for "Gilo," "Shum," and *"S. anguivi*" groups. (**A**) SNPs numbering 2,019,539 (10.85%%), 4,747,418 (25.50%), and 587,885 (3.16%) were unique to Gilo, Shum, and *S. anguivi*, respectively. Most (93.13%) of SNPs in *S. anguivi* were shared with either Gilo or Shum. (**B**) Indels amounting to 14.06%, 28.96%, and 2.76% were unique to Gilo, Shum, and *S. anguivi*, respectively, and, like the SNP statistics in these groups, 92.62% of indels in *S. anguivi* were shared with either Gilo or Shum. (**C**) LD estimation revealed that *r*^2^ reaches the half maximum value at ∼150 kb.

On counting the SNPs and indels in each group, we found 12,777,811, 15,165,053 and 8,557,818 SNPs in “Gilo," “Shum,” and “*S. anguivi*," respectively, accounting for 68.64%, 81.47%, and 45.97% of the total SNPs, respectively. There were 2,019,539 (10.85%), 4,747,418 (25.50%), and 587,885 (3.16%) SNPs unique to Gilo, Shum, and *S. anguivi*, respectively (Fig. 4A). Most (93.13%) SNPs in *S. anguivi* were shared with either Gilo or Shum (Fig. 4A), which is in line with the fact that *S. anguivi* is the ancestor [36]. Similarly, 92.62% of the indels identified in *S. anguivi* were also shared with Gilo or Shum (Fig. 4B).

Nucleotide diversity (π) of all the genotypes was determined to be 3.58 × 10^−3^ for whole genomes, 2.06 × 10^−3^ for genic regions, and 3.75 × 10^−3^ for intergenic regions. Nucleotide diversity for each genotype revealed lower diversity for Gilo (*S. anguivi*: 3.16 × 10^−3^, Shum: 3.65 × 10^−3^, and Gilo: 2.55 × 10^−3^, respectively). Linkage disequilibrium (LD) estimation using Haploview (version 4.2) [37] revealed that *r*^2^ reached the half maximum value at ∼150 kb (Fig. 4C), which is smaller than in other Solanaceae crops, e.g., tomato (2,000 kb) [38]. Because *S. aethiopicum* has been routinely used to improve disease resistance in eggplant and other Solanaceaee crops [14], we further identified SNPs that were strongly associated with resistance genes by selecting those lying within resistance genes. A total of 34,171 SNPs were finally selected, which could be used in the selection of Solanaceae plants with disease resistance (Supplementary Table 16).

### Population structure and demography of *S. aethiopicum*

**Figure 5:**
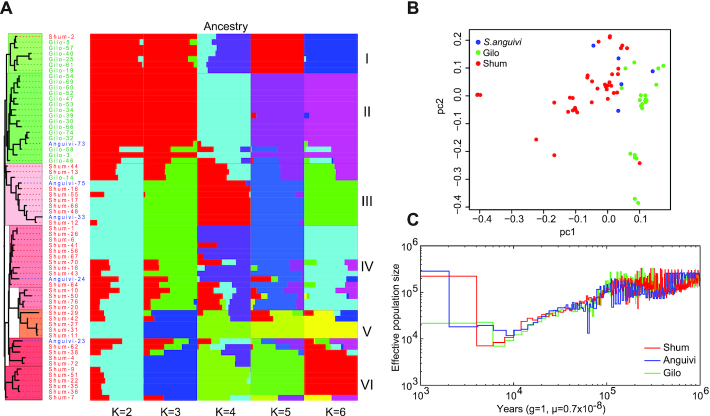
Population structure and demographic history of *Solanum aethiopicum*. (**A**) A maximum-likelihood phylogenetic tree and population structure constructed using the full set of single-nucleotide polymorphisms (SNPs). (**B**) Principal component analysis (PCA). (**C**) Pairwise sequential Markovian coalescent model analysis indicated a distinct demographic history of *S. aethiopicum* from 10,000 to 100 years ago, in which a bottleneck was shown ∼4,000–5,000 years ago, followed by an immediate expansion of population size.

To investigate the evolution and population demography of *S. aethiopicum*, we first built a maximum-likelihood (Fig. 5A, Supplementary Fig. 4) phylogenetic tree using the full set of SNPs. We observed population structure in the genome-wide diversity. As anticipated, the accessions from Gilo and Shum were clearly separated in the tree, with only 1 exception in each group, probably caused by labelling errors. On the other hand, accessions of *S. anguivi*, the known ancestor of *S. aethiopicum*, did not cluster separately, but grouped with either Gilo or Shum. This structure was also supported by principal component analysis (PCA), which clearly separated these accessions into 2 clusters (Fig. 5B, Supplementary Fig. 5).

The domestication history of *S. aethiopicum* was inferred by constructing a multilevel population structure using ADMIXTURE [39]. This enabled us to estimate the maximum-likelihood ancestry (Fig. 5A). The parameter K, representing the number of subgroups to be divided, was set in the range of 2–9, and the cross-validation (CV) error was calculated individually. The CV error converged to 0.4375 when K = 6, suggesting the division of the resequenced accessions into 6 subgroups: I–VI (Fig. 5A). The structure changes with increasing K-value from 2 to 6, showing a time-lapse domestication history of *S. aethiopicum* that was first split into 2 groups, Gilo and Shum. The former was subsequently divided into subgroups I and II. Two groups emerged in Shum when K = 3, each of which was then divided into 2 subgroups when K = 6. In summary, Gilo was divided into 2 subgroups (I and II) and Shum was divided into 4 subgroups (III–VI).

The demographic history of *S. aethiopicum* was inferred using the pairwise sequential Markovian coalescent model [40]. By doing this, we inferred changes in the effective population sizes of *S. aethiopicum* (Fig. 5C). Our data revealed distinct demographic trends from 10,000 to 100 years ago, in which a bottleneck was shown ∼4,000–5,000 years ago, followed by an immediate expansion of population size. The great population expansion might be associated with the early domestication of *S. aethiopicum* in Africa because it coincides with human population growth in western Africa, also occurring 4,000–5,000 years ago [41].

### Artificially selected genes in *S. aethiopicum*

We used reduce of diversity (ROD) and fixation index-statistics (Fst) measures to detect artificially selected regions along the genome. Briefly, ROD and Fst were calculated in a sliding non-overlap 10-kb window. Regions with ROD > 0.75 and Fst > 0.15 were identified as candidate regions under selection. As a result, genomic regions of 3,238 and 1,062 windows were found to be under selection during the domestication of Gilo and Shum, respectively (Supplementary Table 17). Among them, 161 windows were common between these 2 groups, while 3,077 and 901 windows were unique to Gilo and Shum, respectively. Genes located within these regions were identified as selected genes. Thirty-six and 1,406 selected genes were identified in Shum and Gilo, respectively, and 12 of these genes were selected in both. Ten of the 12 genes were annotated in the SwissProt database with known functions and included many genes known to be involved in tolerance to unfavorable environmental stresses, such as autophagy-related gene 18f (*ATG18f*), ATP-binding cassette transporter B (*ABCB18*), lysine–tRNA ligase (*LYSRS*), acyl-coenzyme A oxidase 4 (*ACX4*), inositol hexakisphosphate and diphosphoinositol-pentakisphosphate kinase (*VIP2*) (Supplementary Table 18). For example, ATG18 is reported to be involved in defense response to powdery mildew fungus through autophagy in *Arabidopsis* [42]; it is also involved in response to nutrition starvation by serving as an accessory component to ATG1/13 kinase complex [43]. ABCB is reported to be associated with lipid transport and confers tolerance to heavy metal ions, such as aluminium [44], cadmium, and lead [45]. The expression of *LYSRS* has been shown to be specifically induced in tomato root during the unusual accumulation of metal ions [46]. *VIP2* is reported to be critical in myo-inositol phosphate signalling pathways, and is known to be involved in responses to drought and salt stresses [47]. Furthermore, 2 genes encoding pentatricopeptide repeat-containing protein were also found among these genes, suggesting that RNA editing may have played a crucial role in the domestication of *S. aethiopicum* [48]. GO enrichment analysis showed that genes selected in both the Gilo and Shum groups were enriched in “transport” (Supplementary Table 19). GO terms for “response to auxin," “response to hormone," “response to salt stress” and “response to water” were also overrepresented in genes selected either in Gilo or Shum only. This result could explain the enhanced tolerance to drought and salinity in *S. aethiopicum*.

We also focused on the diversity of genes co-localized with LTR-Rs. A total of 24,682 SNPs were located within these co-localized genes, corresponding to 0.133% of the total number of SNPs (18,614,838). This is substantially fewer than would be expected if SNPs were evenly distributed across all genes, particularly because the LTR-R co-localized genes comprise 3.31% of the total gene set. The repellant of SNPs in these genes suggests purifying selection, which was also supported by the large amount (9,728; 39.41%) of rare SNPs (minor allele frequency <5%) found among the co-localized genes. We also observed that nonsynonymous SNPs (9,544) were much more abundant than synonymous ones (5310) among the co-localized genes. These variations led to amino acid changes in the encoded proteins, which may have contributed to the diversification of resistance genes.

### Pan- and core-genome of *S. aethiopicum*

Gene content varies across different accessions. A single reference assembly is insufficient to include all *S. aethiopicum* genes. Therefore, we assembled contigs for individual accessions using pair-end reads, with coverages ranging from 30 to 60× (Supplementary Table 20).

We assembled the genomes individually using SOAPdenovo2 [49] and filtered out contigs smaller than 2 kb. As a result, 753,084 contigs were retained, among which 432,785 were from Shum, 260,119 were Gilo and 60,180 were from *S. anguivi*. These contigs were further pooled separately and cleaned by removing duplicates using CD-HIT [50]. This led to the retention of 97,429, 76,638, and 36,915 contigs for Shum, Gilo and *S. anguivi*, respectively. The annotation of these contigs resulted in 41,626, 33,194, and 17,662 protein-coding genes, among which we identified accessory gene sets of 29,389, 23,726, and 12,829 for Shum, Gilo, and *S. anguivi*, respectively, by comparing against the reference genome sequence. We generated a pan-genome of *S. aethiopicum* (including Shum, Gilo and *S. anguivi* groups) of 51,351 genes (Supplementary Table 21). These genes were further clustered together with those annotated in the reference using CD-HIT. Overall, we identified 7,069 genes unique to the pan-genome gene set, suggesting that they had been missed from the reference. The average length of accessory genes was 1.62 kb with 2.22 introns. This is comparable to gene models in the reference genome, providing further evidence of accurate annotation. We further assigned their putative functions by querying against protein databases. A total of 48,572 (94.59%) genes were fully annotated and functional descriptions (Supplementary Table 22) provided. Among the identified gene models, 10,409 (20.27%) were common to these 3 groups and were thus defined as “core” genes. As expected, they were mainly composed of housekeeping genes (Supplementary Table 23). However, it is important to note that the number of core genes may have been underestimated because *S. anguivi* was underrepresented, while the other 2 *S. aethiopicum* groups, Kumba and Aculeatum, were not included in the present study.

## Discussion

*Solanum aethiopicum* is cross-compatible with *S. melongena* and is routinely used as a donor of disease resistance genes to its close relative [14]. Genomic analysis of *S. aethiopicum* revealed higher LTR-mediated expansion of resistance gene families than its other close relatives, including tomato, potato, eggplant, and hot pepper. LTR amplification is one of the major forces driving genome evolution. It shapes the genome by capturing, interrupting, or flanking genes [51]. The consequences of LTR insertions depend on the genomic position of insertion. For example, inserting into protein-coding sequences results in pseudogenization. LTR-Rs adjacent to protein-coding genes can downregulate or silence the expression of flanking genes by extending methylation regions or by producing antisense transcripts [52–55]. LTR-Rs also mediate gene retroposition, capturing genes back into the genome [51]. In the present study, LTRs preferentially captured genes related to disease resistance, resulting in the overrepresentation of GO terms related to disease resistance in the LTR-captured genes. Enrichment of the GO terms “chitin binding (GO:0008061)” and “chitinase activity (GO:0006032)” (Fig.   3B, Supplementary Table 12) implies that these genes may have been selected to resist infection by fungal pathogens, such as *Fusarium oxysporum* [56]. On the contrary, no GO term enrichment was seen in genes that were disrupted by LTR-Rs. This suggests that gene disruption by LTR-Rs may be a random event in terms of gene function. The age distribution of LTR-R captured genes coincidently fit with that of the LTR-R–disrupted genes, suggesting that these 2 events may have occurred simultaneously (Fig. 3A). It is not clear why genes related to disease resistance were favoured by LTR-Rs, but one explanation is that the disease resistance genes may have been more active than other genes at the time of LTR retrotransposition. The expression pattern of LTR-R captured genes also varied between tissues. Those related to resistance were specifically active in the leaf, while those engaged in the transport of cations, nitrogen, and cell proliferation were active in flowers. This outcome suggests low abundance of transcripts for disease resistance genes, resulting in a relatively low chance to adequately capture the genes in flowers under normal conditions. Another possible scenario is that LTR retrotransposition occurred under stress conditions, which resulted in the simultaneous induction of the expression of resistance genes in gametes and the activity of LTR retrotransposition. Such possible stresses might be extreme environmental conditions or pathogen infection. A “reinforcement model” has been proposed to explain the simultaneous accumulation of stress-responsive genes and the activity of retrotransposons in genomes under environmental stress [57, 58].

There are 4 major groups of *S. aethiopicum*: “Gilo," “Shum," “Kumba,” and “Aculeatum.” We resequenced accessions from the Gilo and Shum groups, which are widely consumed as vegetables. The accessions resequenced in this study were clustered into 6 subgroups (4 for Shum and 2 for Gilo). By scanning for regions with lower genomic diversity, we identified regions and several genes involved in responses to salt, water, and drought tolerance that were under selection during the domestication of *S. aethiopicum*. Furthermore, purification selection was also found among disease resistance genes.

In the present study, resequencing *S. aethiopicum* and *S. anguivi* genomes at a high depth (30–60×) (Supplementary Table 20) enabled us to assemble draft genomes for these individuals. Despite resequencing only a few genotypes from the 2 groups, we intend to supplement the reference gene set with accessory genes by pooling the resequenced contigs for gene prediction and annotation. This “pan-genome” is expected to provide a more comprehensive understanding of *S. aethiopicum* in the future.

We report a reference genome for African eggplant, which will provide a basic data resource for further genomic research and breeding activities for *S. aethiopicum*. The gene sequences annotated in the genome will be essential for developing genome editing vectors to create mutants to further understand the functions of genes within the genome and develop superior genotypes. Molecular markers developed using the genome sequences will also enable more efficient and precise selection of superior accessions by breeders.

## Methods

### DNA extraction, library construction and sequencing, and genome assembly

High molecular weight genomic DNA was extracted from young leaves of 14-day-old seedlings of *Solanum aethiopicum* “Shum” accession 303, which had been previously and repeatedly selfed to ensure homozygosity. Shum 303 is a selection of African eggplant from Uganda, with green fruits and pigmented stem and leaf veins. DNA was extracted using a modified cetyl trimethylammonium bromide (CTAB) protocol, as previously described [59]. Briefly, 2.5 g fresh leaf tissue was flash-frozen in liquid nitrogen and ground to a fine powder, before adding 15 mL of 2× extraction buffer (100 mM Tris-HCl pH 8.0, 1.4 M NaCl, 20 mM EDTA, 2% w/v CTAB, 10 µL/mL β-mercaptoethanol), then incubated at 65°C. One volume of chloroform: isoamyl alcohol (24:1) was added and mixed and the sample was centrifuged twice. The aqueous phase was precipitated overnight and the washed pellet was treated with RNaseA. A repeat chloroform extraction was performed, as above, to remove RNaseA and any other contaminants. The aqueous phase was collected and DNA was precipitated and washed with ethanol. DNA was allowed to dry, then was resuspended in 100 μL elution buffer.

High molecular weight DNA was fragmented and used to construct paired-end libraries with insert sizes of 250 bp, 500 bp, 2 kb, 6 kb, 10 kb, and 20 kb, following standard Illumina protocols. The libraries were sequenced on an Illlumina HiSeq 2000 platform, resulting in a total of 242.61 Gb raw reads. Filtering of duplicated, low-quality reads and reads with adaptors was done using SOAPfilter (version 2.2, an application included in the SOAPdenovo2 package, RRID:SCR_014986) [49] with the parameters “-M 2, -f 0, -p”. Reads with ≥40% low-quality bases or with ≥10% uncalled bases (“N”) were filtered. We used 17 *k*-mer counts [21] of high-quality reads from small insert libraries to evaluate the genome size and heterozygosity using GCE [60] and Kmergenie [61]. We assembled the genome using Platanus (Platanus, RRID:SCR_015531) [22].

Genomic DNA used for resequencing was extracted from young leaves of 65 accessions. DNA was sheared into small fragments of ∼200 bp and used to construct paired-end libraries, following standard BGI protocols as previously described [62], and subsequently sequenced on a BGI-500 sequencer. Briefly, the DNA fragments were ligated to BGISEQ-500 compatible adaptors, followed by an index PCR amplification, the products of which were then pooled and circularized for sequencing on the BGISEQ-500 (BGI, Shenzhen, China). Ultra-deep data were produced for each accession, with coverage ranging from ∼45 to ∼75× (Supplementary Table 20).

### RNA extraction, library construction, and sequencing

For RNA extraction, seeds of Gilo and Shum inbred lines were obtained from Uganda Christian University. The seeds were planted in a screenhouse at the BecA-ILRI Hub (Nairobi, Kenya) in polyvinylchloride pots (13 cm height and 11.5 cm diameter) containing sterile forest soil and farmyard manure (2:1). The seedlings were later transplanted into larger polyvinylchloride pots of 21 cm height and 14 cm diameter. Plants were raised in a screenhouse at 21–23°C and 11–13°C day and night temperatures, respectively (average 12 light hours per day). The plants were regularly watered to maintain moisture at required capacity.

Two plants were selected randomly from each of Gilo and Shum accessions and were tagged at the seedling stage for tissue sampling. Fresh tissues were sampled from each of the tagged plants and flash-frozen in liquid nitrogen immediately. Total RNA was extracted from the frozen tissues using the ZR Plant RNA MiniprepTM Kit (Zymo Research, Irvine, CA, USA), according to the manufacturer's instructions. RNA integrity was evaluated by electrophoresis in denaturing agarose gel (1% agarose, 5% formamide, 1× TAE) stained with 3× GelRed (Biotium, Fremont, CA, USA). RNA was quantified using the Qubit RNA Assay Kit (Thermo Fisher Scientific, Carlsbad, CA, USA). Ribosomal RNA (rRNA) was removed from 4 µL of total RNA from each sample using the Epicentre Ribo-zero™ rRNA Removal Kit (Epicentre, Madison, WI, USA). The rRNA-depleted RNA was then used to generate strand-specific RNA-seq libraries using TruSeq® Stranded mRNA Kit (Illumina, San Diego, CA, USA). Twenty mRNA libraries were prepared, multiplexed (10 samples at a time), and sequenced as paired-end reads on the MiSeq (Illumina, San Diego, USA) platform at the BecA-ILRI Hub. Similar to the process of filtering genomic reads, SOAPfilter software [49] was used, with the parameters “-M 2, -f 0, -p” to filter low-quality reads and adaptor sequences. Reads with ≥40% low-quality bases or with ≥10% uncalled bases (“N”) were filtered out.

### Repeat annotation

Tandem repeats were searched in the genome using TRF, version 4.04 [63]. Transposable elements (TEs) were identified by a combination of homology-based and *de novo* approaches. Briefly, the assembly was aligned to a known repeats database (Repbase16.02) using RepeatMasker (RRID:SCR_012954) and RepeatProteinMask (version 3.2.9) [64] at both the DNA and protein level. In the *de novo* approach, RepeatModeler (version 1.1.0.4, RRID:SCR_015027) [65] was used to build a *de novo* repeat library using the *S. aethiopicum* assembly, in which redundancies were filtered out. TEs in the genome were then identified by RepeatMasker [64]. LTRs were identified using LTRharvest [29], with the criterion of 75% similarity on both sides. LTRdigest [30] was used to identify the internal elements of LTR-Rs with the eukaryotic tRNA library [66]. Identified LTR-Rs including intact poly purine tracts and primer binding sites with LTR-Rs on both sides were considered to be the final intact LTR-Rs. These were then classified into superfamilies, *Gypsy*and *Copia*, by querying against Repbase 16.02 [67].

### Annotation of gene models and ncRNA

Gene models were predicted using a combination of *de novo* prediction, homology search, and RNA-aided annotation. Augustus software (RRID:SCR_008417) [68] was used to perform *de novo* prediction after the annotated repeats were masked in the assembly. To search for homologous sequences, protein sequences of 4 closely related species (*S. lycopersicum, S. tuberosum, Capsisum annuum*, and *Nicotiana sylvestris*), together with *Arabidopsis thaliana*, were used as query sequences to search the reference genome sequence using TBLASTN (RRID:SCR_011822) [69] with the e-value ≤1e−5. Regions mapped by these query sequences were subjected to GeneWise (RRID:SCR_015054) [70], together with their flanking sequences (1,000 bp) to identify the positions of start/stop codons and splicing. For RNA-aided annotation, RNA-seq data from different tissues of *S. aethiopicum* were mapped to the genome assembly of *S. aethiopicum* using HISAT (RRID:SCR_015530) [71]. Mapped reads were then assembled using StringTie (RRID:SCR_016323) [72]. GLEAN software [73] was used to integrate mapped transcripts from different sources to produce a consensus gene set. tRNAscan-SE (RRID:SCR_010835) [74] was performed to search for reliable tRNA positions. snRNA and miRNA were detected by searching the reference sequence against the Rfam database (RRID:SCR_007891) [75] using BLAST [69]. rRNAs were detected by aligning with BLASTN (RRID:SCR_004870) [69] against known plant rRNA sequences [76]. For functional annotation, protein sequences were searched against Swissprot, TrEMBL, KEGG (release 88.2), InterPro, Gene Ontology, COG, and Non-redundant protein NCBI databases [77–82].

### Gene family analysis

Proteins of *S. aethiopicum, S. tuberosum* (PGSC v3.4) [18], *S. lycopersicum* (v2.3) [19], *C. annuum* (PGA v.1.6) [24], and *S. melongena* (Sme2.5.1) [83] were selected to perform all-against-all comparisons using BLASTP (RRID:SCR_001010) [69], with an e-value cutoff of ≤1e−5. OrthoMCL (RRID:SCR_007839) [26] and the default MCL inflation parameter of 1.5 were used to define the gene families. Single-copy families were selected to perform multiple sequence alignment using MAFFT (RRID:SCR_011811) [84]. Four-fold degenerate sites were picked and used to construct a phylogenetic tree based on the maximum-likelihood method by PhyML (RRID:SCR_014629) [85], with *C. annuum* as the outgroup. WGD analysis was achieved by identifying colinearity blocks by paralog gene pairs in MCscanX, with default parameters [27]. Each aligned paralog gene pair was concatenated to a super-sequence in 1 colinearity block and 4dTv (transversion of 4-fold degenerate site) values of each block were calculated. We also determined the distribution of 4DTv values to estimate the speciation between species or WGD events. The divergence time of *S. aethiopicum* was estimated using the MCMCtree program [86], with the constructed phylogenetic trees and the divergence time of *C. annuum* [24] and *S. tuberosum* [18].

### Analysis of LTR-Rs

Insertion times of identified, intact LTR-Rs were estimated on the basis of the sequence divergence between the 5′ and 3′ LTR of each element. The nucleotide distance *K* between 1 pair of LTR-Rs was calculated using the Kimura 2-parameter method in Distmat (EMBOSS package) [87]. An average base substitution rate of 1.3e−8 [31] was used to estimate the insertion time, based on the following formula:

*T* = *K */2*r* [15].

Transcriptomic data were used to analyse the activity of intact LTR-Rs. After filtering and removing low-quality reads, high-quality reads from each were mapped against the full-length LTR-R sequence using BWA-MEM software [88], with default parameters. Expression levels of intact LTR-Rs were calculated using EdgeR [89] and visually presented using pheatmap in R [90].

### Analysis of NB-containing genes

NB domain-containing genes in the *S. aethiopicum* genome were identified using a method previously described [15, 91]. Briefly, the hidden Markov model (HMM) profile of the NB-ARC domain (PF00931) was used as a query to perform an HMMER search (version 3.2.1, RRID:SCR_005305 [92]) against protein sequences of tomato, potato, hot pepper [18, 19, 24], and annotated sequences of *S. aethiopicum*, with an e-value cut-off of ≤1e−60. Aligned NB-ARC domain sequences of *S. aethiopicum* were extracted and used to build the *S. aethiopicum*–specific HMM model. NB-ARC domain sequences of tomato, potato, and hot pepper were mapped as the query sequences against the *S. aethiopicum* genome using TBLASTN [69], with an e-value cut-off of ≤1e−4 using GeneWise software [70] to identify candidate NB-containing genes at the whole-genome level. Final NB-containing genes were confirmed by searching the genome with an *S. aethiopicum*–specific NB-ARC HMM model, constructed with an e-value cut-off of ≤1e−4. Retroduplicated NLRs were identified according to the method described by Kim et al. (2017) [15]. Phylogenetic trees for *S. aethiopicum* and *S. melongena* NB-containing genes were constructed using FastTree (RRID:SCR_015501) [93], with default parameters.

### SNP calling

The GATK pipeline (RRID:SCR_001876) [94] was used to call SNPs and indels. Briefly, low-quality, duplicated, and adaptor-contaminated reads were filtered using SOAPfilter (version 2.2) [49] before further processing. To reduce the compute time, scaffolds in the assembly were sequentially linked into 24 pseudo-chromosomes, in which the original scaffolds were separated by 100 Ns, before mapping reads using BWA (RRID:SCR_010910) [88], with default parameters. Picard Tools [95] and SAMtools (RRID:SCR_002105) [96] were used to further process the alignment outputs, including sorting and marking of duplicates. After alignment and sorting, the GATK pipeline (version 4.0.11.0) was used to call SNPs by sequentially implementing the following modules: RealignerTargetCreator, IndelRealigner, UnifiedGenotyper, samtools mpileup, VariantFiltration, BaseRecalibrator, AnalyzeCovariates, PrintReads, and HaplotypeCaller, with default parameters. This pipeline produced a file in gvcf format, which displayed the called SNPs and indels filtered according to genotype information. The file was then analysed using PLINK software [97] for quality control, with “GENO>0.05, MAF<0.1, HWE test p-value ≤0.0001” parameters (GENO: maximum per-SNP missing; MAF: minor allele frequency; HWE: Hardy-Weinberg disequilibrium *P*-value). The loci of these SNPs and indels were anchored back to the original scaffolds and annotated using SnpEff [98]. To identify structural variations (SVs), sample information was added using AddOrReplaceReadGroups, a module of Picard-tools, and SVs were detected using DiscoverVariantsFromContigAlignmentsSAMSpark, a GATK module.

### Population analysis

A maximum-likelihood phylogenetic tree was constructed, based on the genotypes at all the SNP loci using FastTree [93], with default parameters. To perform PCA, Beagle4.1 [99] was used to impute the unphased genotypes. All imputed and identified genotypes at SNP loci were pooled and finalized using PLINK [97] and ReSeqTools [100], which were then subjected to PCA using GCTA software [101]. The population was clustered using ADMIXTURE software [39], with K (the expected number of clusters) increasing from 2 to 9. The K value with the minimum cross-validation error was eventually selected.

Genome-wide linkage disequilibrium (LD) was calculated for populations of different groups using Haploview [102] in windows of 2,000 kb. Briefly, the correlation coefficient (*r*^2^) between SNP pairs in a non-overlapping sliding 1-kb bin was calculated and then averaged within bins.

Candidate regions under selection were identified by comparing polymorphism levels—measured by ROD, as well as by *F*_ST_—between Gilo, Shum, and *Solanum anguivi* groups. ROD was calculated using the formula

$$\begin{equation*}
\mathrm{ROD} = 1-{\pi _{{\rm{cul}}}}/{\pi _{{\rm{wild}}}},
\end{equation*}
$$

where π_cul_ and π_wild_ denote the nucleotide diversity within the cultivated and wild populations, respectively.

*F*_ST_ measurement was calculated according to the formula

$$\begin{equation*}
{F_{\mathrm{ST}}} = \left( {{\pi _{{\rm{between}}}} - {\pi _{{\rm{within}}}}} \right)/{\pi _{{\rm{between}}}},
\end{equation*}
$$

where π_between_ and π_within_ represent the average number of pairwise differences between 2 individuals sampled from different or the same population.

### Construction of pan- and core-genome

To build a gene set including as many *S. aethiopicum* genes as possible, we assembled contigs of all 65 resequenced accessions individually using SOAPdenovo2 [49]. The assembled contigs from each group (Gilo, Shum, and *S. anguivi*) were then merged. CD-HIT-EST [50] was used to eliminate redundancy and generate the final dataset of pan-genomes for each group. Similarly, all these contigs were merged into a pan-genome of *S. aethiopicum*. Gene models were predicted from these contigs as described above and their functions were also annotated.

## Availability of supporting data and materials

The raw sequence data from our genome project were deposited in the NCBI SRA with BioProject number PRJNA523664 and in the CNGB Nucleotide Sequence Archive database under project accession number CNP0000317. Assembly and annotation of the *S. aethiopicum* genome are available in GigaDB [103].

## Additional files

Supplementary Table 1. Summary of the library types and data generated in this study

Supplementary Table 2. Statistics of the *S. aethiopicum* assembly

Supplementary Table 3. Comparison of the genomic characteristics in different genomes

Supplementary Table 4. Statistics of repeat annotation, transposable elements

Supplementary Table 5. Statistics of gene model in different species

Supplementary Table 6. Evaluation of predicted gene models using 1,440 CEGs

Supplementary Table 7. Statistics of predicted ncRNA

Supplementary Table 8. Result of function annotation

Supplementary Table 9. Statistics of gene families

Supplementary Table 10. Common shared gene families between *S. aethiopicum* and other species

Supplementary Table 11. GO annotation of expansion gene family of *S. aethiopicum*

Supplementary Table 12. GO classification and enchriment analysis of LTR-captured and LTR disrupted genes in *S. aethiopicum*

Supplementary Table 13. GO classification and enchriment analysis of LTR capture genes with specifical activity in different tissues

Supplementary Table 14. GO classification and enchriment analysis of total specifical active gene in 4 tissues

Supplementary Table 15. Number of annotated NLR genes in *S. aethiopicum, S. melongena* and other species

Supplementary Table 16. SNPs within resistance genes

Supplementary Table 17. Under selection genomic regions of Gilo and Shum

Supplementary Table 18. Gene position and functional annotation description for 12 Artificially selected genes (both in Gilo and Shum)

Supplementary Table 19. GO enrich for Artificially selected genes (Gilo and Shum)

Supplementary Table 20. Sequencing data of Pan-genome

Supplementary Table 21. Pan-genome annotation

Supplementary Table 22. Statistics of functional annotation for the protein-coding genes from the three groups (*S. anguivi*, Shum and Gilo) and pan-genome (All)

Supplementary Table 23. Statistics of genes which commened shared by three groups (*S. anguivi*, Gilo and Shum)

Supplementary Figure 1. 17-mer analysis for estimating the *S. aethiopicum* genome size

Supplementary Figure 2. Distributions of the gene model for four categories in the relative species

Supplementary Figure 3. Distinct expression pattern of LTR-Rs and their captured genes in different tissues

Supplementary Figure 4. Maximum-likelihood phylogenetic tree of 65 samples using the full-set of SNPs

Supplementary Figure 5. Principal-component analysis

giz115_GIGA-D-19-00026_Original_Submission

giz115_GIGA-D-19-00026_Revision_1

giz115_Response_to_Reviewer_Comments_Original_Submission

giz115_Reviewer_1_Report_Original_Submission Cyril Jourda -- 3/25/2019 Reviewed

giz115_Reviewer_1_Report_Revision_1 Cyril Jourda -- 7/19/2019 Reviewed

giz115_Reviewer_2_Report_Original_Submission Kenta Shirasawa -- 4/3/2019 Reviewed

giz115_Reviewer_3_Report_Original_Submission Lorenzo Barchi -- 4/8/2019 Reviewed

giz115_Reviewer_3_Report_Revision_1 Lorenzo Barchi -- 7/15/2019 Reviewed

giz115_Supplemental_Files

## Abbreviations

4DTV: 4-fold degenerative third-codon transversion; BLAST: Basic Local Alignment Search Tool; bp: base pairs; BUSCO: Benchmarking Universal Single-Copy Orthologs; BWA: Burrows-Wheeler Aligner; CEG: core embryophyta gene; COG: Clusters of Orthologous Groups; CV: cross-validation; Fst: fixation distance; GATK: Genome Analysis Toolkit; Gb: gigabase pairs; GC: guanine-cytosine; GCE: Genomic Character Estimator; GCTA: Genome-wide Complex Trait Analysis; GO: gene ontology; HMM: hidden Markov model; kb: kilobase pairs; KEGG: Kyoto Encyclopedia of Genes and Genomes; LTR: long terminal repeat; LD: linkage disequilibrium; LTR-R: long terminal repeat retrotransposon; MAFFT: Multiple Alignment using Fast Fourier Transform; Mb: megabase pairs; MYA: million years ago; NB-LRR: nucleotide-binding, leucine-rich repeat domain; NCBI: National Center for Biotechnology Information; NLR: nucleotide-binding, leucine-rich repeat-related; PCA: principal component analysis; RNA-seq: RNA-sequencing; rRNA: ribosomal RNA; SNP: single-nucleotide polymorphism; snRNA: small nuclear RNA; SRA: Sequence Read Archive; TE: transposable element; TrEMBL: Translation of European Molecular Biology Laboratory; TRF: Tandem Repeats Finder; tRNA: transfer RNA; WGD: whole-genome duplication.

## Competing interests

The authors declare that they have no competing interests.

## Funding

This work was supported by the National Natural Science Foundation of China (grant number 31601042), the Science, Technology and Innovation Commission of Shenzhen Municipality (grant numbers JCYJ20151015162041454 and JCYJ20160331150739027), and by the Guangdong Provincial Key Laboratory of Genome Read and Write (grant number 2017B030301011).

## Authors' contributions

D.A.O., X.X., A.V., X.Liu., H.S., R.J., A.M., J.W., and H.Y. conceived the project; D.A.O., F.S., E.B.K., A.V., S.C., and H.L. managed and supervised the work; B.S., Y.F. and X.C. managed the samples at BGI; B.S., Y.F. and W.C. assembled the whole genome; and Y.F., Y.S., and W.S. annotated the genome. S.N.K., S.M., and R.K. extracted high molecular weight DNA. H.L. and S.P. constructed DNA libraries and sequenced the genome. S.N.K. and S.M. prepared RNA libraries and sequenced the transcriptome. J.N. and S.N.K. assembled and analysed the transcriptome. Y.S., X.Li. and B.S. performed the analysis of gene families, LTR evolution, and transcriptomic data; P.N.K. extracted DNA for resequencing samples. Y.F. and B.S. analysed the resequencing data; Y.S., Y.F., S.W. and B.S. collected datasets required for the genome annotation and analyses. B.S., X.Liu., Y.S., D.A.O., P.S.H. and Y.F. wrote and revised the manuscript.
