# Supplementary material for: Draft genome sequence of Solanum aethiopicum provides insights into disease resistance, drought tolerance, and the evolution of the genome
Source: Gigascience. 2019 Oct 1;8(10):giz115. doi: 10.1093/gigascience/giz115 (PMC6771550; doi:10.1093/gigascience/giz115)
Supplement: giz115_GIGA-D-19-00026_Revision_1 [file giz115_giga-d-19-00026_revision_1.pdf]

GigaScience

Draft genome sequence of the Solanum aethiopicum provides insights into disease resistance, drought tolerance and the evolution of the genome

--Manuscript Draft--

Manuscript Number:	GIGA-D-19-00026R1
Full Title:	Draft genome sequence of the Solanum aethiopicum provides insights into disease resistance, drought tolerance and the evolution of the genome
Article Type:	Research
Funding Information:	National Natural Science Foundation of China (No. 31601042) — Dr. Bo Song<br>Science, Technology and Innovation Commission of Shenzhen Municipality (JCYJ20151015162041454) — Not applicable<br>Science, Technology and Innovation Commission of Shenzhen Municipality (JCYJ20160331150739027) — Not applicable<br>Guangdong Provincial Key Laboratory of Genome Read and Write (No. 2017B030301011) — Dr. Xin Liu
Abstract:	Background<br>The African eggplant (Solanum aethiopicum) is a nutritious traditional vegetable used in many African countries, including Uganda and Nigeria. It is believed to have been domesticated in Africa from its wild relative, Solanum anguivi. S. aethiopicum has been routinely used as a source of disease resistance genes for several Solanaceae crops including Solanum melongena. Breeding of S. aethiopicum has lagged behind due to lack of genomic resources.<br>Results<br>We assembled a 1.02 Gb draft genome of S. aethiopicum, which contained predominantly repetitive sequences (76.2%). We annotated 37,681 gene models including 34,906 protein-coding genes. We observed an expansion of disease resistance genes through two rounds of amplification of long terminal repeat retrotransposons (LTR-Rs), which may have occurred around 1.25 and 3.5 million years ago, respectively. We identified 14,995,740 SNPs by re-sequencing 65 S. aethiopicum and S. anguivi genotypes, of which 41,046 SNPs were closely linked to disease resistance genes. The domestication and demographic history analysis revealed the active selection for genes involved in drought tolerance in both “Gilo” and “Shum” groups. A pan-genome of S. aethiopicum with a total of 51,351 protein-coding genes was assembled, 7,069 genes of which are missing in the reference genome.<br>Conclusions<br>The genome sequence of S. aethiopicum enhances our understanding of its extraordinary biotic and abiotic resistance nature. The SNPs identified will be available for immediate use by breeders. The information provided here will greatly accelerate the selection and breeding of the African eggplant as well as other crops within the Solanaceae family.
Corresponding Author:	Xin Liu, Ph.D.<br>BGI<br>CHINA
Corresponding Author Secondary Information:	
Corresponding Author's Institution:	BGI
Corresponding Author's Secondary Institution:	
First Author:	Bo Song
First Author Secondary Information:	

Powered by Editorial Manager® and ProduXion Manager® from Aries Systems Corporation

Order of Authors:	Bo Song
	Yue Song
	Yuan Fu
	Elizabeth Balyejusa Kizito
	Pamela Nahamya Kabod
	Sandra Ndagire Kamenya
	Huan Liu
	Samuel Muthemba
	Robert Kariba
	Xiuli Li
	Sibo Wang
	Shifeng Cheng
	Alice Muchugi
	Ramni Jamnadass
	Howard Yana-Shapiro
	Allen Van Deynze
	Huanming Yang
	Jian Wang
	Xun Xu
	Damaris Achieng Odeny
	Xin Liu, Ph.D.
Order of Authors Secondary Information:	
Response to Reviewers:	Reviewer reports:<br><br>Reviewer #1: The manuscript entitled "Draft genome sequence of the Solanum aethiopicum provides insight into disease resistance, drought tolerance and evolution" is a genomic study of Solanum aethiopicum, a close relative of the cultivated eggplant Solanum melongena.<br>Methods are very appropriate to the aims of the study and conclusions are adequately supported by the genomic data.<br><br>Could you give more details about the method of:<br>- The high molecular genomic DNA extraction?<br>Response: More details and the cited reference were added.<br>- The selection of high-quality reads?<br>Response: Details have been added.<br><br>- The multiplexing? (barcoding?) and the demultiplexing?<br>Response: The delivered reads were already demultiplexed.<br><br>- The identification of collinearity blocks (parameters of MCscanX)?<br>Response: Changed to "… gene pairs in MCscanX with default parameters".<br><br>- The RNAseq read filtering and removing of low-quality reads (tools, parameters and threshold)?<br>Response: Details have been added in the text. "SOAPfilter software with the parameters "-M 2, -f 0, -p" was used to filter low quality reads and adapter sequence. Also reads with >=40% low quality bases or with >=10% uncalled bases ("N") were filtered."

Powered by Editorial Manager® and ProduXion Manager® from Aries Systems Corporation

- The variant calling pipeline? (default parameters in GATK for SNP and SV?)
Response: Yes, we used default parameters in GATK pipeline for SNP and SV identified. For quality control, parameters “GENO>0.05, MAF<0.1, HWE test p-value <=0.0001” was used. Detailed parameters have been added.

- The pan-genome reconstruction (parameters and threshold of SOAPdenovo2 and CD-HIT-EST)?
Response: We use SOAPdenovo2 and CD-HIT-EST software to construct pan-genome with default parameters.

Minor comments:
- Could you describe the eggplant accession used to produce the genome assembly?
Response: A brief description had been added.

- You have used a substitution rate of 1.3e-8 year-1site-1 based on works performed on rice genomes. Could you justify this?
Response: Generally, the substitution rate varies little among different plants. For example, the substitution rate reported in Arabidopsis is 7 × 10−9 base substitutions per site per generation (Ossowski et al, 2010), which is quite close to that in rice. The use of the rate of rice enables the comparison between our study and another study of hot pepper, in which the same substitution rate was used to infer the ages of LTRs (Kim et al., 2017).

- Could you perform a statistical test to validate the comparison of degeneration of LTR-R activities in different tissues?
Response: Unfortunately, statistical test is not allowed without replicates. Instead, we added regression onto the plots.

- An amplification of LTR is found in Solanum aethiopicum and also in Solanum melongena. Could you give us the reference?
Response: We searched for LTR in S. melongena genome (Hirakawa et al., 2014) in this study. A same method and criteria were used in both the genomes so that the results are comparable.

- The number of SNP seems huge. Could you compare with others plant genomes? (Yuan Fu)
Response: In this study, we had identified 18,614,838 SNPs in total. The number of SNP is highly dependent on the variations between the accessions used in different studies. The differences of genome sizes also contribute to the varied number of SNP in different species. Actually, it is not fair to compare the number of SNPs between different species and populations. Take tomato, whose genome size (828 Mb) is comparable to S. aethiopicum, as an example, a number of 11,620,517 SNPs and 1,303,213 small indels were identified in a population of 360 accessions (Lin et al., 2014). Furthermore, it is not surprise to have such a large number of SNPs in S. aethiopicum because it is a hypervariable species (Lester et al., 1986).

- "Artificially selected genes", what does the term artificial mean? Could you explain/develop?
Response: It means the genes preferentially retained by human during the history of domestication.

- Numbers of accessory genes seem huge. Could you check if these values are not overestimate due to the presence of fragmented genes?
Response: The genome sequences per se varies greatly among different groups (Lester et al., 1986), several groups were previously recognized as different species. Although we cannot completely exclude the possibility of overestimation caused by the presence of fragmented genes, the degree of overestimation is minor because the length of CDS of accessory genes (921 bp) (Supplementary Table 20) is comparable to that of genes (1104 bp) (Supplementary Table 5) in reference.

- "Good quality transcripts" ", what does the term good mean? Could you explain/develop?
Response: It has been rephrased to “The mapped reads were then assembled using

Powered by Editorial Manager® and ProduXion Manager® from Aries Systems Corporation

StringTie”

- Could you justify the choice of e-value thresholds for gene annotations and gene clustering (1e-4 seems very weak)?
Response: The cutoff of 1e-4 was used for the identification of NLR. It is actually not that weak and had been used in many other studies (Seo et al., 2016 and Kim et al., 2017). Another reason we use this threshold is to make our results comparable to that reported in pepper (Kim et al., 2017), which used a threshold of e-value <=1e-4.

- Could you explain acronyms (GENO, MAF, HWE)?
Response: The full names have been added in the manuscript. They are GENO: Maximum per-SNP missing, MAF: Minor allele frequency, HWE: Hardy-Weinberg disequilibrium p-value.

Reviewer #2: This paper reports the first genome assembly of Solanum aethiopicum. The description is easy to follow and the data would be useful for the breeding programs of eggplant. I recommend the authors to submit the data (genome, genes, protein, annotatoin, sequence variations etc) to Sol Genomics Network <https://solgenomics.net> so that potential users can access them easily.
Response: Thanks. That’s a very good suggestion. We will arrange the submission upon the acceptance of the paper.

Minor comments:
The term "the reference genome" in the main text should be replaced by "the reference genome sequence".
Response: Replaced. Thanks.

Abstract: LTR-Rs should be spelled out.
Response: Replaced by “long terminal repeat retrotransposons (LTR-Rs)”. Thanks. (P2, L12)

Abstract: "closely" is ambiguous.
Response: It is 150 kb. It had been indicated in the text.

Introduction: "We also re-sequenced two ...". Is this 65 (not two) as mentioned in Abstract and other parts?
Response: Changed to “two groups”

Data Description: While a total of 242.6 Gb raw reads were obtained, only 127.83 Gb were used for assembly. I assume that approximately 115 Gb reads were low quality. Correct?
Response: Yes, the quality of several of the libraries were poor at the beginning of this work, therefore we added more libraries to make sure the final clean data is sufficient.

Data Description: Only 80.4% complete BUSCOs were found in the assembly, whereas the total length of the assembly was 1.02 Gb covering 87% of the estimated genome size (1.17 Gb). Please clarify the reason for the low BUSCOs. (Yuan Fu, please explain this)
Response: We won’t deny that this assembly is only a draft and there must be some genes and sequences missed. In order to keep only the most reliable predictions of gene models, we used much more stringent criteria for gene annotation, compared to many other studies on Solanaceae genomes, resulting in a smaller but more accurate gene set. For example, the genome of Solanum melongena has as many as 85,446 genes (Hirakawa et al, 2014). In fact, the scores of BUSCO assessment can be increased by relaxing the criteria for gene annotation. However, this will also include more inaccurate gene models. We had other version of gene sets with higher scores but we finally selected this one hoping to removing false annotations as many as possible.

Increased resistance is facilitated by LTR-Rs amplification: What is the definition of "LTR-Rs captured"? It is unclear why the "LTR-Rs captured" genes enhance disease resistance.

NLR?
Response: The genes located in LTR-Rs were defined as LTR-Rs captured genes. It is likely that these genes were retroposed by the retrotransposition of LTR-Rs. As these genes are overrepresented by NLRs, we speculate that they are beneficial to disease resistance.

Polymorphisms in different S. aethiopicum groups: What's the difference between indels and SVs?
Response: In this study, we follow the criteria described in the users' guide of GATK pipeline (version 4.0), in which SV is considered to be structural variant, while indel is defined as short variants including small deletion or insertions.

Artificially selected genes in S. aethiopicum: What types of selections do the authors mention here?
Response: They are the genes preferentially retained by human during the domestication of this crop.

Potential implications: This part can be deleted because this is not based on the data.
Response: removed.

Methods: What are the "standard BGI protocols"?
Response: Changed to "The DNA was sheared into small fragments of ~ 200 bp and used to construct paired-end libraries following standard BGI protocols as described in (Mak et al., 2017) and subsequently sequenced on a BGI-500 sequencer. Briefly, the DNA fragments were ligated to BGISEQ-500 compatible adapters, followed by an index PCR amplification, the products of which were then pooled and circularized for sequencing on BGISEQ-500 (BGI, Shenzhen, China).

SNP calling: "samtools mpileup" and "VariantFiltration" are duplicated.
Response: Corrected.

Reviewer #3: The manuscript describes a draft assembly and annotation for S. aethiopicum genome.
Authors estimated the repetitive elements content and proposed that two amplifications of LTR-Rs occurred around 1.25 and 3.5 million years ago, resulting in the expansion of resistance genes. Authors carried out also comparative genomics study in the Solanaceae family and inferred phylogenetic studies as well as the domestication history of S. aethiopicum and LD.

Although S. aethiopicum is an orphan species and therefore I do not expect the use of the most advanced technologies for assembly such as PacBio and chromosome scaffolding with HiC, I would have expected at least the anchoring of scaffolds and contigs to pseudomolecules. I think that generating an F2 mapping population for S. aethiopicum is easy to obtain, which could be thus genotyped using any GBS approach authors want.
Response: These are very good suggestions. Unfortunately, we do not have extra budget for this at this moment. Of course, the reference will be further improved and updated once these data are available.

Although a pan genome of the species was also provided, I think that this paper is not suitable for the publication on this journal.
Furthermore, the language needs tightening up and editing for English sense.
Response: The language has been polished.

More detailed comments
Abstract:
it is reported that the pan-genome of S. aethiopicum contains 1,345 genes are missing in the reference genome. I cannot find this in the main text.
Response: The figures in this part have been corrected. Now it has been changed to "A pan-genome of S. aethiopicum with a total of 51,351 protein-coding genes was assembled, of which 24,567 genes are missing in the reference genome sequence." It has also been added in the text.

Background
Line 8-10: I would add some extra reference to this part "It is reported to have medicinal value and its roots and fruits have been used to treat colic, high blood pressure and uterine complications in Africa" or clearly highlighted the information got from FAO. Furthermore, FAO should be added to reference list
Response: The publication of these orphan crops is very few, we could only find this information on the website of FAO (http://www.fao.org/traditional-crops/africangardenegg/en/?amp%3Butm_medium=social%20media&%3Butm_campaign=unfaopinterest), which had already been added to reference list.

Line 24 is (mansfeld.ipk-gatersleben.de). is it a reference for disease resistance? The link send to a database. I would change it with some references from literature.
Response: The full address is http://mansfeld.ipk-gatersleben.de/apex/f?p=185:46:448783208481::NO::module,mf_use,source,akzanz,rehm,akzname,taxid:mf,,botnam,0,,Solanum%20aethiopicum%20Aculeatum%20Group,5898, which is too long and only the website of home page was shown.
Now, we changed it to “Aculeatum is used as ornamentals (Prohens et al., 2012; Plazas et al., 2014) or rootstocks (mansfeld.ipk-gatersleben.de) due to its excellent disease resistance nature (Toppino et al., 2008)”

line 28: please provide at least a reference for this part:"S. aethiopicum is the second most cultivated eggplant, as an "orphan crop"
Response: This statement has been changed to “Although S. aethiopicum is one of the most important cultivated eggplants in Africa, it remains an “orphan crop” because research and breeding investments are substantially lagging behind in comparison with other Solanaceae relatives such as tomato, potato and eggplant.”

Line 40 : the sentence on genome editing sound to me a little bit out of place, as no information on genome editing in scarlet aethiopicum is available. I would point out that genome editing might be used for breeding.
Response: We noticed that there is no report of genome editing in S. aethiopicum so far. This is because very few efforts have been paid to it. However, we believe that these techniques, just like many other advanced techniques, can eventually be applied into this species to speed the progress of breeding. When these platforms are ready, the sequence of genome would be very essential for the identification of genes to be edited, as well as for the design of guide RNAs. This strategy had been proved to be very efficient in a report on Physalis pruinose, another orphan crop also in Solanaceae (Lemmon et al., 2018. Nat. Plants), before which there is not available genome editing example either.

Data description:
I would modify "with a genome size of 1.17 Gb" with "expected genome size". You would get a more precise estimate using flow-cytometry.
Response: Changed.

Furthermore, authors generated more than 242Gb of data, but after cleaning, about 50% of the data (128GB) were used for assembly, which is a quite high percentage. This presumably may explain the number of scaffolds obtained (more than 162k). Did the authors filter for scaffolds' size? Did the authors try to assembly the genome sequence with other tools, like SOAP? Any comments?
Response: Yes, the quality of several of the libraries were poor, therefore we added more libraries to make sure the final clean data is sufficient. We also had tried to assembly the genome using other tools including SOAPdenovo and selected the best assembly for downstream analyses. The assembler automatically filtered out the scaffolds smaller than 100 bp, and all the resulted scaffolds were retained.

Line 33-39. This sentence "Among these annotated TEs, LTR-Rs were extraordinarily abundant and occupied 719 Mbp, accounting for approximately 70% of the genome, followed by LINEs and SINEs (Supplementary Table S4)." is a repetition of what said at the beginning of the paragraph. I will combine the two sentences.
Response: We have deleted this sentence. Thanks.

Powered by Editorial Manager® and ProduXion Manager® from Aries Systems Corporation

	Line 42 Section protein coding. From table S5 gene features are not so similar to other genomes, especially Pepper and Arabidopsis. Furthermore, why pepper has more than 45k genes? The gene number from Kim et al. 2017 is 35,884<br>Response: Arabidopsis is relatively distant to S. aethiopicum. As for the data of Pepper, the data in this table was collected from NCBI (version GCA_000710875.1), which has a total of 45,131 protein-coding genes. The data now has been replaced by Kim's data (Kim et al, 2017).<br><br>Section Amplification of LTR-Rs:<br>* please add references here "The proportion of Ty3/Gypsy and Ty1/Copia LTR-Rs in S. aethiopicum is also comparable to those reported in other Solanaceae genomes."<br>Response: The references were added. The sentence was rephrased to "The proportion of Ty3/Gypsy in S. aethiopicum is also comparable to what is reported in the hot pepper genome (87.7% of Ty3/Gypsy in hot pepper)".<br><br>* Line 19: In this part "they occurred separately in each genome since S. aethiopicum and hot pepper had split about 20 MYA (Figure 1A), and about 4 MYA between S. aethiopicum and tomato (Figure 1A)." authors stated that S. aethiopicum separated from tomato 4 million years ago. This sound strange. S.aethiopicum did not separated from tomato 4 MYA, but only the ancestors of tomato/potato and eggplant/scarlet eggplant, which occurred around 16MYA.<br>Response: Changed to "they occurred separately in each genome since the ancestor of S. aethiopicum had diverged from that of hot pepper and tomato about 20 MYA and 4 MYA, respectively".<br><br>Furthermore, the second LTR burst occurred 1.25MYA was also shared by eggplant?<br>Response: No, but eggplant has a burst more recently, about 0.5 MYA (Figure 2A)<br><br>Polymorphisms in different S. aethiopicum groups section:<br>Concerning the ADMIXTURE analysis and results, I wonder why authors did not define accessions belonging for less than, let's say 70%, to a group as admixed.<br>Response: The accessions were clustered using ADMIXTURE following the methods previously described in (Mathieson et al. 2017; Olalde et al., 2017; Mittnik et al., 2017), and we did not see an example in which accessions were grouped as suggested.<br><br>Artificially selected genes in S. aethiopicum<br>I would have expected, at least for the 12 genes in common between Gilo and Shum (and maybe for the 36 selected genes in Shum), some more information. What genes are they?<br>Response: The functional descriptions have been listed in a new table, Supplementary Table 18.<br><br>Go enrichments are nice but sometimes it would be better to provide some more details, especially if the number of genes involved are limited.<br>Response: Added<br><br>Pan-genome section<br>* Why did the authors get less contigs for Anguivi? The sequencing performance are quite good for the 5 accessions of this species.<br>Response: The contigs were assembled separately for each individual, Anguivi had fewer contigs only because the number of Anguivi accessions used in this study is small (5 for Anguivi, and 24 for Gilo and 36 for Shum)<br><br>* I am quite confused on the metrics (Supplementary table S20). In the text, it is reported that 41,626, 22,942 and 17,726 protein-coding genes for "Shum", "Gilo" and "S. anguivi, respectively were predicted, among which accessory gene sets of 29,389, 23,726 and 12,829 for "Shum", "Gilo" and "S. anguivi", respectively were found. These numbers are not the same in S20 table, presumably two columns were switched.<br>Furthermore in the table S22 for Gilo, a total of 33,194 gene are reported, while in the text the number is 22,942. Accessory genes in the text for Gilo are less than the ones predicted (as reported in the text).<br><br>* Table S20, I will add the unit of measurement for length

Powered by Editorial Manager® and ProduXion Manager® from Aries Systems Corporation

	* I cannot find Supplementary Table S21 and S22<br>Response: The two columns were switched in Supplementary table 21 (previous supplementary table 20) and we forgot to add supplementary table 22 and 23. We have corrected the errors and add the unit of measurement for length.<br><br>Methods<br>Gene family analysis: References for the 5 proteomes used are missing, as well as the version used<br><br>Response: The references and version of the data have been added.<br><br>NLR genes: it is not clear to me how the NLR genes were identified. In methods is reported that specific NB-ARC HMM model was constructed, but in the text it is reported that NBS-LRR genes were identified.<br>How did the authors performed the identification of other Motifs (TIR, CC and LRR)?<br>Response: The "NBS-LRR gene" in the text was supposed to be "NB-containing genes". We counted the number of "NB-containing genes" because, even without LRR motif, NB-containing genes can also function in plant immunity (Nandety et al., 2013).<br><br>SNP calling: which parameters did the authors use for SNP identification? Besides MAF and GENO parameters, I would also have considered sequencing depth as a key parameter for the final SNPs set.<br>Response: Yes, sequencing depth is critical. Actually, the depth had been considered, and it is not a problem because the sequencing depth for each accession is averagely higher than 60 X in our work.<br><br>Population analyses. I would add bootstrap values to the figure 5A<br>Response: As the branches in the figure are too short, we added the phylogenetic tree with bootstrap in supplementary figure 4.<br><br>Furthermore, is the reference for Itools (80) correct?<br>Response: The software itools used in our research has been changed to a new name, called ReSeqTools. We have changed it to the correct software name in our article.
**Additional Information:**	
**Question**	**Response**
Are you submitting this manuscript to a special series or article collection?	No
**Experimental design and statistics**<br><br>Full details of the experimental design and statistical methods used should be given in the Methods section, as detailed in our Minimum Standards Reporting Checklist. Information essential to interpreting the data presented should be made available in the figure legends.<br><br>Have you included all the information requested in your manuscript?	Yes
**Resources**<br><br>A description of all resources used,	Yes

Powered by Editorial Manager® and ProduXion Manager® from Aries Systems Corporation

including antibodies, cell lines, animals and software tools, with enough information to allow them to be uniquely identified, should be included in the Methods section. Authors are strongly encouraged to cite Research Resource Identifiers (RRIDs) for antibodies, model organisms and tools, where possible.<br><br>Have you included the information requested as detailed in our Minimum Standards Reporting Checklist?	
**Availability of data and materials**<br><br>All datasets and code on which the conclusions of the paper rely must be either included in your submission or deposited in publicly available repositories (where available and ethically appropriate), referencing such data using a unique identifier in the references and in the "Availability of Data and Materials" section of your manuscript.<br><br>Have you have met the above requirement as detailed in our Minimum Standards Reporting Checklist?	Yes

Manuscript

Click here to access/download;Manuscript;Draft genome sequence of the S.aethiopicum_edit_BoSong.docx

Click here to view linked References

Draft genome sequence of *Solanum aethiopicum* provides insights into disease resistance, drought tolerance and the evolution of the genome

Bo Song[1,2,3, ‡](0000-0003-1102-2814), Yue Song[1,3,4, ‡](0000-0002-2974-6442), Yuan Fu[1,2, ‡](0000-0002-3867-1219), Elizabeth Balyejusa Kizito[5](0000-0003-2558-8309), Sandra Ndagire Kamenya[5,6], Pamela Nahamya Kabod[5], Huan Liu[1,2,3](0000-0003-3909-0931), Samuel Muthemba[7](0000-0003-3311-8489), Robert Kariba[7](0000-0003-0242-4547), Joyce Njuguna[6], Solomon Maina[6](0000-0003-0205-8567), Francesca Stomeo[6,8](0000-0003-1776-6128), Appolinaire Djikeng[6,9], Prasad S. Hendre[7](0000-0003-1691-183X), Xiaoli Chen[1,2](0000-0002-4878-1905),Wenbin Chen[1,2], Xiuli Li[1,2], Wenjing Sun[1,2], Sibo Wang[1,3], Shifeng Cheng[1,2], Alice Muchugi[7], Ramni Jamnadass[7](0000-0003-2416-8361), Howard-Yana Shapiro[7,10,11], Allen Van Deynze[10](0000-0002-2093-0577), Huanming Yang[1,2], Jian Wang[1,2], Xun Xu[1,2,3](0000-0002-5338-5173), Damaris Achieng Odeny[12,*](0000-0002-3629-3752) and Xin Liu[1,2,3,*](0000-0003-3256-2940)

[1] BGI-Shenzhen, Beishan Industrial Zone, Yantian District, Shenzhen 518083, China; [2] China National GeneBank, BGI-Shenzhen, Jinsha Road, Shenzhen 518120, China; [3] State Key Laboratory of Agricultural Genomics, BGI-Shenzhen, Shenzhen 518083, China; [4] BGI-Qingdao, BGI-Shenzhen, Qingdao 266555, China; [5] Uganda Christian University, Bishop Tucker Road, Box 4, Mukono, Uganda; [6] Biosciences Eastern and Central Africa (BecA) – International Livestock Research Institute (ILRI) Hub, P.O. Box 30709 Nairobi, 00100 Kenya; [7] African Orphan Crops Consortium, World Agroforestry Centre (ICRAF), Nairobi,

Kenya; [8] Present address: European Molecular Biology Laboratory (EMBL), Heidelberg, Germany; [9] Present address: Centre for Tropical Livestock Genetics and Health (CTLGH), University of Edinburgh, Edinburgh EH25 9RG, UK; [10] University of California, 1 Shields Ave, Davis, CA, USA; [11] Mars, Incorporated, 6885 Elm Street, McLean, Virginia, 22101, USA; [12] International Crops Research Institute for the Semi-Arid Tropics (ICRISAT) – Eastern and Southern Africa, P.O. Box 39063, Nairobi 00623 Kenya

‡ Equal contribution

*Correspondence address. Xin Liu. BGI-Shenzhen, Beishan Industrial Zone, Yantian District, Shenzhen 518083, China. Tel: +86-180-2546-0332; E-mail: liuxin@genomics.cn

Damaris Achieng Odeny. P.O Box 39063 – 00623, Nairobi, Kenya Tel: +254 20 7224559; E-mail: D.Odeny@cigar.org

Abstract

The African eggplant (*Solanum aethiopicum*) is a nutritious traditional vegetable used in many African countries, including Uganda and Nigeria. It is thought to have been domesticated in Africa from its wild relative, *S. anguivi*. *S. aethiopicum* has been routinely used as a source of disease resistance genes for several Solanaceae crops, including *S. melongena*. A lack of genomic resources has meant that breeding of *S. aethiopicum* has lagged behind other vegetable crops. We assembled a 1.02 Gb draft genome of *S. aethiopicum*, which contained

predominantly repetitive sequences (76.2%). We annotated 37,681 gene models, including 34,906 protein-coding genes. Expansion of disease resistance genes was observed via two rounds of amplification of long terminal repeat retrotransposons, which may have occurred around 1.25 and 3.5 million years ago, respectively. By re-sequencing 65 *S. aethiopicum* and *S. anguivi* genotypes, 14,995,740 single nucleotide polymorphisms (SNPs) were identified, of which 41,046 were closely linked to disease resistance genes. Analysis of domestication and demographic history revealed active selection for genes involved in drought tolerance in both 'Gilo' and 'Shum' groups. A pan-genome of *S. aethiopicum* was assembled, containing 51,351 protein-coding genes; 7,069 of these genes were missing from the reference genome. The genome sequence of *S. aethiopicum* enhances our understanding of its biotic and abiotic resistance. The single nucleotide polymorphisms identified are immediately available for use by breeders. The information provided here will accelerate selection and breeding of the African eggplant, as well as other crops within the Solanaceae family.

Keywords: *Solanum aethiopicum*; African eggplant; *Solanum anguivi*; LTR-Rs; biotic stress; drought tolerance.

Background

The African eggplant, *Solanum aethiopicum* (NCBI:txid205524), is an indigenous non-tuberiferous Solanaceae crop that is mainly grown in tropical Africa [1], especially in

Central and West Africa. *S. aethiopicum* is hypervariable [2, 3] and is generally classified into four groups: Gilo, Shum, Kumba and Aculeatum. Gilo is the most important group and has edible fruits, while Shum has small and bitter fruits. Kumba is used as a leafy vegetable, while Aculeatum is used as an ornamental [3, 4] or as rootstock because of its excellent disease resistance [5]. The African eggplant is reported to have anti-inflammatory activity [6] and its roots and fruits have been used to treat colic, high blood pressure and uterine complications in Africa [6].

Although *S. aethiopicum* is one of the most important cultivated eggplants in Africa [7, 8], it remains an 'orphan crop' because research and breeding investments are lagging behind other Solanaceae relatives, such as *S. lycopersicum* (tomato), *S. tuberosum* (potato) and *S. melongena* (edible eggplant). Consequently, there have been few robust genomic resources, such as a well-annotated reference genome. Genomics-assisted breeding is an effective approach that would facilitate the breeding of orphan crops such as the African eggplant. Previous attempts to develop molecular markers for *S. aethiopicum*, using the *S. melongena* genome as a reference, have been unsuccessful because of compromised accuracy [9]. An alternative approach that uses genome editing has been successfully deployed in other Solanaceae crops, including *Physalis pruinose* [11, 12], but cannot be implemented in *S. aethiopicum* because of its lack of well-annotated reference genome and gene sequences.

The African eggplant serves as a gene reservoir for other economically important crops within the Solanaceae family. Thanks to its cross-compatibility with *S. melongena* [4,

10] and its outstanding resistance to various pathogens, including *Fusarium*, *Ralstonia* and *Verticillium* [5, 11–13], *S. aethiopicum* has been used to develop rootstocks [13] or improve the disease resistance of *S. melongena* [14]. As the genomic basis of resistance in *S. aethiopicum* is poorly understood, it can be time-consuming to use it as a donor in such interspecific crosses. Mapping resistance genes and then developing markers associated with these genes might resolve this challenge. The development and expansion of resistance genes is usually accompanied by the amplification of long terminal repeat retrotransposons (LTR-Rs). A typical example is shown in the Solanaceous hot pepper (*Capsicum annuum*), in which a burst of LTR-Rs substantially mediated the retrotransposition of nucleotide-binding, leucine rich repeat-related (NLR) genes, leading to the expansion of resistance genes [15]. LTR-Rs are abundant in plant genomes, including Solanaceae crops such as *Nicotiana sylvestris* (~38.16%) [16], pepper (more than 70.0%) [17], potato (62.2%) [18], tomato (50.3%) [19] and Petunia (more than 60%) [20]. The role of LTR-Rs in the *S. aethiopicum* genome remains unknown and whether the resistance seen in *S. aethiopicum* is a result of LTR-R amplification remains to be investigated. The generation of a reference genome for *S. aethiopicum*, as well as for other orphan crops, is urgently needed to advance their research and breeding.

Here, we report a draft whole genome assembly and annotation for *S. aethiopicum*. We found two amplifications of LTR-Rs that occurred around 1.25 and 3.5 million years ago (MYA), resulting in the expansion of resistance genes. We also resequenced two *S. aethiopicum* groups, ‘Gilo’ and ‘Shum’, at a high depth (~60 X) and identified

14,995,740 single nucleotide polymorphisms (SNPs), 41,046 of which are closely linked to resistance genes. Subsequently, we generated a pan-genome of *S. aethopicum*. The genomic data provided in this study will greatly advance research and breeding activities of the African eggplant.

Data Description

We sequenced the genome of *S. aethiopicum* using a whole-genome shotgun (WGS) approach. A total of 242.61 Gb raw reads were generated by sequencing the libraries with insert sizes of 250 and 500 bp, and mate-pair libraries with sizes ranging between 2,000 and 20,000 bp, on an Illumina Hiseq 2000 platform. The filtered reads used for downstream analysis are shown in Supplementary Table 1. *k*-mer (k = 17) analysis [21] revealed the *S. aethiopicum* genome to be diploid and homozygous, with an estimated genome size of 1.17 Gb (Supplementary Figure 1). 'Clean reads' amounting to 127.83 Gb (~ 109 X) were used to assemble the genome using Platanus [22] (see Methods). A final assembly of 1.02 Gb in size was obtained, containing 162,187 scaffolds with N50 contig and scaffold values of 25.2 Kbp and 516.15 Kbp (Table 1 and Supplementary Table 2), respectively. Our results reveal that the *S. aethiopicum* genome is larger than that of other *Solanum* genomes, including tomato (0.76 Gb) and potato (0.73 Gb) [18, 19], but it has a comparable GC ratio (33.12%) (Supplementary Table 3).

Repetitive elements, predominantly transposable elements (TE) (Supplementary Table 4), occupied 790 Mbp (76.2%) of the sequenced genome. Most annotated TEs were

retrotransposon elements, including long terminal repeats (LTRs), short interspersed elements (SINEs) and long interspersed elements (LINEs). Together these retrotransposons made up 75.42% of the assembly. DNA transposons accounting for 2.87% of the genome were also annotated (Supplementary Table 4).

Protein-coding gene models were predicted by a combination of homologous search and *ab initio* prediction. The resulting models were pooled to generate a final set of 34,906 protein-coding genes. Predicted gene models were, on average, 3,038 bp in length, with an average of 3.15 introns. The average length of coding sequences, exons and introns was 1,104 bp, 265 bp and 613 bp, respectively (Table 1, Supplementary Table 5, Supplementary Figure 2). As expected, these gene features were similar to those of other released genomes, including *Arabidopsis thaliana* [23] and other Solanaceae crops including *S. lycopersicum, S. tuberosum, C. annuum* and *N. sylvestris* [16, 18, 19, 24] (Supplementary Table 5). We further assessed the annotation completeness of this assembly by searching for 1,440 core embryophyta genes (CEGs) with Benchmarking Universal Single-Copy Orthologs (BUSCO, version 3.0) [25]. We found 80.4% CEGs in this assembly, with 77.8% being single copies and 2.6% being duplicates (Supplementary Table 6). We also annotated the non-coding genes by homologous search, leading to the identification of 128 microRNA, 960 tRNA, 1,185 rRNA and 503 snRNA genes (Supplementary Table 7).

We annotated 31,863 (91.28%) proteins for their homologous function in several databases. Homologs of 31,099 (89.09%), 26,319 (75.4%) and 20,932 (59.97%)

proteins were found in TrEMBL, InterPro and SwissProt databases, respectively (Supplementary Table 8). The remaining 3,043 (8.72%) genes encoded putative proteins with unknown functions.

Analyses

Genome evolution and phylogenetic analysis

By comparing with four other sequenced Solanaceae genomes (*S. melongena*, *S. lycopersicum*, *S. tuberosum* and *C. annuum*), 25,751 of the *S. aethiopicum* genes were clustered into 19,310 families using OrthoMCL (version 2.0) [26], with an average of 1.33 genes each. Single-copy genes shared by these five genomes were concatenated as a super gene representing each genome and were used to build a phylogenetic tree (Figure 1A). The split time between *S. aethiopicum* and *S. melongena* was estimated to be ~2.6 MYA. McScanX [27] identified 182 syntenic blocks. We detected evidence of whole genome duplication (WGD) events in this genome by calculating the pairwise synonymous mutation rates and the rate of four-fold degenerative third-codon transversion (4DTV) of 1,686 paralogous genes in these blocks. The 4DTV distribution plot displayed two peaks, at around 0.25 and 1, indicating two WGDs (Figure 1B). The first one (peak at 1) represents the ancient WGD event shared by asterids and rosids [28], while the second WGD event is shared by Solanaceae plants. This suggests that its occurrence predates the split of Solanaceae.

Evolution of gene families

OrthoMCL [26] clustering of genes from *S. aethiopicum*, *S. melongena*, *S. lycopersicum*, *S. tuberosum* and *C. annuum* identified 25,751 gene families. Among these, 465 gene families were unique to *S. aethiopicum* and 10,166 were common (Supplementary Table 9, Figure 1C). As expected, the number of shared gene families decreased as a function of evolutionary distance between *S. aethiopicum* and the selected species (Supplementary Table 10). For example, *S. aethiopicum* shared 15,723 gene families with *S. melongena*, compared with only 13,461 genes shared with *C. annuum*. To further investigate the evolution of gene families, we identified expanded and contracted gene families. Compared with *S. melongena*, 437 gene families were expanded; most expanded gene families were found to be involved in biological processes related to drought or salinity tolerance or disease resistance, including defense response (GO:0006952), response to oxidative stress (GO:0006979), glutamate biosynthetic processes (GO:0006537) and response to metal ions (GO:0010038) (Supplementary Table 11). No gene families were contracted when comparing with *S. melongena*.

Amplification of LTR-Rs

LTR-Rs comprised ~70% of the genome and accounted for 89.31% of the total TEs in *S. aethiopicum* (Supplementary Table 4). Consistent with previous studies of LTR-Rs, most LTR-Rs were classified as being in *Ty3/Gypsy* (82.36% of total LTR-Rs) and *Ty1/Copia* (14.90% of total LTR-Rs) subfamilies. The proportion of *Ty3/Gypsy* in *S. aethiopicum* is comparable to that reported in the hot pepper genome (87.7% of *Ty3/Gypsy*) [24]. To investigate the roles of LTR-Rs in the evolution of *S. aethiopicum*, we detected 36,599 full-length LTR-Rs using LTRharvest [29] with the parameters "-maxlenltr 2000, -similar 75" and LTRdigest software [30]. We further analyzed their evolution, activity and potential biological functions.

The age of each LTR-R was inferred by comparing the divergence between the 5′ and 3′ LTR-R, using a substitution rate of 1.3e-8 $year^{-1}site^{-1}$ [31]. Two amplifications of LTR-Rs were found in *S. aethiopicum*, while only one was detected in tomato and hot pepper (Figure 2A). The early amplification occurred at around 3.5 MYA, coincident with the LTR-R burst found in *C. annuum* [15] (Figure 2A). The second amplification was at 1.25 MYA, coinciding with the LTR-R burst in the tomato genome [19] (Figure 2A). Although the time of LTR-Rs amplification is vertically coincident between different species, they occurred separately in each genome since the ancestor of *S. aethiopicum* diverged from that of hot pepper and tomato about 20 MYA and 4 MYA, respectively (Figure 1A). These results imply that environmental stimulators shared between these species during their evolution could have triggered the amplifications observed. We also estimated the amplification time of *Ty3/Gypsy* and *Ty1/Copia* LTR-Rs and found two peaks at around 1.25 MYA and 3.5 MYA for Gypsy LTR-Rs (Figure

2B), but only one peak (around 1.25 MYA) for *Ty1/Copia* LTR-Rs (Figure 2C). Compared with the amplification time of *Ty3/Gypsy* and *Ty1/Copia* LTR-Rs in different species, we observed that the insertion time of *Ty1/Copia* LTR-RTs in *S. aethiopicum* and tomato were earlier than that of *S. melongena* and hot pepper. On the contrary, the insertion time of *Ty3/Gypsy* LTR-RTs (around 3.5 MYA) in *S. aethiopicum* was consistent with the insertion time of hot pepper (Figure 2B, 2C).

To investigate the activities of these LTR-Rs, we measured their expression levels by using RNA-seq data from different tissues (see Methods). Younger LTR-Rs were expressed in higher levels than those of older LTR-Rs. We detected two peaks of LTR-R activity, at positions corresponding to the two rounds of LTR-R insertions (Figure 2D–G). The slight shift of the former peaks indicates that the activities degenerated slower than the LTR-R sequences (Figure 2D–G). The LTR-R activities varied across these tissues. The degeneration of LTR-R activities was slower in fruits and roots that those in flowers and leaves (Figure 2D). This pattern was also confirmed by the varied activity of each LTR-R across these tissues (Figure 2D), implying that these LTR-Rs have different roles in development.

Increased resistance is facilitated by LTR-Rs amplification

We identified 1,156 LTR-R captured genes and 491 LTR-R disrupted genes. The insertion time of LTR-R captured and LTR-R disrupted genes both ranged between 1.5 and 3.5 MYA (Figure 3A), showing a pattern similar to the insertions of whole LTR-Rs

(Figure 2A). These results suggest that LTR-R-mediated gene disruption and capture occurred simultaneously. We further classified the LTR-R captured genes into Gene Ontology (GO) categories and performed GO enrichment analysis. GO terms related to disease resistance including ‘defense response to fungus (GO:0006952)’, ‘chitin catabolic process (GO:0006032)’, ‘chitinase activity (GO:0004568)’, ‘chitin binding (GO:0008061)’, ‘cell wall macromolecule catabolic process (GO:0016998)’ and ‘defense response to bacterium (GO:0042742)’ were overrepresented in the LTR-R captured genes (Figure 3B, Supplementary Table 12), suggesting that they may be involved in enhancing disease resistance.

We also analyzed the expression of genes captured by LTR-Rs. It was intriguing to find that most of these genes were active in only one tissue (Supplementary Figure 3). Among these genes, 159 (13.75%), 105 (9.08%), 106 (9.16%) and 129 (11.15%) were specifically and highly expressed in root, leaf, flower and fruit, respectively. The genes captured by LTR-Rs that were specifically active in leaf tissues were significantly enriched in functions relating to disease resistance (Supplementary Table 13). The biological processes and molecular activities related to disease resistance mentioned above were overrepresented in these genes (Figure 3C). The high expression level of resistance genes in leaves would arm the plant with stronger resistance to pathogens. On the contrary, these GO terms were not enriched in the genes that were specifically and highly expressed in leaves. Instead, as expected, ‘photosynthesis’ and ‘photosystem I’ were significantly overrepresented (Supplementary Table 14). The discrepancy

between these two gene sets highlights the contribution to resistance of LTR-R captured genes.

Proteins containing nucleotide-binding, leucine-rich repeat domains (NB-LRRs) are major components that are responsible for defense against various phytopathogens [32]. The NB-LRR family is highly expanded in plants, with numbers ranging from less than 100 to more than 1,000 [33, 34]. As NB-LRR genes are often co-localized with LTR-Rs [35], we inspected their genomic locations in the *S. aethiopicum* genome. Because proteins containing the nucleotide-binding (NB) site can also confer disease resistance, we searched for all the NB-containing genes in the genome. As a result, we identified 447 NB-containing genes in the genome, among which 62 (13.8%) NB-containing genes co-localized with LTR-Rs were identified as LTR-R captured genes. The phylogenetic tree shows a substantial expansion of NB-containing genes after the amplification of LTRs in *S. aethiopicum* (Figure 3D). A similar expansion was also observed in *S. melongena*. However, the number was significantly fewer than in *S. aethiopicum*, probably because of the limited number of LTR-Rs in the *S. melongena* genome (Supplementary Table 15).

Polymorphisms in different *S. aethiopicum* groups

We resequenced 60 *S. aethiopicum* genotypes in two major groups, ‘Gilo’ and ‘Shum’, and five accessions of *S. anguivi*, the progenitor of *S. aethiopicum* [36]. We generated ~60 Gb raw data (60 X) (Supplementary Table 20) and identified 18,614,838 SNPs and

1,999,241 indels, with an average of 3,530,488 SNPs for each accession (Supplementary Table 16). On average, there were 18,090 SNPs and 1,943 indels per megabase. Among them, 426,401 (2.07%), 821,101 (3.98%) and 19,374,353 (93.99%) were located in exons, introns and intergenic regions, respectively (Table 2). There were 267,710 SNPs that resulted in amino acid sequence changes by introducing new start codons, premature stop codons, or nonsynonymous substitutions (Table 2). We also identified 1,999,241 indels and 1,255,302 structural variations (SVs). Of the detected indels, 178,260 (8.90%) were located in genic regions, among which 2,977 (0.13%) caused frameshift changes and, therefore, resulted in amino acid sequence changes that may have led to gene malfunctions. Furthermore, 106,377 SVs were identified in genic regions, including 53,736 (50,51%) deletions, 34,368 (32,31%) insertions and 8,872 (8.34%) duplications.

On counting the SNPs and indels in each group, we found 12,777,811, 15,165,053 and 8,557,818 SNPs in 'Gilo', 'Shum' and '*S. anguivi*', respectively, accounting for 68.64%, 81.47% and 45.97% of the total SNPs, respectively. There were, 2,019,539 (10.85%), 4,747,418 (25.50%) and 587,885 (3.16%) SNPs unique to 'Gilo', 'Shum' and '*S. anguivi*', respectively (Figure 4A). Most (93.13%) SNPs in '*S. anguivi*' were shared with either 'Gilo' or 'Shum' (Figure 4A), which is in line with the fact that '*S. anguivi*' is the ancestor [36]. Similarly, 92.62% of the indels identified in '*S. anguivi*' were also shared with 'Gilo' or 'Shum' (Figure 4B).

Nucleotide diversity (π) of all the genotypes was determined to be 3.58×10^{-3} for whole

genomes, 2.06×10^{-3} for genic regions and 3.75×10^{-3} for intergenic regions. Nucleotide diversity for each genotype revealed lower diversity for 'Gilo' (*S. anguivi*: 3.16×10^{-3}, Shum: 3.65×10^{-3} and Gilo: 2.55×10^{-3}, respectively). Linkage disequilibrium (LD) estimation using Haploview (version 4.2) [37] revealed that r^2 reached the half maximum value at ~150 kb (Figure 4C), which is smaller than in other Solanaceae crops; for example, tomato (2,000 kb) [38]. Since *S. aethiopicum* has been routinely used to improve disease resistance in eggplant and other Solanaceaee crops [14], we further identified SNPs that were strongly associated with resistance genes by selecting those lying within 150 kb of resistance genes. A total of 5,562 SNPs were finally selected (205 genes), which could be used in the selection of Solanaceae plants with disease resistance (Supplementary Table 16).

Population structure and demography of *S. aethiopicum*

To investigate the evolution and population demography of *S. aethiopicum*, we first built a maximum-likelihood (Figure 5A, Supplementary Figure 4) phylogenetic tree using the full set of SNPs. We observed population structure in the genome-wide diversity. As anticipated, the accessions from 'Gilo' and 'Shum' were clearly separated in the tree, with only one exception in each group, probably caused by labelling errors. On the other hand, accessions of '*S. anguivi*', the known ancestor of *S. aethiopicum*, did not cluster separately, but grouped with either 'Gilo' or 'Shum'. This structure was also supported by principal component analysis (PCA), which clearly separated these

accessions into two clusters (Figure 5B, Supplementary Figure 5).

The domestication history of *S. aethiopicum* was inferred by constructing a multilevel population structure using ADMIXTURE [39]. This enabled us to estimate the maximum likelihood ancestry (Figure 5A). The parameter K, representing the number of subgroups to be divided, was set from 2–9, and the cross-validation (CV) error was calculated individually. The CV error converged to 0.4375 when K = 6, suggesting the division of the resequenced accessions into six subgroups: I–VI (Figure 5A). The structure changes with increasing K-value from 2 to 6, showing a timelapse domestication history of *S. aethiopicum* that was first split into two groups, 'Gilo' and 'Shum'. The former was subsequently divided into subgroups I and II. Two groups emerged in 'Shum' when K = 3, each of which was then divided into two subgroups when K = 6. In summary, 'Gilo' was divided into two subgroups (I and II) and 'Shum' was divided into four subgroups (III–VI).

The demographic history of *S. aethiopicum* was inferred using the pairwise sequential Markovian coalescent model (PSMC) [40]. By doing this, we inferred changes in the effective population sizes of *S. aethiopicum* (Figure 5C). Our data revealed distinct demographic trends from 10,000 to 100 years ago, in which a bottleneck was shown around 4,000–5,000 years ago, followed by an immediate expansion of population size. The great population expansion might be associated with the early domestication of *S. aethiopicum* in Africa, since it coincides with human population growth in western Africa, also occurring 4,000–5,000 years ago [41].

Artificially selected genes in *S. aethiopicum*

We used *ROD* and *Fst* measures to detect artificially selected regions along the genome. Briefly, *ROD* and *Fst* were calculated in a sliding non-overlap 10-kb window. Regions with *ROD* > 0.75 and *Fst* > 0.15 were identified as candidate regions under selection. As a result, genomic regions of 3,238 and 1,062 windows were found to be under selection during the domestication of ‘Gilo’ and ‘Shum’, respectively (Supplementary Table 17). Among them, 161 windows were common between these two groups, while 3,077 and 901 windows were unique to ‘Gilo’ and ‘Shum’, respectively. Genes located within these regions were identified as selected genes. Thirty-six and 1,406 selected genes were identified in ‘Shum’ and ‘Gilo’, respectively, and 12 of these genes were selected in both. Ten of the 12 genes were annotated in the SwissProt database with known functions and included many genes known to be involved in tolerance to unfavorable environmental stresses, such as autophagy-related gene 18f (*ATG18f*), ATP-binding cassette transporter B (*ABCB18*), lysine--tRNA ligase (*LYSRS*), acyl-coenzyme A oxidase 4 (*ACX4*), inositol hexakisphosphate and diphosphoinositol-pentakisphosphate kinase (*VIP2*) (Supplementary Table 18). For example, ATG18 is reported to be involved in defense response to powdery mildew fungus through autophagy in *Arabidopsis* [42]; it is also involved in response to nutrition starvation by serving as an accessory component to ATG1/13 kinase complex [43]. ABCB is reported to be associated with lipid transport and confers tolerance to heavy metal ions, such as

aluminium [44], cadmium and lead [45]. The expression of *LYSRS* has been shown to be specifically induced in tomato root during the unusual accumulation of metal ions [46]. *VIP2* is reported to be critical in myo-inositol phosphates (InsPs) signalling pathways, and is known to be involved in responses to drought and salt stresses [47]. Furthermore, two genes encoding pentatricopeptide repeat-containing protein were also found among these genes, suggesting that RNA editing may have played a crucial role in the domestication of *S. aethiopicum* [48]. GO enrichment analysis showed that genes selected in both the ‘Gilo’ and ‘Shum’ groups were enriched in ‘transport’ (Supplementary Table 19). GO terms for ‘response to auxin’, ‘response to hormone’, ‘response to salt stress’ and ‘response to water’ were also overrepresented in genes selected either in ‘Gilo’ or ‘Shum’ only. This result could explain the enhanced tolerance to drought and salinity in *S. aethiopicum*.

We also focused on the diversity of genes co-localized with LTR-Rs. A total of 24,682 SNPs were located within these co-localized genes, corresponding to 0.133% of the total number of SNPs (18,614,838). This is substantially fewer than would be expected if SNPs were evenly distributed across all genes, particularly because the LTR-R co-localized genes comprise 3.31% of the total gene set. The repellant of SNPs in these genes suggests purifying selection, which was also supported by the large amount (9,728; 39.41%) of rare SNPs (minor allele frequency <5%) found among the co-localized genes. We also observed that nonsynonymous SNPs (9,544) were much more abundant than synonymous ones (5,310) among the co-localized genes. These variations led to amino acid changes in the encoded proteins, which may have

contributed to the diversification of resistance genes.

Pan- and core-genome of *S. aethiopicum*

Gene content varies across different accessions. A single reference assembly is insufficient to include all *S. aethiopicum* genes. Therefore, we assembled contigs for individual accessions using pair-end reads, with coverages ranging from 30–60 X (Supplementary Table 20).

We assembled the genomes individually using SOAPdenovo2 [49] and filtered out contigs smaller than 2 kb. As a result, 753,084 contigs were retained, among which 432,785 were from 'Shum', 260,119 were 'Gilo' and 60,180 were from '*S. anguivi*'. These contigs were further pooled separately and cleaned by removing duplicates using CD-HIT [50]. This led to the retention of 97,429, 76,638 and 36,915 contigs for 'Shum', 'Gilo' and '*S. anguivi*', respectively. The annotation of these contigs resulted in 41,626, 33,194 and 17,662 protein-coding genes, among which we identified accessory gene sets of 29,389, 23,726 and 12,829 for 'Shum', 'Gilo' and '*S. anguivi*', respectively, by comparing against the reference genome sequence. We generated a pan-genome of *S. aethiopicum* (including 'Shum', 'Gilo' and '*S. anguivi*' groups) of 51,351 genes (Supplementary Table 21). These genes were further clustered together with those annotated in the reference using CD-HIT. Overall, we identified 7,069 genes unique to the pan-genome gene set, suggesting that they had been missed from the reference. The average length of accessory genes was 1.62 kb with 2.22 introns. This is comparable to

gene models in the reference genome, providing further evidence of accurate annotation. We further assigned their putative functions by querying against protein databases. A total of 48,572 (94.59%) genes were fully annotated and functional descriptions (Supplementary Table 22) provided. Among the identified gene models, 10,409 (20.27%) were common to these three groups and were thus defined as ‘core’ genes. As expected, they were mainly composed of housekeeping genes (Supplementary Table 23). However, it is important to note that the number of core genes may have been underestimated because ‘*S. anguivi*’ was under-represented, while the other two *S. aethiopicum* groups, Kumba and Aculeatum, were not included in the current study.

Discussion

Solanum aethiopicum is cross-compatible with *S. melongena* and is routinely used as a donor of disease resistance genes to its close relative [14]. Genomic analysis of *S. aethiopicum* revealed higher LTR-mediated expansion of resistance gene families than its other close relatives, including tomato, potato, eggplant and hot pepper. LTR amplification is one of the major forces driving genome evolution. It shapes the genome by capturing, interrupting or flanking genes [51]. The consequences of LTR insertions depend on the genomic position of insertion. For example, inserting into protein-coding sequences results in pseudogenisation. LTR-Rs adjacent to protein-coding genes can downregulate or silence the expression of flanking genes by extending methylation regions or by producing antisense transcripts [52–55]. LTR-Rs also mediate gene

retroposition, capturing genes back into the genome [51]. In the current study, LTRs preferentially captured genes related to disease resistance, resulting in the over-representation of GO terms related to disease resistance in the LTR-captured genes. Enrichment of the GO terms ‘chitin binding (GO:0008061)’ and ‘chitinase activity (GO:0006032)’ (Figure 3B, Supplementary Table 12) implies that these genes may have been selected to resist infection by fungal pathogens, such as *Fusarium oxysporum* [56]. On the contrary, no GO term enrichment was seen in genes that were disrupted by LTR-Rs. This suggests that gene disruption by LTR-Rs may be a random event in terms of gene function. The age distribution of LTR-R captured genes coincidently fit with that of the LTR-R disrupted genes, suggesting that these two events may have occurred simultaneously (Figure 3A). It is not clear why genes related to disease resistance were favoured by LTR-Rs, but one explanation is that the disease resistance genes may have been more active than other genes at the time of LTR retrotransposition. The expression pattern of LTR-R captured genes also varied between tissues. Those related to resistance were specifically active in the leaf, while those engaged in the transport of cations, nitrogen and cell proliferation were active in flowers. This outcome suggests low abundance of transcripts for disease resistance genes, resulting in a relatively low chance to adequately capture the genes in flowers under normal conditions. Another possible scenario is that LTR retrotransposition occurred under stress conditions, which resulted in the simultaneous induction of the expression of resistance genes in gametes and the activity of LTR retrotransposition. Such possible stresses might be extreme environmental conditions or pathogen infection. A ‘reinforcement model’ has been

proposed to explain the simultaneous accumulation of stress responsive genes and the activity of retrotransposons in genomes under environmental stress [57, 58].

There are four major groups of *S. aethiopicum*: ‘Gilo’, ‘Shum’, ‘Kumba’ and ‘Aculeatum’. We resequenced accessions from the ‘Gilo’ and ‘Shum’ groups, which are widely consumed as vegetables. The accessions resequenced in this study were clustered into six subgroups (two for ‘Shum’ and four for ‘Gilo’). By scanning for regions with lower genomic diversity, we identified regions and several genes involved in responses to salt, water and drought tolerance that were under selection during the domestication of *S. aethiopicum*. Furthermore, purification selection was also found among disease resistance genes.

In the current study, resequencing *S. aethiopicum* and *S. anguivi* genomes at a high depth (30–60 X) (Supplementary Table 20) enabled us to assemble draft genomes for these individuals. Despite resequencing only a few genotypes from the two groups, we intend to supplement the reference gene set with accessory genes by pooling the resequenced contigs for gene prediction and annotation. This ‘pan-genome’ is expected to provide a more comprehensive understanding of *S. aethiopicum* in the future.

We report a reference genome for African eggplant, which will provide a basic data resource for further genomic research and breeding activities for *S. aethiopicum*. The gene sequences annotated in the genome will be essential for developing genome editing vectors to create mutants to further understand the functions of genes within the genome and develop superior genotypes. Molecular markers developed using the

genome sequences will also enable more efficient and precise selection of superior accessions by breeders.

Methods

DNA extraction, library construction and sequencing, and genome assembly

High molecular weight genomic DNA was extracted from young leaves of 14-day old seedlings of *Solanum aethiopicum* 'Shum' accession 303, which had been previously and repeatedly selfed to ensure homozygosity. Shum 303 is a selection of African eggplant from Uganda, with green fruits and pigmented stem and leaf veins. DNA was extracted using a modified CTAB protocol, as previously described [59]. Briefly, 2.5 g fresh leaf tissue was flash-frozen in liquid nitrogen and ground to a fine powder, before adding 15 ml of 2x extraction buffer (100 mM Tris-HCl pH 8.0, 1.4 M NaCl, 20 mM EDTA, 2% w/v CTAB, 10 μl/ml β-mercaptoethanol), then incubated at 65°C. One volume of chloroform:isoamyl alcohol (24:1) (ChIA) was added and mixed and the sample was centrifuged twice. The aqueous phase was precipitated overnight and the washed pellet was treated with RNaseA. A repeat chloroform extraction was performed, as above, to remove RNaseA and any other contaminants. The aqueous phase was collected and DNA was precipitated and washed with ethanol. DNA was allowed to dry, then was resuspended in 100 μl elution buffer.

High molecular weight DNA was fragmented and used to construct paired-end libraries

with insert sizes of 250 bp, 500 bp, 2 kb, 6 kb, 10 kb and 20 kb, following standard Illumina protocols. The libraries were sequenced on an Illlumina HiSeq 2000 platform, resulting in a total of 242.61 Gb raw reads. Filtering of duplicated, low quality reads and reads with adapters was done using SOAPfilter (version 2.2, an application included in the SOAPdenovo2 package, RRID:SCR_014986) [49] with the parameters "-M 2, -f 0, -p". Reads with ≥40% low quality bases or with ≥10% uncalled bases ('N') were filtered. We used 17 *k*-mer counts [21] of high-quality reads from small insert libraries to evaluate the genome size and heterozygosity using GCE [60] and Kmergenie [61]. We assembled the genome using Platanus (Platanus, RRID:SCR_015531)[22].

Genomic DNA used for resequencing was extracted from young leaves of 65 accessions. DNA was sheared into small fragments of ~200 bp and used to construct paired-end libraries, following standard BGI protocols as previously described [62], and subsequently sequenced on a BGI-500 sequencer. Briefly, the DNA fragments were ligated to BGISEQ-500 compatible adapters, followed by an index polymerase chain reaction (PCR) amplification, the products of which were then pooled and circularised for sequencing on the BGISEQ-500 (BGI, Shenzhen, China). Ultra-deep data were produced for each accession, with coverage ranging from ~45 to ~75X (Supplementary Table 20).

RNA extraction, library construction and sequencing

For RNA extraction, seeds of ‘Gilo’ and ‘Shum’ inbred lines were obtained from Uganda Christian University. The seeds were planted in a screenhouse at the BecA-ILRI Hub (Nairobi, Kenya) in polyvinylchloride (PVC) pots (13 cm height and 11.5 cm diameter) containing sterile forest soil and farmyard manure (2:1). The seedlings were later transplanted into larger PVC pots of 21 cm height and 14 cm diameter. Plants were raised in a screenhouse at 21–23°C and 11–13°C day and night temperatures, respectively (average 12 light hours per day). The plants were regularly watered to maintain moisture at required capacity.

Two plants were selected randomly from each of ‘Gilo’ and ‘Shum’ accessions and were tagged at the seedling stage for tissue sampling. Fresh tissues were sampled from each of the tagged plants and flash-frozen in liquid nitrogen immediately. Total RNA was extracted from the frozen tissues using the ZR Plant RNA MiniprepTM Kit (Zymo Research, CA, USA), according to the manufacturer’s instructions. RNA integrity was evaluated by electrophoresis in denaturing agarose gel (1% agarose, 5% formamide, 1X TAE) stained with 3x Gel Red (Biotium Inc., CA, USA). RNA was quantified using the Qubit RNA Assay Kit (Life Technologies, Thermo Fisher Scientific Inc.). Ribosomal RNA (rRNA) was removed from 4 µl of total RNA from each sample using the Epicentre Ribo-zero™ rRNA Removal Kit (Epicentre, Madison, WI, USA). The rRNA-depleted RNA was then used to generate strand-specific RNA-seq libraries using TruSeq® Stranded mRNA Kit (Illumina, San Diego, CA, USA). Twenty mRNA libraries were prepared, multiplexed (10 samples at a time) and sequenced as paired-end reads on the MiSeq (Illumina) platform at the BecA-ILRI Hub. Similar to the

process of filtering genomic reads, SOAPfilter software [49] was used, with the parameters “-M 2, -f 0, -p” to filter low quality reads and adapter sequences. Reads with ≥40% low quality bases or with ≥10% uncalled bases (‘N’) were filtered out.

Repeat annotation

Tandem repeats were searched in the genome using Tandem Repeats Finder (TRF, version 4.04) [63]. Transposable elements (TEs) were identified by a combination of homology-based and *de novo* approaches. Briefly, the assembly was aligned to a known repeats database (Repbase16.02) using RepeatMasker (RRID:SCR_012954) and RepeatProteinMask (version 3.2.9) [64] at both the DNA and protein level. In the *de novo* approach, RepeatModeler (version 1.1.0.4, RRID:SCR_015027) [65] was employed to build a *de novo* repeat library using the *S. aethiopicum* assembly, in which redundancies were filtered out. TEs in the genome were then identified by RepeatMasker [64]. Long terminal repeats (LTR) were identified using LTRharvest [29], with the criterion of 75% similarity on both sides. LTRdigest [30] was used to identify the internal elements of LTR-Rs with the eukaryotic tRNA library [66]. Identified LTR-Rs including intact poly purine tracts and primer binding sites with LTR-Rs on both sides were considered to be the final intact LTR-Rs. These were then classified into superfamilies, *Gypsy* and *Copia*, by querying against Repbase 16.02 [67].

Annotation of gene models and ncRNA

Gene models were predicted using a combination of *de novo* prediction, homology search and RNA-aided annotation. Augustus software (RRID:SCR_008417) [68] was used to perform *de novo* prediction after the annotated repeats were masked in the assembly. To search for homologous sequences, protein sequences of four closely related species (*S. lycopersicum*, *S. tuberosum*, *Capsisum annuum* and *Nicotiana sylvestris*), together with *Arabidopsis thaliana*, were used as query sequences to search the reference genome sequence using TBLASTN (RRID:SCR_011822) [69] with the e-value ≤1e-5. Regions mapped by these query sequences were subjected to GeneWise (RRID:SCR_015054) [70], together with their flanking sequences (1000 bp) to identify the positions of start/stop codons and splicing. For RNA-aided annotation, RNA-seq data from different tissues of *S. aethiopicum* were mapped to the genome assembly of *S. aethiopicum* using HISAT (RRID:SCR_015530) [71]. Mapped reads were then assembled using StringTie (RRID:SCR_016323) [72]. GLEAN software [73] was used to integrate mapped transcripts from different sources to produce a consensus gene set. tRNAscan-SE (RRID:SCR_010835) [74] was performed to search for reliable tRNA positions. snRNA and miRNA were detected by searching the reference sequence against the Rfam database (RRID:SCR_007891) [75] using BLAST [69]. rRNAs were detected by aligning with BLASTN (RRID:SCR_004870) [69] against known plant rRNA sequences [76]. For functional annotation, protein sequences were searched against Swissprot, TrEMBL, KEGG (release 88.2), InterPro, Gene Ontology, COG and Non-redundant protein NCBI databases [77–82].

Gene family analysis

Proteins of *S. aethiopicum*, *S. tuberosum* (PGSC v3.4) [18], *S. lycopersicum* (v2.3) [19], *C. annuum* (PGA v.1.6) [24] and *S. melongena* (Sme2.5.1) [83] were selected to perform all-against-all comparisons using BLASTP (RRID:SCR_001010)[69], with an e-value cutoff of ≤1e-5. OrthoMCL (RRID:SCR_007839) [26] and the default MCL inflation parameter of 1.5 were used to define the gene families. Single-copy families were selected to perform multiple sequence alignment using MAFFT (RRID:SCR_011811) [84]. Four-fold degenerate sites were picked and used to construct a phylogenetic tree based on the maximum-likelihood method by PhyML (RRID:SCR_014629) [85], with *C. annuum* as the outgroup. WGD analysis was achieved by identifying colinearity blocks by paralog gene pairs in MCscanX, with default parameters [27]. Each aligned paralog gene pair was concatenated to a super-sequence in one colinearity block and 4dTv (transversion of fourfold degenerate site) values of each block were calculated. We also determined the distribution of 4DTv values to estimate the speciation between species or WGD events. The divergence time of *S. aethiopicum* was estimated using the MCMCtree program [86], with the constructed phylogenetic trees and the divergence time of *C. annuum* [24] and *S. tuberosum* [18].

Analysis of LTR-Rs

Insertion times of identified, intact LTR-Rs were estimated based on the sequence divergence between the 5′ and 3′ LTR of each element. The nucleotide distance K between one pair of LTR-Rs was calculated using the Kimura 2-parameter method in Distmat (EMBOSS package) [87]. An average base substitution rate of 1.3e-8 [31] was used to estimate the insertion time, based on the formula:

$$T = K / 2r \text{ [15]}$$

Transcriptomic data were used to analyse the activity of intact LTR-Rs. After filtering and removing low quality reads, high quality reads from each were mapped against the full length LTR-R sequence using BWA-MEM software [88], with default parameters. Expression levels of intact LTR-Rs were calculated using EdgeR [89] and visually presented using pheatmap in R [90].

Analysis of NB-containing genes

Nucleotide-binding (NB) domain-containing genes in the *S. aethiopicum* genome were identified using a method previously described [15, 91]. Briefly, the HMM profile of the NB-ARC domain (PF00931) was used as a query to perform an HMMER search (version 3.2.1, RRID:SCR_005305 [92]) against protein sequences of tomato, potato, hot pepper [18, 19, 24] and annotated sequences of *S. aethiopicum*, with an e-value cut-

off of ≤1e-60. Aligned NB-ARC domain sequences of *S. aethiopicum* were extracted and used to build the *S. aethiopicum*-specific HMM model. NB-ARC domain sequences of tomato, potato and hot pepper were mapped as the query sequences against the *S. aethiopicum* genome using TBLASTN [69], with an e-value cut-off of ≤1e-4 using GeneWise software [70] to identify candidate NB-containing genes at the whole genome level. Final NB-containing genes were confirmed by searching the genome with an *S. aethiopicum*-specific NB-ARC HMM model, constructed with an e-value cut-off of ≤1e-4. Retroduplicated NLRs were identified according to the method described by Kim et al. (2017) [15]. Phylogenetic trees for *S. aethiopicum* and *S. melongena* NB-containing genes were constructed using FastTree (RRID:SCR_015501) [93], with default parameters.

SNP calling

The Genome Analysis Toolkit (GATK) pipeline (RRID:SCR_001876) [94] was used to call SNPs and indels. Briefly, low quality, duplicated and adapter-contaminated reads were filtered using SOAPfilter (version 2.2) [49] before further processing. To reduce the compute time, scaffolds in the assembly were sequentially linked into 24 pseudo-chromosomes, in which the original scaffolds were separated by 100 Ns, before mapping reads using BWA (RRID:SCR_010910) [88], with default parameters. Picard-tools [95] and SAMtools (RRID:SCR_002105) [96] were used to further process the alignment outputs, including sorting and marking of duplicates. After alignment and

sorting, the GATK pipeline (version 4.0.11.0) was used to call SNPs by sequentially implementing the following modules: RealignerTargetCreator, IndelRealigner, UnifiedGenotyper, samtools mpileup, VariantFiltration, BaseRecalibrator, AnalyzeCovariates, PrintReads and HaplotypeCaller, with default parameters. This pipeline produced a file in gvcf format, which displayed the called SNPs and indels filtered according to genotype information. The file was then analysed using PLINK software [97] for quality control, with “GENO>0.05, MAF<0.1, HWE test p-value ≤0.0001” parameters (GENO: Maximum per-SNP missing; MAF: Minor allele frequency; HWE: Hardy-Weinberg disequilibrium p-value). The loci of these SNPs and indels were anchored back to the original scaffolds and annotated using SnpEff [98]. To identify structural variations (SVs), sample information was added using AddOrReplaceReadGroups, a module of Picard-tools, and SVs were detected using DiscoverVariantsFromContigAlignmentsSAMSpark, a GATK module.

Population analysis

A maximum-likelihood phylogenetic tree was constructed, based on the genotypes at all the SNP loci using FastTree [93], with default parameters. To perform principal component analysis (PCA), Beagle4.1 [99] was used to impute the unphased genotypes. All imputed and identified genotypes at SNP loci were pooled and finalised using PLINK [97] and ReSeqTools [100], which were then subjected to PCA using GCTA software [101]. The population was clustered using ADMIXTURE software [39], with

K (the expected number of clusters) increasing from 2 to 9. The K value with the minimum cross-validation error was eventually selected.

Genome-wide linkage disequilibrium (LD) was calculated for populations of different groups using Haploview [102] in windows of 2,000 kb. Briefly, the correlation coefficient (r^2) between SNP pairs in a non-overlapping sliding 1 kb bin was calculated and then averaged within bins.

Candidate regions under selection were identified by comparing polymorphism levels – measured by *ROD*, as well as by F_{ST} – between 'Gilo', 'Shum' and '*Solanum anguivi*' groups. *ROD* was calculated using the formula:

$$ROD = 1 - \pi_{cul}/\pi_{wild}$$

where π_{cul} and π_{wild} denote the nucleotide diversity within the cultivated and wild populations, respectively.

F_{ST} measurement was calculated according to the formula:

$$F_{ST} = (\pi_{between} - \pi_{within}) / \pi_{between}$$

where $\pi_{between}$ and π_{within} represent the average number of pairwise differences between two individuals sampled from different or the same population.

Construction of pan- and core-genome

To build a gene set including as many *S. aethiopicum* genes as possible, we assembled

contigs of all 65 resequenced accessions individually using SOAPdenovo2 [49]. The assembled contigs from each group ('Gilo', 'Shum' and '*S. anguivi*') were then merged. CD-HIT-EST [50] was used to eliminate redundancy and generate the final dataset of pan-genomes for each group. Similarly, all these contigs were merged into a pan-genome of *S. aethiopicum*. Gene models were predicted from these contigs as described above and their functions were also annotated.

Availability of supporting data and materials

The raw sequence data from our genome project was deposited in the NCBI Sequence Read Archive with BioProject number PRJNA523664 and in the CNGB Nucleotide Sequence Archive database under project accession number CNP0000317. Assembly and annotation of the *S. aethiopicum* genome are available in GigaDB [103]. All supplementary figures and tables are provided as Additional Files.

Additional files

Supplementary Tables-1.docx

Supplementary Tables-2.xlsx

Supplementary Figures.docx

List of abbreviations

4DTV, four-fold degenerative third-codon transversion; BUSCO, Benchmarking Universal Single-Copy Orthologs; CEG, core embryophyta gene; CV, cross-validation; GATK, Genome Analysis Toolkit; LTR, long terminal repeat; LINE, long interspersed element; LD, Linkage disequilibrium; MYA, million years ago; PSMC, pairwise sequential Markovian coalescent model; PCA, principal-component analysis; SINE, short interspersed element; TE, transposable elements; WGD, whole genome duplication; WGS, whole-genome shotgun.

Consent for publication

Not applicable.

Competing interests

The authors declare that they have no competing interests.

Funding

This work was supported by the National Natural Science Foundation of China (grant number 31601042), the Science, Technology and Innovation Commission of Shenzhen Municipality (grant numbers JCYJ20151015162041454 and JCYJ20160331150739027) and by the Guangdong Provincial Key Laboratory of Genome Read and Write (grant number 2017B030301011).

Authors contributions:

D.A.O., X.X., A.V., X.L., J.W. and H.Y. conceived the project; D.A.O., F.S., E.B.K., A.V., S.C. and H.L. managed and supervised the work; B.S. and Y.F. managed the samples at BGI; B.S. and Y.F assembled the whole genome, Y.F and Y.S. annotated the genome. S.N.K., S.M. and R.K. extracted high molecular weight DNA. H.L. and S.P. constructed DNA libraries and sequenced the genome. S.N.K. and S.M. prepared RNA libraries and sequenced the transcriptome. J.N. and S.N.K. assembled and analysed the transcriptome. Y.S and B.S. performed the analysis of gene families, LTR evolution and transcriptomic data; Y.F., B.S., and Y.P.N.K. extracted DNA for re-sequencing samples. Y.F. and B.S. analysed the resequencing data; Y.S., Y.F. and B.S. collected datasets required for the genome annotation and analyses. B.S, X.L., Y.S., D.A.O., and Y.F. wrote and revised the manuscript.

Acknowledgements

We acknowledge Uganda Christian University for providing seeds of the African eggplant.

References

1. Sunseri F, Polignano GB, Alba V, Lotti C, Bisignano V, Mennella G, et al. Genetic diversity and characterization of African eggplant germplasm collection. African Journal of Plant Science. 2010;4:231-41.
2. Adeniji O, Kusolwa P and Reuben S. Genetic diversity among accessions of Solanum aethiopicum L. groups based on morpho-agronomic traits. Plant Genetic Resources. 2012;10 3:177-85.
3. Plazas M, Andújar I, Vilanova S, Gramazio P, Herraiz FJ and Prohens J. Conventional and phenomics characterization provides insight into the diversity and relationships of hypervariable scarlet (Solanum aethiopicum L.) and gboma (S. macrocarpon L.) eggplant complexes. Frontiers in plant science. 2014;5:318.
4. Prohens J, Plazas M, Raigón MD, Seguí-Simarro JM, Stommel JR and Vilanova S. Characterization of interspecific hybrids and first backcross generations from crosses between two cultivated eggplants (Solanum melongena and S. aethiopicum Kumba group) and implications for eggplant breeding. Euphytica. 2012;186 2:517-38.
5. Toppino L, Valè G and Rotino GL. Inheritance of Fusarium wilt resistance introgressed from Solanum aethiopicum Gilo and Aculeatum groups into cultivated eggplant (S. melongena) and development of associated PCR-based markers. Molecular Breeding. 2008;22 2:237-50.

6. FAO. http://www.fao.org/traditional-crops/africangardenegg/en/?amp%3Butm_medium=social%20media&amp%3Butm_campaign=unfaopinterest. Accessed 19 Aug 2019.

7. Schippers RR. African indigenous vegetables: an overview of the cultivated species. 2000.

8. Maundu P, Achigan-Dako E and Morimoto Y. Biodiversity of African vegetables. African indigenous vegetables in urban agriculture. 2009:65-104.

9. Gramazio P, Blanca J, Ziarsolo P, Herraiz FJ, Plazas M, Prohens J, et al. Transcriptome analysis and molecular marker discovery inSolanum incanumandS. aethiopicum, two close relatives of the common eggplant (*Solanum melongena*) with interest for breeding. BMC Genomics. 2016;17 1:300.

10. Mennella G, Rotino GL, Fibiani M, D'Alessandro A, Francese G, Toppino L, et al. Characterization of health-related compounds in eggplant (*Solanum melongena* L.) lines derived from introgression of allied species. J Agric Food Chem. 2010;58 13:7597-603.

11. Cappelli C, Stravato V, Rotino G and Buonaurio R. Sources of resistance among Solanum spp. to an Italian isolate of Fusarium oxysporum f. sp. melongenae. In: *Eucarpia Meeting on genetics and breeding of Capsicum & Eggplant IX* 1995.

12. Fock I. Source of resistance against Ralstonia solanacearum in fertile somatic hybrids of eggplant (Solanum melongena L.) with Solanum aethiopicum L. Plant Science An International Journal of Experimental Plant Biology. 2001;160 2:301-13.

13. Gisbert C, Prohens J, Raigón MD, Stommel JR and Nuez F. Eggplant relatives as sources of variation for developing new rootstocks: Effects of grafting on eggplant yield and fruit apparent quality and composition. Scientia Horticulturae. 2011;128 1:14-22.

14. Rizza F, Mennella G, Collonnier C, Sihachakr D, Kashyap V, Rajam M, et al. Androgenic

dihaploids from somatic hybrids between Solanum melongena and S. aethiopicum group gilo as a source of resistance to *Fusarium oxysporum f. sp*. melongenae. Plant Cell Reports. 2002;20 11:1022-32.

15. Kim S, Park J, Yeom SI, Kim YM, Seo E, Kim KT, et al. New reference genome sequences of hot pepper reveal the massive evolution of plant disease-resistance genes by retroduplication. Genome Biology. 2017;18 1:210.

16. Sierro N, Battey JN, Ouadi S, Bovet L, Goepfert S, Bakaher N, et al. Reference genomes and transcriptomes of *Nicotiana sylvestris* and *Nicotiana tomentosiformis*. Genome Biology. 2013;14 6:R60.

17. Qin C, Yu C, Shen Y, Fang X, Chen L, Min J, et al. Whole-genome sequencing of cultivated and wild peppers provides insights into Capsicum domestication and specialization. Proc Natl Acad Sci U S A. 2014;111 14:5135-40.

18. Consortium TPGS. Genome sequence and analysis of the tuber crop potato. Nature. 2011;475 7355:189-95.

19. Consortium TG. The tomato genome sequence provides insights into fleshy fruit evolution. Nature. 2012;485 7400:635.

20. Bombarely A, Moser M, Amrad A, Bao M, Bapaume L, Barry CS, et al. Insight into the evolution of the Solanaceae from the parental genomes of Petunia hybrida. Nat Plants. 2016;2 6:16074.

21. Moscone EA, Baranyi M, Ebert I, Greilhuber J, Ehrendorfer F and Hunziker AT. Analysis of Nuclear DNA Content in Capsicum (Solanaceae) by Flow Cytometry and Feulgen Densitometry. Annals of Botany. 2003;92 1:21.

22. Kajitani R, Toshimoto K, Noguchi H, Toyoda A, Ogura Y, Okuno M, et al. Efficient de novo assembly of highly heterozygous genomes from whole-genome shotgun short reads. Genome Research. 2014;24 8:1384-95.

23. Initiative AG. Analysis of the genome sequence of the flowering plant *Arabidopsis thaliana*. Nature. 2000;408 6814:796-815.

24. Kim S, Park M, Yeom SI, Kim YM, Lee JM, Lee HA, et al. Genome sequence of the hot pepper provides insights into the evolution of pungency in Capsicum species. Nature Genetics. 2014;46 3:270-8.

25. Waterhouse RM, Seppey M, Simão FA, Manni M, Ioannidis P, Klioutchnikov G, et al. BUSCO applications from quality assessments to gene prediction and phylogenomics. Molecular Biology & Evolution. 2017;35 3.

26. Li L, Stoeckert CJ and Roos DS. OrthoMCL: identification of ortholog groups for eukaryotic genomes. Genome Research. 2003;13 9:2178-89.

27. Wang Y, Tang H, DeBarry JD, Tan X, Li J, Wang X, et al. MCScanX: a toolkit for detection and evolutionary analysis of gene synteny and collinearity. Nucleic acids research. 2012;40 7:e49-e.

28. Jaillon O, Aury J-M, Noel B, Policriti A, Clepet C, Casagrande A, et al. The grapevine genome sequence suggests ancestral hexaploidization in major angiosperm phyla. Nature. 2007;449 7161:463.

29. Ellinghaus D, Kurtz S and Willhoeft U. LTRharvest, an efficient and flexible software for de novo detection of LTR retrotransposons. BMC bioinformatics. 2008;9 1:18.

30. Steinbiss S, Willhoeft U, Gremme G and Kurtz S. Fine-grained annotation and classification of

de novo predicted LTR retrotransposons. Nucleic acids research. 2009;37 21:7002-13.

31. Ma J and Bennetzen JL. Rapid recent growth and divergence of rice nuclear genomes. Proceedings of the National Academy of Sciences. 2004;101 34:12404-10.

32. McHale L, Tan X, Koehl P and Michelmore RW. Plant NBS-LRR proteins: adaptable guards. Genome Biology. 2006;7 4:212.

33. Yue JX, Meyers BC, Chen JQ, Tian D and Yang S. Tracing the origin and evolutionary history of plant nucleotide‐binding site–leucine‐rich repeat (NBS‐LRR) genes. New Phytologist. 2012;193 4:1049-63.

34. Xia R, Xu J, Arikit S and Meyers BC. Extensive Families of miRNAs and PHAS Loci in Norway Spruce Demonstrate the Origins of Complex phasiRNA Networks in Seed Plants. Molecular Biology & Evolution. 2015;32 11:2905-18.

35. Ratnaparkhe MB, Wang X, Li J, Compton RO, Rainville LK, Lemke C, et al. Comparative analysis of peanut NBS-LRR gene clusters suggests evolutionary innovation among duplicated domains and erosion of gene microsynteny. New Phytologist. 2011;192 1:164-78.

36. Lester R and Niakan L. Origin and domestication of the scarlet eggplant, Solanum aethiopicum, from S. anguivi in Africa. Solanaceae: Biology and systematics. 1986:433-56.

37. Barrett JC, Fry B, Maller J and Daly MJ. Haploview: analysis and visualization of LD and haplotype maps. Bioinformatics. 2004;21 2:263-5.

38. Pascual L, Desplat N, Huang BE, Desgroux A, Bruguier L, Bouchet JP, et al. Potential of a tomato MAGIC population to decipher the genetic control of quantitative traits and detect causal variants in the resequencing era. Plant Biotechnology Journal. 2015;13 4:565-77.

39. Alexander DH, Novembre J and Lange K. Fast model-based estimation of ancestry in unrelated

individuals. Genome research. 2009.

40. Li H and Richard D. Inference of Human Population History From Whole Genome Sequence of A Single Individual. Nature. 2012;475 7357:493-6.

41. Manning K and Timpson A. The demographic response to Holocene climate change in the Sahara. Quaternary Science Reviews. 2014;101:28-35.

42. Wang Y, Wu Y and Tang D. The autophagy gene, ATG18a, plays a negative role in powdery mildew resistance and mildew-induced cell death in *Arabidopsis*. Plant signaling & behavior. 2011;6 9:1408-10.

43. Suttangkakul A, Li F, Chung T and Vierstra RD. The ATG1/ATG13 protein kinase complex is both a regulator and a target of autophagic recycling in *Arabidopsis*. The Plant Cell. 2011;23 10:3761-79.

44. Larsen PB, Cancel J, Rounds M and Ochoa V. Arabidopsis ALS1 encodes a root tip and stele localized half type ABC transporter required for root growth in an aluminum toxic environment. Planta. 2007;225 6:1447.

45. Kim D-Y, Bovet L, Kushnir S, Noh EW, Martinoia E and Lee Y. AtATM3 is involved in heavy metal resistance in *Arabidopsis*. Plant physiology. 2006;140 3:922-32.

46. Giritch A, Herbik A, Balzer HJ, Ganal M, Stephan UW and Bäumlein H. A root-specific iron-regulated gene of tomato encodes a lysyl-tRNA-synthetase-like protein. European journal of biochemistry. 1997;244 2:310-7.

47. Perera IY, Hung C-Y, Moore CD, Stevenson-Paulik J and Boss WF. Transgenic Arabidopsis plants expressing the type 1 inositol 5-phosphatase exhibit increased drought tolerance and altered abscisic acid signaling. The Plant Cell. 2008;20 10:2876-93.

48. Cheng S, Gutmann B, Zhong X, Ye Y, Fisher MF, Bai F, et al. Redefining the structural motifs that determine RNA binding and RNA editing by pentatricopeptide repeat proteins in land plants. The Plant Journal. 2016;85 4:532-47.

49. Luo R, Liu B, Xie Y, Li Z, Huang W, Yuan J, et al. SOAPdenovo2: an empirically improved memory-efficient short-read de novo assembler. GigaScience. 2012;1 1:18.

50. Fu L, Niu B, Zhu Z, Wu S and Li W. CD-HIT: accelerated for clustering the next-generation sequencing data. Bioinformatics. 2012;28 23:3150-2.

51. Galindo-González L, Mhiri C, Deyholos MK and Grandbastien M-A. LTR-retrotransposons in plants: engines of evolution. Gene. 2017;626:14-25.

52. Kashkush K, Feldman M and Levy AA. Transcriptional activation of retrotransposons alters the expression of adjacent genes in wheat. Nature genetics. 2003;33 1:102.

53. Kashkush K and Khasdan V. Large-scale survey of cytosine methylation of retrotransposons and the impact of readout transcription from long terminal repeats on expression of adjacent rice genes. Genetics. 2007;177 4:1975-85.

54. Hollister JD, Smith LM, Guo Y-L, Ott F, Weigel D and Gaut BS. Transposable elements and small RNAs contribute to gene expression divergence between *Arabidopsis thaliana* and *Arabidopsis lyrata*. Proceedings of the National Academy of Sciences. 2011:201018222.

55. Hollister JD and Gaut BS. Epigenetic silencing of transposable elements: a trade-off between reduced transposition and deleterious effects on neighboring gene expression. Genome research. 2009.

56. Ma L-J, Van Der Does HC, Borkovich KA, Coleman JJ, Daboussi M-J, Di Pietro A, et al. Comparative genomics reveals mobile pathogenicity chromosomes in Fusarium. Nature.

2010;464 7287:367.

57. Song B, Morse D, Song Y, Fu Y, Lin X, Wang W, et al. Comparative genomics reveals two major bouts of gene retroposition coinciding with crucial periods of Symbiodinium evolution. Genome Biology and Evolution. 2017;9 8:2037-47.

58. Song B, Chen S and Chen W. Dinoflagellates, a unique lineage for retrogene research. Frontiers in microbiology. 2018;9:1556.

59. Stoffel K, van Leeuwen H, Kozik A, Caldwell D, Ashrafi H, Cui X, et al. Development and application of a 6.5 million feature Affymetrix Genechip® for massively parallel discovery of single position polymorphisms in lettuce (*Lactuca spp*.). BMC Genomics. 2012;13 1:185.

60. Liu B, Shi Y, Yuan J, Hu X, Zhang H, Li N, et al. Estimation of genomic characteristics by analyzing k-mer frequency in de novo genome projects. arXiv preprint arXiv:13082012. 2013.

61. Chikhi R and Medvedev P. Informed and automated k-mer size selection for genome assembly. Bioinformatics. 2013;30 1:31-7.

62. Jie Huang, Xinming Liang, Yuankai Xuan, Chunyu Geng, Yuxiang Li, Haorong Lu, Shoufang Qu, Xianglin Mei, Hongbo Chen, Ting Yu, Nan Sun, Junhua Rao, Jiahao Wang, Wenwei Zhang, Ying Chen, Sha Liao, Hui Jiang, Xin Liu, Zhaopeng Yang, Feng Mu, Shangxian Gao (2018). BGISEQ-500 WGS library construction. protocols.io http://dx.doi.org/10.17504/protocols.io.ps5dng6

63. Benson G. Tandem repeats finder: a program to analyze DNA sequences. Nucleic acids research. 1999;27 2:573-80.

64. Smit A, Hubley R and Green P. RepeatMasker Open-4.0. 2013–2015. 2015.

65. Smit AF and Hubley R. RepeatModeler Open-1.0. Available fom http://www repeatmasker org.

2008.

66. GtRNAdb. http://gtrnadb.ucsc.edu/. Accessed 19 Aug 2019.

67. Bao W, Kojima KK and Kohany O. Repbase Update, a database of repetitive elements in eukaryotic genomes. Mobile DNA. 2015;6 1:11.

68. Stanke M, Keller O, Gunduz I, Hayes A, Waack S and Morgenstern B. AUGUSTUS: ab initio prediction of alternative transcripts. Nucleic acids research. 2006;34 suppl_2:W435-W9.

69. Altschul SF, Gish W, Miller W, Myers EW and Lipman DJ. Basic local alignment search tool. Journal of molecular biology. 1990;215 3:403-10.

70. Birney E, Clamp M and Durbin R. GeneWise and genomewise. Genome research. 2004;14 5:988-95.

71. Kim D, Langmead B and Salzberg SL. HISAT: a fast spliced aligner with low memory requirements. Nature methods. 2015;12 4:357.

72. Pertea M, Pertea GM, Antonescu CM, Chang T-C, Mendell JT and Salzberg SL. StringTie enables improved reconstruction of a transcriptome from RNA-seq reads. Nature biotechnology. 2015;33 3:290.

73. Elsik CG, Mackey AJ, Reese JT, Milshina NV, Roos DS and Weinstock GM. Creating a honey bee consensus gene set. Genome biology. 2007;8 1:R13.

74. Lowe TM and Eddy SR. tRNAscan-SE: a program for improved detection of transfer RNA genes in genomic sequence. Nucleic acids research. 1997;25 5:955.

75. Kalvari I, Argasinska J, Quinones-Olvera N, Nawrocki EP, Rivas E, Eddy SR, et al. Rfam 13.0: shifting to a genome-centric resource for non-coding RNA families. Nucleic acids research. 2017;46 D1:D335-D42.

76. Vitales D, D'Ambrosio U, Gálvez F, Kovařík A, Garcia S. Third release of the plant rDNA database with updated content and information on telomere composition and sequenced plant genomes. Plant Systematics and Evolution. 2017;303:1115-1121.

77. Consortium U. UniProt: the universal protein knowledgebase. Nucleic acids research. 2018;46 5:2699.

78. Mitchell AL, Attwood TK, Babbitt PC, Blum M, Bork P, Bridge A, et al. InterPro in 2019: improving coverage, classification and access to protein sequence annotations. Nucleic Acids Research. 2018.

79. Ashburner M, Ball CA, Blake JA, Botstein D, Butler H, Cherry JM, et al. Gene Ontology: tool for the unification of biology. Nature genetics. 2000;25 1:25.

80. Consortium GO. Expansion of the Gene Ontology knowledgebase and resources. Nucleic acids research. 2016;45 D1:D331-D8.

81. Tatusov RL, Fedorova ND, Jackson JD, Jacobs AR, Kiryutin B, Koonin EV, et al. The COG database: an updated version includes eukaryotes. BMC bioinformatics. 2003;4 1:41.

82. Bairoch A and Apweiler R. The SWISS-PROT protein sequence database and its supplement TrEMBL in 2000. Nucleic acids research. 2000;28 1:45-8.

83. Hirakawa H, Shirasawa K, Miyatake K, Nunome T, Negoro S, Ohyama A, et al. Draft genome sequence of eggplant (*Solanum melongena* L.): the representative solanum species indigenous to the old world. DNA research. 2014;21 6:649-60.

84. Nakamura T, Yamada KD, Tomii K, Katoh K and Hancock J. Parallelization of MAFFT for large-scale multiple sequence alignments. Bioinformatics. 2018;1:3.

85. Guindon S, Dufayard J-F, Lefort V, Anisimova M, Hordijk W and Gascuel O. New algorithms

and methods to estimate maximum-likelihood phylogenies: assessing the performance of PhyML 3.0. Systematic biology. 2010;59 3:307-21.

86. Yang Z and Rannala B. Bayesian estimation of species divergence times under a molecular clock using multiple fossil calibrations with soft bounds. Molecular biology and evolution. 2005;23 1:212-26.

87. Rice P, Longden I and Bleasby A. EMBOSS: the European molecular biology open software suite. Trends in genetics. 2000;16 6:276-7.

88. Li H and Durbin R. Fast and accurate short read alignment with Burrows–Wheeler transform. bioinformatics. 2009;25 14:1754-60.

89. Robinson MD, McCarthy DJ and Smyth GK. edgeR: a Bioconductor package for differential expression analysis of digital gene expression data. Bioinformatics. 2010;26 1:139-40.

90. Kolde R and Kolde MR. Package 'pheatmap'. 2018.

91. Seo E, Kim S, Yeom S-I and Choi D. Genome-wide comparative analyses reveal the dynamic evolution of nucleotide-binding leucine-rich repeat gene family among Solanaceae plants. Frontiers in plant science. 2016;7:1205.

92. HMMER. http://hmmer.org/. Accessed 19 Aug 2019.

93. Price MN, Dehal PS and Arkin AP. FastTree 2–approximately maximum-likelihood trees for large alignments. PloS one. 2010;5 3:e9490.

94. GATK. https://software.broadinstitute.org/gatk/. Accessed 19 Aug 2019.

95. Picard. https://broadinstitute.github.io/picard/. Accessed 19 Aug 2019.

96. Li H, Handsaker B, Wysoker A, Fennell T, Ruan J, Homer N, et al. The sequence alignment/map format and SAMtools. Bioinformatics. 2009;25 16:2078-9.

97. Chang CC, Chow CC, Tellier LC, Vattikuti S, Purcell SM and Lee JJ. Second-generation PLINK: rising to the challenge of larger and richer datasets. GigaScience. 2015;4 1:7.

98. Cingolani P, Platts A, Wang LL, Coon M, Nguyen T, Wang L, et al. A program for annotating and predicting the effects of single nucleotide polymorphisms, SnpEff: SNPs in the genome of Drosophila melanogaster strain w1118; iso-2; iso-3. Fly. 2012;6 2:80-92.

99. Browning SR and Browning BL. Rapid and accurate haplotype phasing and missing-data inference for whole-genome association studies by use of localized haplotype clustering. The American Journal of Human Genetics. 2007;81 5:1084-97.

100. He W, Zhao S, Liu X, Dong S, Lv J, Liu D, et al. ReSeqTools: an integrated toolkit for large-scale next-generation sequencing based resequencing analysis. Genetics and Molecular Research. 2013;12 4:6275-83.

101. Yang J, Lee SH, Goddard ME and Visscher PM. Genome-wide complex trait analysis (GCTA): methods, data analyses, and interpretations. Genome-wide association studies and genomic prediction. Springer; 2013. p. 215-36.

102. Barrett JC, Fry B, Maller J, Daly MJ. Haploview: analysis and visualization of LD and haplotype maps. Bioinformatics. 2005;21:263-265.

103. Song B; Song Y; Fu Y; Kizito EB; Kamenya SN; Kabod PN; Liu H; Muthemba S; Kariba R; Njuguna J; Maina S; Stomeo F; Djikeng A; Hendre PS; Chen X; Chen W; Li X; Sun W; Wang S; Cheng S; Muchugi A; Jamnadass R; Shapiro H; Van Deynze A; Yang H; Wang J; Xu X; Odeny DA; Liu X (2019): Supporting data for "Draft genome sequence of Solanum aethiopicum provides insights into disease resistance, drought tolerance and the evolution of the genome". GigaScience Database. http://dx.doi.org/10.5524/100642

Tables

Table 1: Statistical data for the *Solanum aethiopicum* genome and gene annotation

Number of scaffolds	162,187
Total length of scaffolds	1.02 Gb
N50 of scaffolds	516.1 Kb
Longest scaffolds	2.94 Mp
Number of contigs	231,821
Total length of contigs	936 Mb
N50 of contigs	25.2 Kb
Longest contigs	366.2 kb
GC content	33.13%
Number of genes	34,906
Average/total coding sequence length	1104.3bp/38.5 Mb
Average exon/intron length	265.8bp/613.1 bp
Total length of transposable elements	805.7 Mb (78.23%)

Table 2: Statistical data for single nucleotide polymorphisms and indels of 65 accessions

Type	Class	Number	Percentage (%)
SNPs	Exon	393,882	2.12
	Intron	675,360	3.63

	Intergenic	17,552,823	94.29
	Synonymous	126,172	0.67
	Nonsynonymous	267,710	1.44
	**Total**	**18,614,838**	
Indels	Exon	32,519	1.62
	Intron	145,741	7.28
	Intergenic	1,821,530	91.11
	Frame shift	2,977	0.13
	**Total**	**1,999,241**	

Figure legends

Figure 1: Comparative analysis of the *Solanum aethiopicum* genome.

(**A**) Phylogenetic analysis of *Solanum melongena*, *S. lycopersicum*, *S. tuberosum*, *S. aethiopicum* and *Capsicum annuum* using single-copy gene families. The species differentiation time between *S. aethiopicum* and *S. melongena* was 2.6 million years. (**B**) Distribution of 4DTv distance, which showed two peaks around 0.25 and 1 (black line), representing two whole genome duplication events. (**C**) Venn diagram showing overlaps of gene families between *S. melongena*, *S. lycopersicum*, *S. tuberosum*, *S. aethiopicum* and *C. annuum*. A total of 465 gene families were unique to *S. aethiopicum* and 10,166 were common to the genomes of the five species.

Figure 2: Long terminal repeat retrotransposon (LTR-R) insertion time distribution and

the expression level of LTR-Rs in different tissues.

Insertion time distribution of total LTR-Rs (**A**), *Ty3/Gypsy* LTR-Rs (**B**) and *Ty1/Copia* LTR-Rs (**C**) of *Capsicum annuum, Solanum melongena*, *S. lycopersicum* and *S. aethiopicum*. The x- and y-axes, respectively, indicate the insertion time and the frequency of inserted LTR-Rs. Expression levels of LTR-Rs in flower (**D**), fruit (**E**), leaf (**F**) and root (**G**) tissues.

Figure 3: LTR-R captured and disrupted genes.

(**A**) The distribution of ages of LTR-R captured and disrupted genes. (**B**) GO enrichment analysis between the LTR-R captured and disrupted gene set. (**C**) GO terms enriched in LTR-R captured genes that are specifically and highly expressed in various tissues, including leaf, flower, root and fruit. (**D**) Phylogenetic tree of the nucleotide-binding, leucine rich repeat-related (NLR) gene in *Solanum aethiopicum* and *S. melongena*.

Figure 4: Single nucleotide polymorphisms (SNPs), indel and linkage disequilibrium (LD) decay for 'Gilo', 'Shum' and '*S. anguivi*' groups.

(**A**) SNPs numbering 2,019,539 (10.85%%), 4,747,418 (25.50%) and 587,885 (3.16%) were unique to 'Gilo', 'Shum' and '*S. anguivi*', respectively. Most (93.13%) of SNPs in '*S. anguivi*' were shared with either 'Gilo' or 'Shum'. (**B**) Indels amounting to 14.06%, 28.96% and 2.76% were unique to 'Gilo', 'Shum' and '*S. anguivi*', respectively and, like the SNP statistics in these groups, 92.62% of indels in '*S. anguivi*'

were shared with either ‘Gilo’ or ‘Shum’. (**C**) LD estimation revealed that r^2 reaches the half maximum value at ~150 kb.

Figure 5: Population structure and demography of *Solanum aethiopicum*.

(**A**) A maximum-likelihood phylogenetic tree and population structure constructed using the full set of single nucleotide polymorphisms (SNPs). (**B**) Principal component analysis (PCA). (**C**) Pairwise sequential Markovian coalescent (PSMC) model analysis indicated a distinct demographic history of *S. aethiopicum* from 10,000 to 100 years ago, in which a bottleneck was shown around 4,000–5,000 years ago, followed by an immediate expansion of population size.

Figure 1

Click here to access/download;Figure;Figure 1.pdf

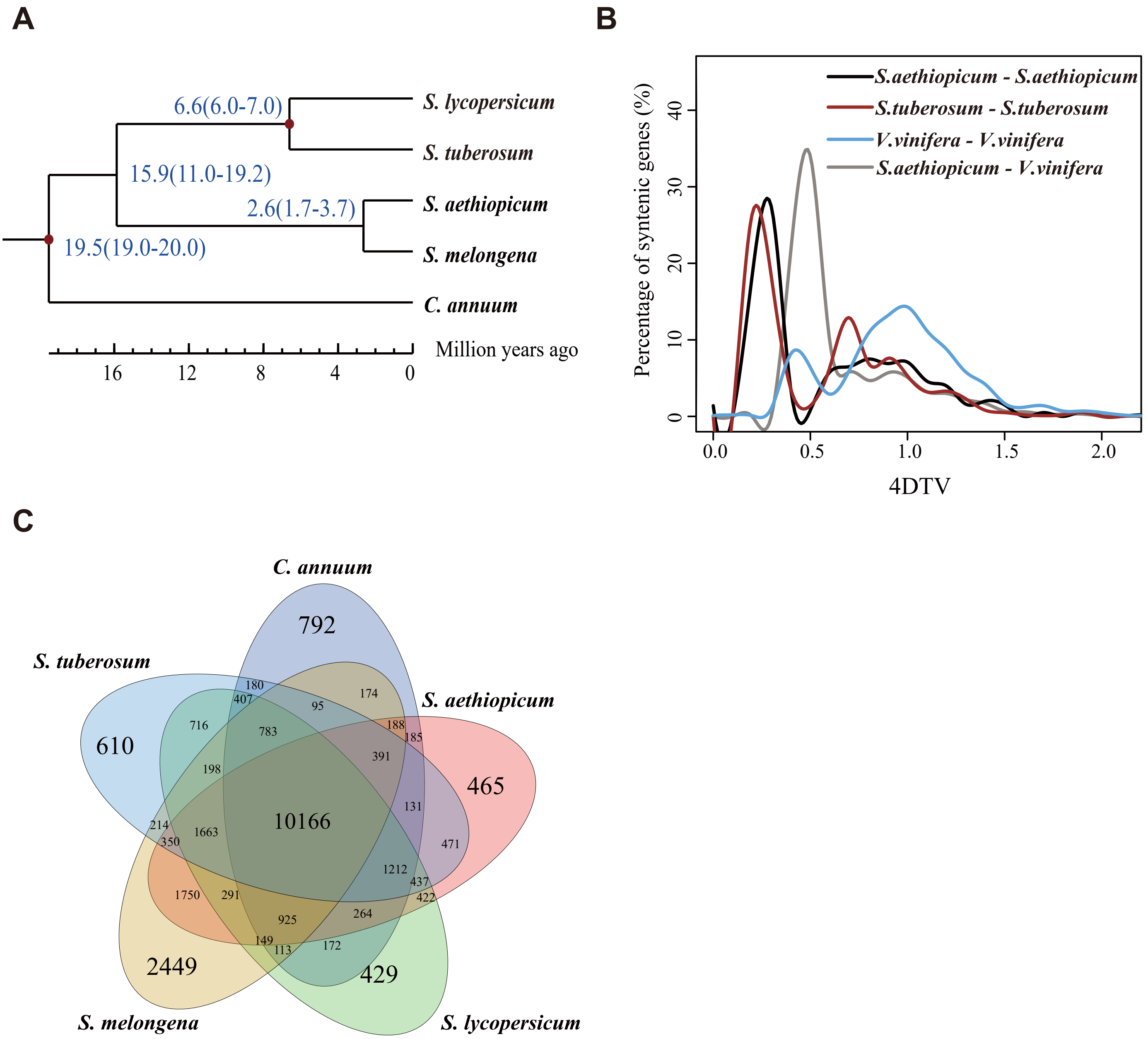

Figure 2

Click here to access/download;Figure;Figure 2.pdf

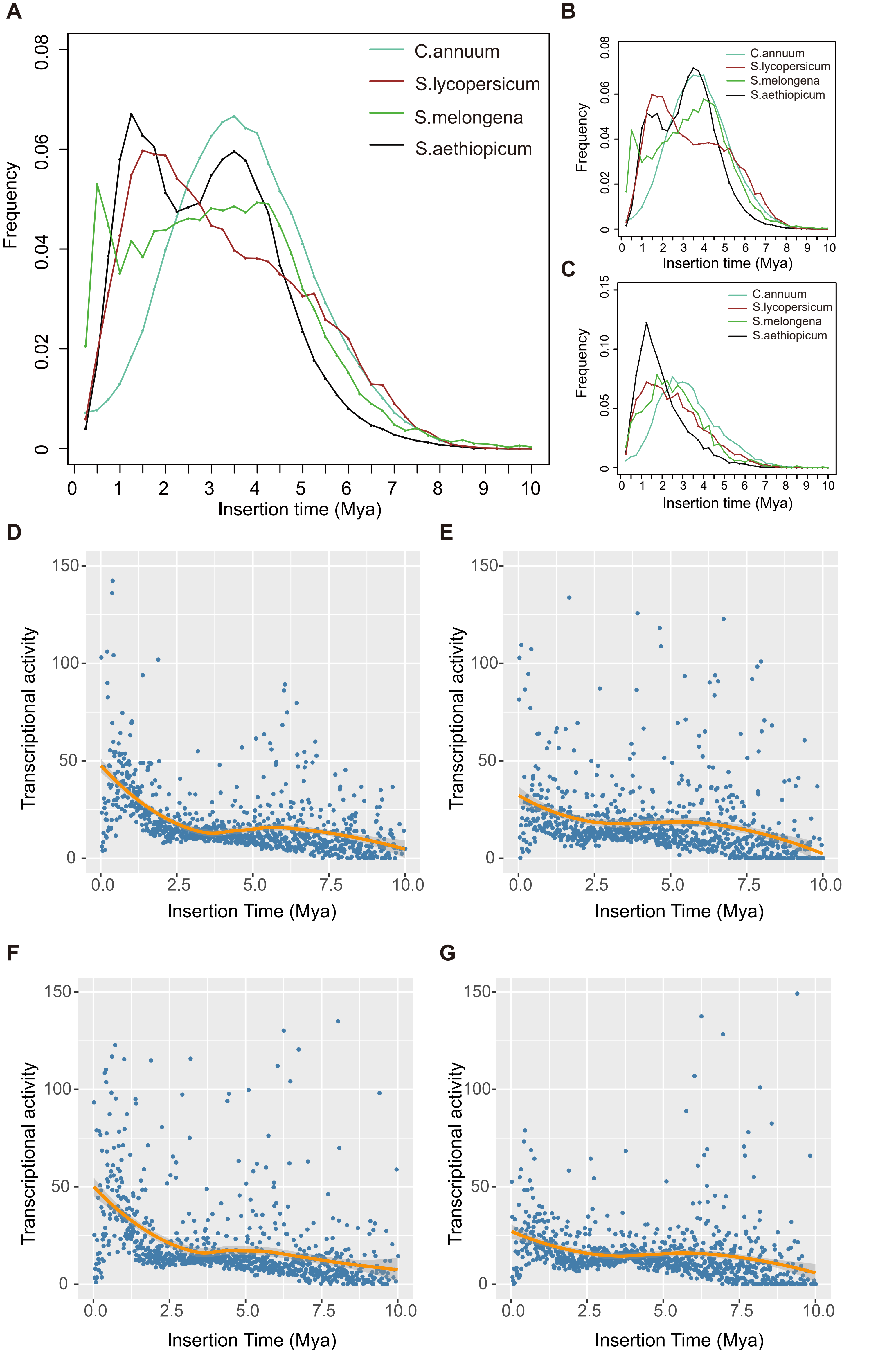

Click here to access/download;Figure;Figure 3.pdf

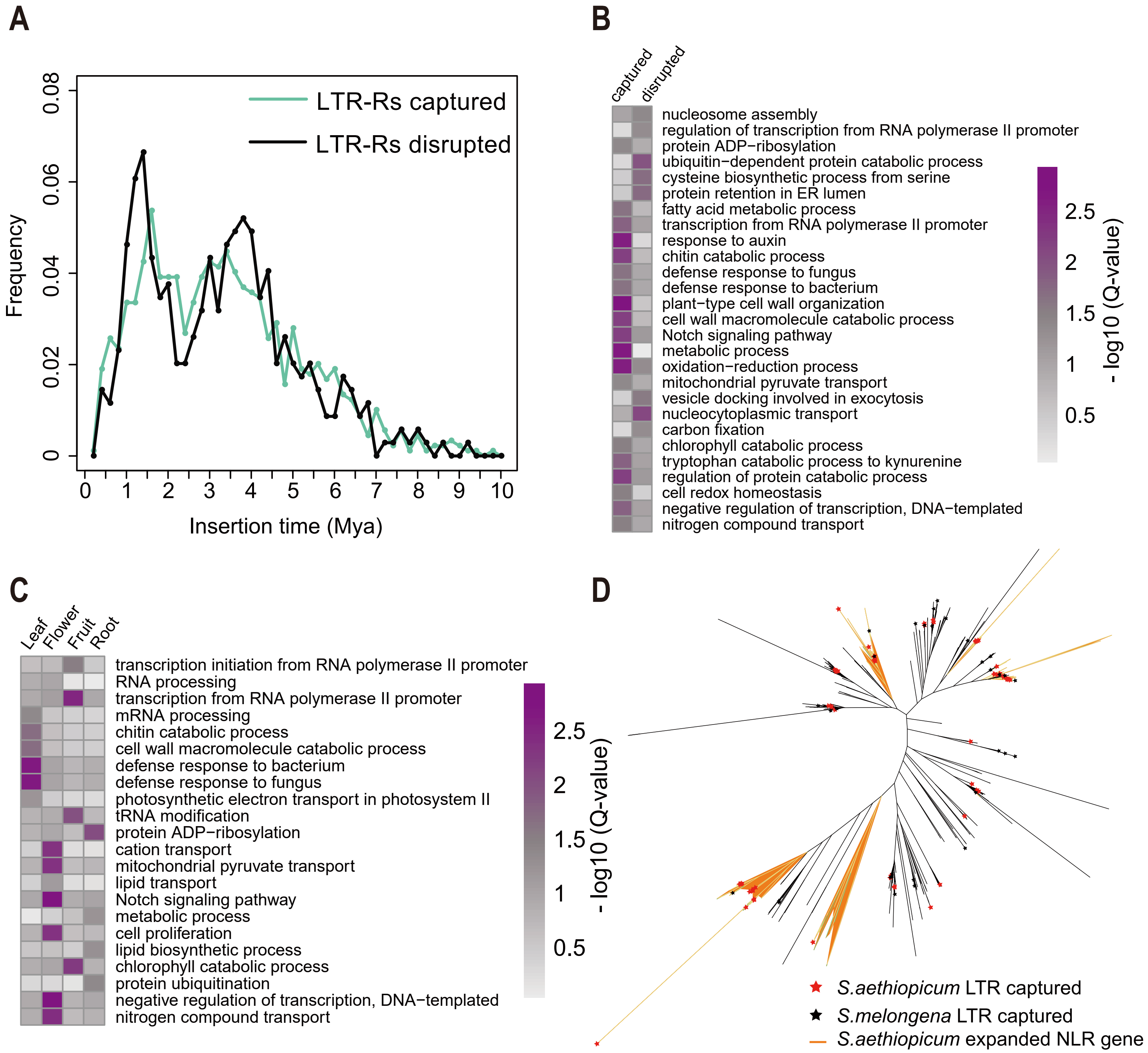

Figure 4

Click here to access/download;Figure;Figure 4.pdf

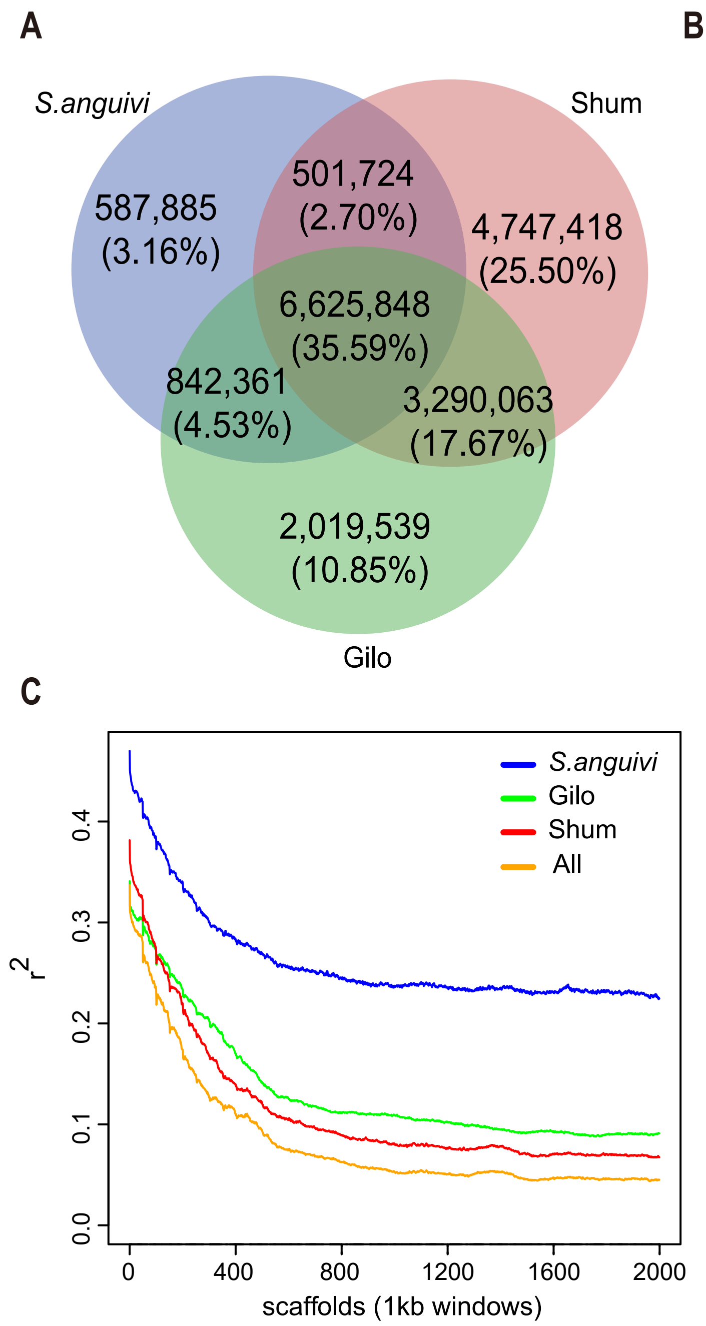

Supplementary Tables 1

Click here to access/download
Supplementary Material
Supplementary Tables 1.docx

Click here to access/download
Supplementary Material
Supplementary Tables 2.xlsx

Click here to access/download
Supplementary Material
Supplementary Figures.docx
