# Supplementary material for: Draft genome sequence of Solanum aethiopicum provides insights into disease resistance, drought tolerance, and the evolution of the genome
Source: Gigascience. 2019 Oct 1;8(10):giz115. doi: 10.1093/gigascience/giz115 (PMC6771550; doi:10.1093/gigascience/giz115)
Supplement: giz115_Supplemental_Files [file giz115_supplemental_files.zip › Supplementary Figures_final.docx]

**Supplementary Figure 1.** 17-mer analysis for estimating the *S. aethiopicum* genome size. The peak of distribution is about 38X coverage, then the genome size can be estimated as ~1.16 Gb (Genome Size=K-mer number/Peak depth). The small peak at 1/2 the peak depth shows the high heterozygous rate of the genome.


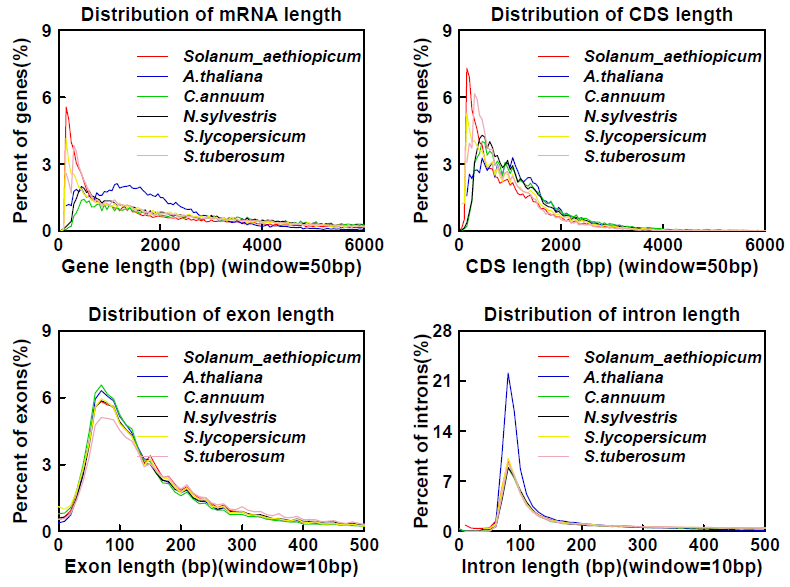


**Supplementary Figure 2.** Distributions of the gene model for four categories in the relative species.


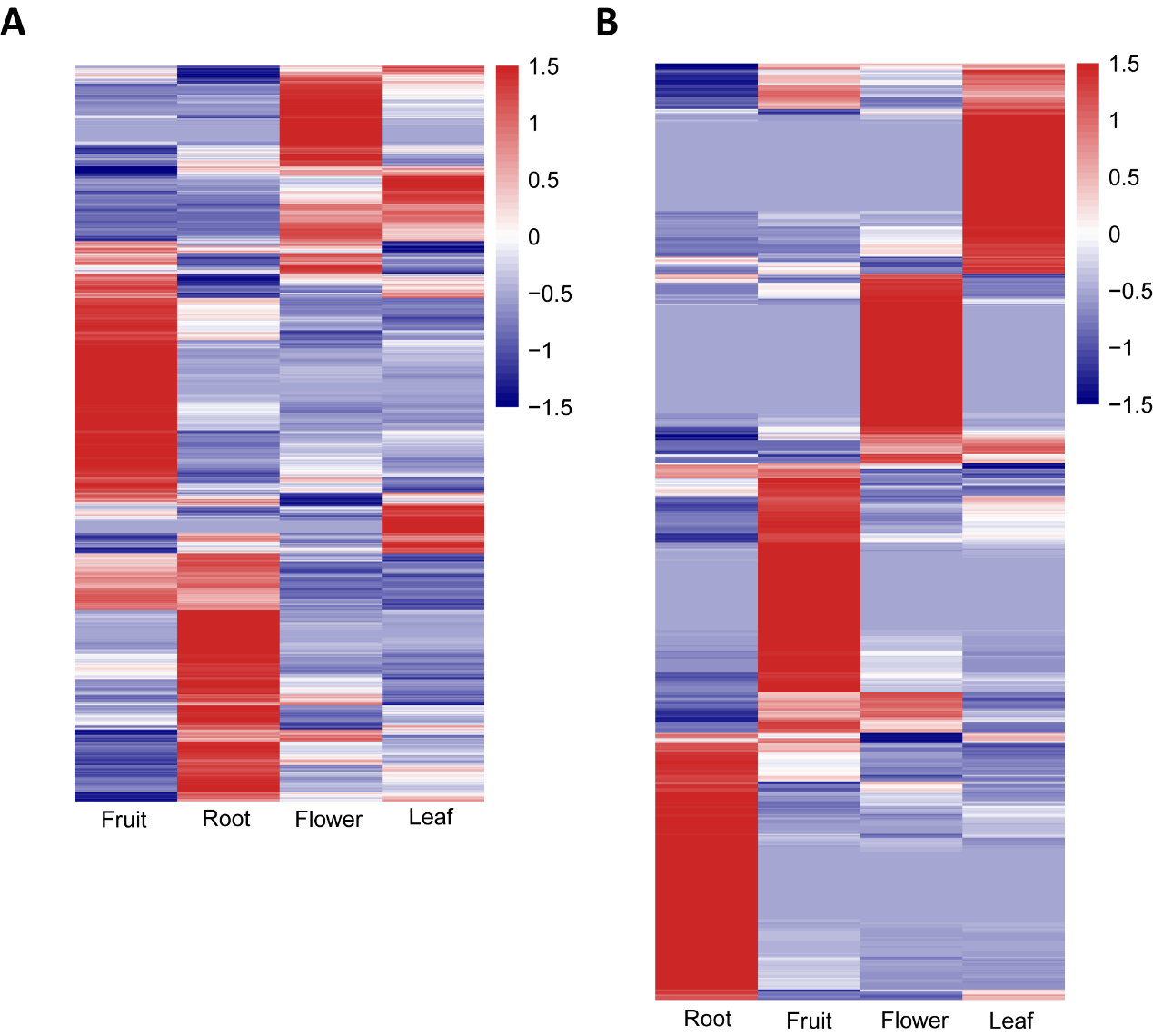


**Supplementary Figure 3.** Distinct expression pattern of LTR-Rs and their captured genes in different tissues. (A) The expression of LTR-Rs; (B) the expression of LTR-captured genes. The expression levels of LTR-Rs and genes were normalized to Z-score and the color bars on the right of the heatmaps show the value of Z-score.


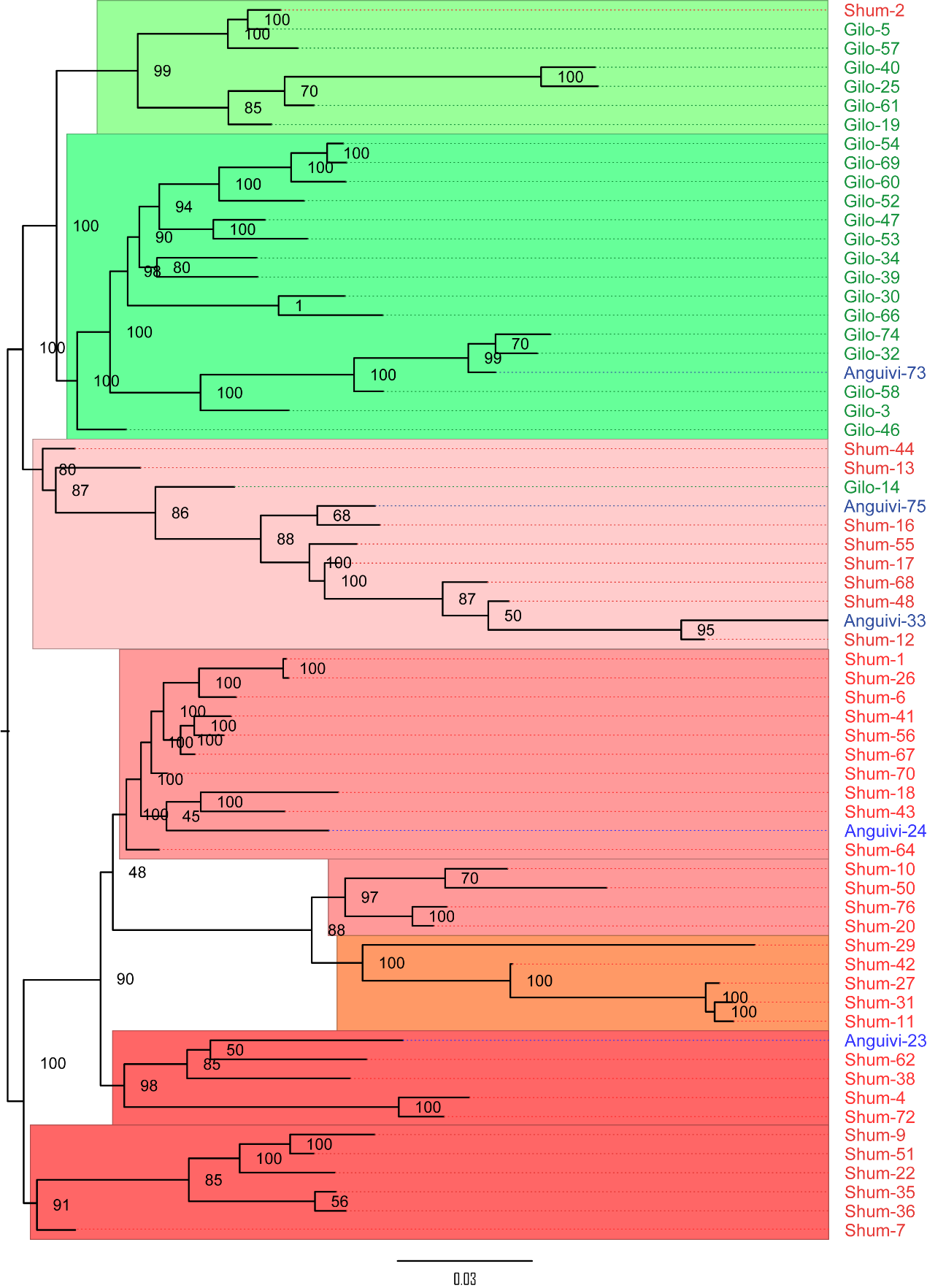


**Supplementary Figure 4.** Maximum-likelihood phylogenetic tree of 65 samples using the full-set of SNPs. The numbers on the branch points of the phylogenetic tree represented bootstrap values of the tree (1,000 replicates); Scale length represented genetic distance.


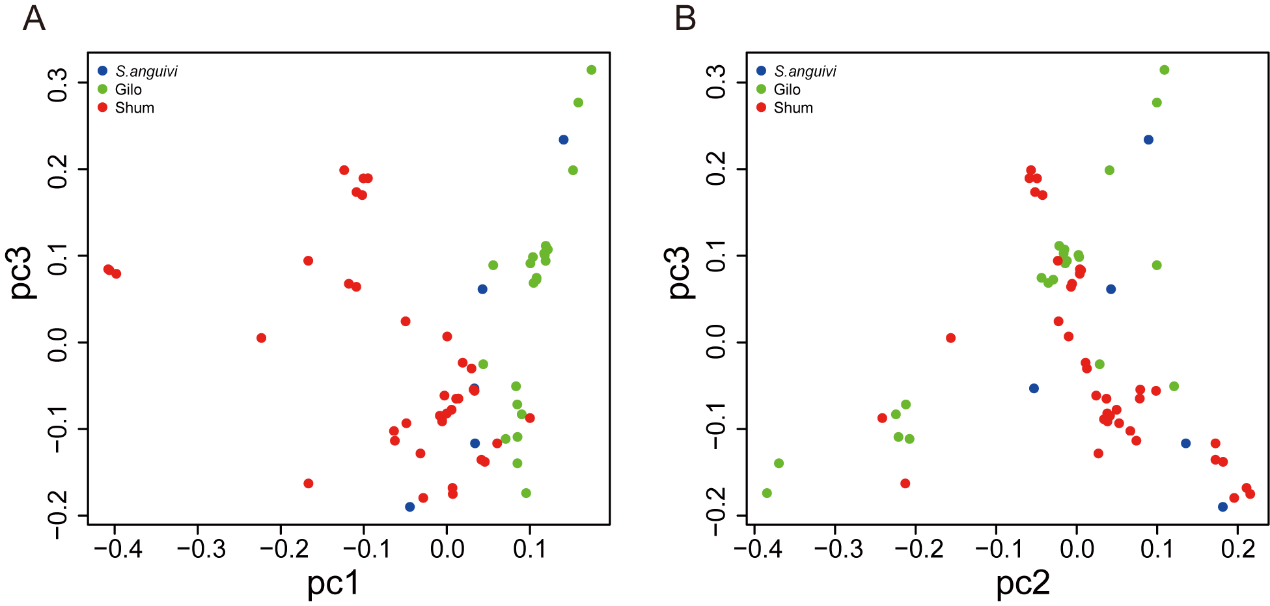


**Supplementary Figure 5.** Principal-component analysis.
