# Supplementary material for: Draft genome sequence of Solanum aethiopicum provides insights into disease resistance, drought tolerance, and the evolution of the genome
Source: Gigascience. 2019 Oct 1;8(10):giz115. doi: 10.1093/gigascience/giz115 (PMC6771550; doi:10.1093/gigascience/giz115)
Supplement: giz115_Response_to_Reviewer_Comments_Original_Submission [file giz115_response_to_reviewer_comments_original_submission.pdf]

Author's Response To Reviewer Comments

Close

Reviewer reports:

Reviewer #1: The manuscript entitled "Draft genome sequence of the Solanum aethiopicum provides insight into disease
study of Solanum aethiopicum, a close relative of the cultivated eggplant Solanum melongena.
Methods are very appropriate to the aims of the study and conclusions are adequately supported by the genomic data.

Could you give more details about the method of:
- The high molecular genomic DNA extraction?
Response: More details and the cited reference were added.
- The selection of high-quality reads?
Response: Details have been added.

- The multiplexing? (barcoding?) and the demultiplexing?
Response: The delivered reads were already demultiplexed.

- The identification of collinearity blocks (parameters of MCscanX)?
Response: Changed to "… gene pairs in MCscanX with default parameters".

- The RNAseq read filtering and removing of low-quality reads (tools, parameters and threshold)?
Response: Details have been added in the text. "SOAPfilter software with the parameters "-M 2, -f 0, -p" was used to filte
>=40% low quality bases or with >=10% uncalled bases ("N") were filtered."

- The variant calling pipeline? (default parameters in GATK for SNP and SV?)
Response: Yes, we used default parameters in GATK pipeline for SNP and SV identified. For quality control, parameters "G
used. Detailed parameters have been added.

- The pan-genome reconstruction (parameters and threshold of SOAPdenovo2 and CD-HIT-EST)?
Response: We use SOAPdenovo2 and CD-HIT-EST software to construct pan-genome with default parameters.

Minor comments:
- Could you describe the eggplant accession used to produce the genome assembly?
Response: A brief description had been added.

- You have used a substitution rate of 1.3e-8 year-1site-1 based on works performed on rice genomes. Could you justify t
Response: Generally, the substitution rate varies little among different plants. For example, the substitution rate reported
generation (Ossowski et al, 2010), which is quite close to that in rice. The use of the rate of rice enables the comparison b
which the same substitution rate was used to infer the ages of LTRs (Kim et al., 2017).

- Could you perform a statistical test to validate the comparison of degeneration of LTR-R activities in different tissues?
Response: Unfortunately, statistical test is not allowed without replicates. Instead, we added regression onto the plots.

- An amplification of LTR is found in Solanum aethiopicum and also in Solanum melongena. Could you give us the referenc
Response: We searched for LTR in S. melongena genome (Hirakawa et al., 2014) in this study. A same method and criteri
comparable.

- The number of SNP seems huge. Could you compare with others plant genomes? (Yuan Fu)
Response: In this study, we had identified 18,614,838 SNPs in total. The number of SNP is highly dependent on the variat
differences of genome sizes also contribute to the varied number of SNP in different species. Actually, it is not fair to comp
populations. Take tomato, whose genome size (828 Mb) is comparable to S. aethiopicum, as an example, a number of 11,
in a population of 360 accessions (Lin et al., 2014). Furthermore, it is not surprise to have such a large number of SNPs in
(Lester et al., 1986).

- "Artificially selected genes", what does the term artificial mean? Could you explain/develop?

Response: It means the genes preferentially retained by human during the history of domestication.

- Numbers of accessory genes seem huge. Could you check if these values are not overestimate due to the presence of fra
Response: The genome sequences per se varies greatly among different groups (Lester et al., 1986), several groups were
cannot completely exclude the possibility of overestimation caused by the presence of fragmented genes, the degree of ov
accessory genes (921 bp) (Supplementary Table 20) is comparable to that of genes (1104 bp) (Supplementary Table 5) in

- "Good quality transcripts" ", what does the term good mean? Could you explain/develop?
Response: It has been rephrased to "The mapped reads were then assembled using StringTie"

- Could you justify the choice of e-value thresholds for gene annotations and gene clustering (1e-4 seems very weak)?
Response: The cutoff of 1e-4 was used for the identification of NLR. It is actually not that weak and had been used in man
Another reason we use this threshold is to make our results comparable to that reported in pepper (Kim et al., 2017), whi

- Could you explain acronyms (GENO, MAF, HWE)?
Response: The full names have been added in the manuscript. They are GENO: Maximum per-SNP missing, MAF: Minor al
value.

Reviewer #2: This paper reports the first genome assembly of Solanum aethiopicum. The description is easy to follow and
eggplant. I recommend the authors to submit the data (genome, genes, protein, annotatoin, sequence variations etc) to S
them easily.
Response: Thanks. That's a very good suggestion. We will arrange the submission upon the acceptance of the paper.

Minor comments:
The term "the reference genome" in the main text should be replaced by "the reference genome sequence".
Response: Replaced. Thanks.

Abstract: LTR-Rs should be spelled out.
Response: Replaced by "long terminal repeat retrotransposons (LTR-Rs)". Thanks. (P2, L12)

Abstract: "closely" is ambiguous.
Response: It is 150 kb. It had been indicated in the text.

Introduction: "We also re-sequenced two ...". Is this 65 (not two) as mentioned in Abstract and other parts?
Response: Changed to "two groups"

Data Description: While a total of 242.6 Gb raw reads were obtained, only 127.83 Gb were used for assembly. I assume t
Response: Yes, the quality of several of the libraries were poor at the beginning of this work, therefore we added more lib

Data Description: Only 80.4% complete BUSCOs were found in the assembly, whereas the total length of the assembly wa
(1.17 Gb). Please clarify the reason for the low BUSCOs. (Yuan Fu, please explain this)
Response: We won't deny that this assembly is only a draft and there must be some genes and sequences missed. In orde
models, we used much more stringent criteria for gene annotation, compared to many other studies on Solanaceae genom
example, the genome of Solanum melongena has as many as 85,446 genes (Hirakawa et al, 2014). In fact, the scores of
criteria for gene annotation. However, this will also include more inaccurate gene models. We had other version of gene s
hoping to removing false annotations as many as possible.

Increased resistance is facilitated by LTR-Rs amplification: What is the definition of "LTR-Rs captured"? It is unclear why t
NLR?
Response: The genes located in LTR-Rs were defined as LTR-Rs captured genes. It is likely that these genes were retropos
are overrepresented by NLRs, we speculate that they are beneficial to disease resistance.

Polymorphisms in different S. aethiopicum groups: What's the difference between indels and SVs?
Response: In this study, we follow the criteria described in the users' guide of GATK pipeline (version 4.0), in which SV is
as short variants including small deletion or insertions.

Artificially selected genes in S. aethiopicum: What types of selections do the authors mention here?
Response: They are the genes preferentially retained by human during the domestication of this crop.

Potential implications: This part can be deleted because this is not based on the data.

Response: removed.

Methods: What are the "standard BGI protocols"?
Response: Changed to "The DNA was sheared into small fragments of ~ 200 bp and used to construct paired-end libraries
al., 2017) and subsequently sequenced on a BGI-500 sequencer. Briefly, the DNA fragments were ligated to BGISEQ-500
amplification, the products of which were then pooled and circularized for sequencing on BGISEQ-500 (BGI, Shenzhen, Ch

SNP calling: "samtools mpileup" and "VariantFiltration" are duplicated.
Response: Corrected.

Reviewer #3: The manuscript describes a draft assembly and annotation for S. aethiopicum genome.
Authors estimated the repetitive elements content and proposed that two amplifications of LTR-Rs occurred around 1.25 a
resistance genes. Authors carried out also comparative genomics study in the Solanaceae family and inferred phylogenetic
aethiopicum and LD.

Although S. aethiopicum is an orphan species and therefore I do not expect the use of the most advanced technologies fo
with HiC, I would have expected at least the anchoring of scaffolds and contigs to pseudomolecules. I think that generatin
obtain, which could be thus genotyped using any GBS approach authors want.
Response: These are very good suggestions. Unfortunately, we do not have extra budget for this at this moment. Of cours
once these data are available.

Although a pan genome of the species was also provided, I think that this paper is not suitable for the publication on this
Furthermore, the language needs tightening up and editing for English sense.
Response: The language has been polished.

More detailed comments
Abstract:
it is reported that the pan-genome of S. aethiopicum contains 1,345 genes are missing in the reference genome. I cannot
Response: The figures in this part have been corrected. Now it has been changed to "A pan-genome of S. aethiopicum wit
of which 24,567 genes are missing in the reference genome sequence." It has also been added in the text.

Background
Line 8-10: I would add some extra reference to this part "It is reported to have medicinal value and its roots and fruits ha
uterine complications in Africa" or clearly highlighted the information got from FAO. Furthermore, FAO should be added to
Response: The publication of these orphan crops is very few, we could only find this information on the website of FAO (h
crops/africangardenegg/en/?amp%3Butm_medium=social%20media&%3Butm_campaign=unfaopinterest), which had alr

Line 24 is (mansfeld.ipk-gatersleben.de). is it a reference for disease resistance? The link send to a database. I would cha
Response: The full address is http://mansfeld.ipk-
gatersleben.de/apex/f?p=185:46:448783208481::NO::module,mf_use,source,akzanz,rehm,akzname,taxid:mf,,botnam,0
which is too long and only the website of home page was shown.
Now, we changed it to "Aculeatum is used as ornamentals (Prohens et al., 2012; Plazas et al., 2014) or rootstocks (mansf
resistance nature (Toppino et al., 2008)"

line 28: please provide at least a reference for this part:"S. aethiopicum is the second most cultivated eggplant, as an "or
Response: This statement has been changed to "Although S. aethiopicum is one of the most important cultivated eggplant
and breeding investments are substantially lagging behind in comparison with other Solanaceae relatives such as tomato,

Line 40 : the sentence on genome editing sound to me a little bit out of place, as no information on genome editing in sca
genome editing might be used for breeding.
Response: We noticed that there is no report of genome editing in S. aethiopicum so far. This is because very few efforts
techniques, just like many other advanced techniques, can eventually be applied into this species to speed the progress o
of genome would be very essential for the identification of genes to be edited, as well as for the design of guide RNAs. Thi
on Physalis pruinose, another orphan crop also in Solanaceae (Lemmon et al., 2018. Nat. Plants), before which there is no

Data description:
I would modify "with a genome size of 1.17 Gb" with "expected genome size". You would get a more precise estimate usin
Response: Changed.

Furthermore, authors generated more than 242Gb of data, but after cleaning, about 50% of the data (128GB) were used [illegible] presumably may explain the number of scaffolds obtained (more than 162k). Did the authors filter for scaffolds' size? Did [illegible] other tools, like SOAP? Any comments?
Response: Yes, the quality of several of the libraries were poor, therefore we added more libraries to make sure the final [illegible] genome using other tools including SOAPdenovo and selected the best assembly for downstream analyses. The assembler [illegible] bp, and all the resulted scaffolds were retained.

Line 33-39. This sentence "Among these annotated TEs, LTR-Rs were extraordinarily abundant and occupied 719 Mbp, [illegible] by LINEs and SINEs (Supplementary Table S4)." is a repetition of what said at the beginning of the paragraph. I will [illegible]
Response: We have deleted this sentence. Thanks.

Line 42 Section protein coding. From table S5 gene features are not so similar to other genomes, especially Pepper and [illegible] genes? The gene number from Kim et al. 2017 is 35,884
Response: Arabidopsis is relatively distant to S. aethiopicum. As for the data of Pepper, the data in this table was [illegible] total of 45,131 protein-coding genes. The data now has been replaced by Kim's data (Kim et al, 2017).

Section Amplification of LTR-Rs:
* please add references here "The proportion of Ty3/Gypsy and Ty1/Copia LTR-Rs in S. aethiopicum is also comparable [illegible]
Response: The references were added. The sentence was rephrased to "The proportion of Ty3/Gypsy in S. aethiopicum is [illegible] genome (87.7% of Ty3/Gypsy in hot pepper)".

* Line 19: In this part "they occurred separately in each genome since S. aethiopicum and hot pepper had split about 20 [illegible]
