# Supplementary material for: Draft genome sequence of Solanum aethiopicum provides insights into disease resistance, drought tolerance, and the evolution of the genome
Source: Gigascience. 2019 Oct 1;8(10):giz115. doi: 10.1093/gigascience/giz115 (PMC6771550; doi:10.1093/gigascience/giz115)
Supplement: giz115_Reviewer_1_Report_Original_Submission — Cyril Jourda -- 3/25/2019 Reviewed [file giz115_reviewer_1_report_original_submission.pdf]

Reviewer Report

Title: Draft genome sequence of the Solanum aethiopicum provides insights into disease resistance, drought tolerance and the evolution of the genome

Version: Original Submission **Date:** 3/25/2019

Reviewer name: Cyril Jourda

Reviewer Comments to Author:

The manuscript entitled "Draft genome sequence of the Solanum aethiopicum provides insight into disease resistance, drought tolerance and evolution" is a genomic study of Solanum aethiopicum, a close relative of the cultivated eggplant Solanum melongena.
Methods are very appropriate to the aims of the study and conclusions are adequately supported by the genomic data.
Could you give more details about the method of:
-     The high molecular genomic DNA extraction?
-     The read filtering (parameters, threshold)?
-     The selection of high-quality reads?
-     The multiplexing? (barcoding?) and the demultiplexing?
-     The identification of collinearity blocks (parameters of MCscanX)?
-     The RNAseq read filtering and removing of low-quality reads (tools, parameters and threshold)?
-     The variant calling pipeline? (default parameters in GATK for SNP and SV?)
-     The pan-genome reconstruction (parameters and threshold of SOAPdenovo2 and CD-HIT-EST)?

Minor comments:
-     Could you describe the eggplant accession used to produce the genome assembly?
-     You have used a substitution rate of 1.3e-8 year-1site-1 based on works performed on rice genomes. Could you justify this?
-     Could you perform a statistical test to validate the comparison of degeneration of LTR-R activities in different tissues?
-     An amplification of LTR is found in Solanum aethiopicum and also in Solanum melongena. Could you give us the reference?
-     The number of SNP seems huge. Could you compare with others plant genomes?
-     "Artificially selected genes", what does the term artificial mean? Could you explain/develop?
-     Numbers of accessory genes seem huge. Could you check if these values are not overestimate due to the presence of fragmented genes?
-     "Good quality transcripts" ", what does the term good mean? Could you

explain/develop?
-     Could you justify the choice of e-value thresholds for gene annotations and gene clustering (1e-4 seems very weak)?
-     Could you explain acronyms (GENO, MAF, HWE)?

Methods

Are the methods appropriate to the aims of the study, are they well described, and are necessary controls included? Choose an item.

Conclusions

Are the conclusions adequately supported by the data shown? Choose an item.

Reporting Standards

Does the manuscript adhere to the journal's guidelines on minimum standards of reporting? Choose an item.

Choose an item.

Statistics

Are you able to assess all statistics in the manuscript, including the appropriateness of statistical tests used? Choose an item.

Quality of Written English

Please indicate the quality of language in the manuscript: Choose an item.

Declaration of Competing Interests

Please complete a declaration of competing interests, considering the following questions:

- Have you in the past five years received reimbursements, fees, funding, or salary from an organisation that may in any way gain or lose financially from the publication of this manuscript, either now or in the future?
- Do you hold any stocks or shares in an organisation that may in any way gain or lose financially from the publication of this manuscript, either now or in the future?
- Do you hold or are you currently applying for any patents relating to the content of the manuscript?
- Have you received reimbursements, fees, funding, or salary from an organization that holds or has applied for patents relating to the content of the manuscript?
- Do you have any other financial competing interests?

- Do you have any non-financial competing interests in relation to this paper?

If you can answer no to all of the above, write 'I declare that I have no competing interests' below. If your reply is yes to any, please give details below.

I declare that I have no competing interests

I agree to the open peer review policy of the journal. I understand that my name will be included on my report to the authors and, if the manuscript is accepted for publication, my named report including any attachments I upload will be posted on the website along with the authors' responses. I agree for my report to be made available under an Open Access Creative Commons CC-BY license (http://creativecommons.org/licenses/by/4.0/). I understand that any comments which I do not wish to be included in my named report can be included as confidential comments to the editors, which will not be published.

Choose an item.

To further support our reviewers, we have joined with Publons, where you can gain additional credit to further highlight your hard work (see: https://publons.com/journal/530/gigascience). On publication of this paper, your review will be automatically added to Publons, you can then choose whether or not to claim your Publons credit. I understand this statement.

Yes Choose an item.
