# Supplementary material for: Draft genome sequence of Solanum aethiopicum provides insights into disease resistance, drought tolerance, and the evolution of the genome
Source: Gigascience. 2019 Oct 1;8(10):giz115. doi: 10.1093/gigascience/giz115 (PMC6771550; doi:10.1093/gigascience/giz115)
Supplement: giz115_Reviewer_2_Report_Original_Submission — Kenta Shirasawa -- 4/3/2019 Reviewed [file giz115_reviewer_2_report_original_submission.pdf]

Reviewer Report

Title: Draft genome sequence of the Solanum aethiopicum provides insights into disease resistance, drought tolerance and the evolution of the genome

Version: Original Submission **Date:** 4/3/2019

Reviewer name: Kenta Shirasawa

Reviewer Comments to Author:

This paper reports the first genome assembly of Solanum aethiopicum. The description is easy to follow and the data would be useful for the breeding programs of eggplant. I recommend the authors to submit the data (genome, genes, protein, annotatoin, sequence variations etc) to Sol Genomics Network so that potential users can access them easily.
Minor comments:
The term "the reference genome" in the main text should be replaced by "the reference genome sequence".
Abstract: LTR-Rs should be spelled out.
Abstract: "closely" is ambiguous.
Introduction: "We also re-sequenced two ...". Is this 65 (not two) as mentioned in Abstract and other parts?
Data Description: While a total of 242.6 Gb raw reads were obtained, only 127.83 Gb were used for assembly. I assume that approximately 115 Gb reads were low quality. Correct?
Data Description: Only 80.4% complete BUSCOs were found in the assembly, whereas the total length of the assembly was 1.02 Gb covering 87% of the estimated genome size (1.17 Gb). Please clarify the reason for the low BUSCOs.
Increased resistance is facilitated by LTR-Rs amplification: What is the definition of "LTR-Rs captured"? It is unclear why the "LTR-Rs captured" genes enhance disease resistance.
NLR?
Polymorphisms in different S. aethiopicum groups: What's the difference between indels and SVs?
Artificially selected genes in S. aethiopicum: What types of selections do the authors mention here?
Potential implications: This part can be deleted because this is not based on the data.
Methods: What are the "standard BGI protocols"?
SNP calling: "samtools mpileup" and "VariantFiltration" are duplicated.

Methods

Are the methods appropriate to the aims of the study, are they well described, and are necessary controls included? Choose an item.

Conclusions

Are the conclusions adequately supported by the data shown? Choose an item.

Reporting Standards

Does the manuscript adhere to the journal's guidelines on minimum standards of reporting? Choose an item.

Choose an item.

Statistics

Are you able to assess all statistics in the manuscript, including the appropriateness of statistical tests used? Choose an item.

Quality of Written English

Please indicate the quality of language in the manuscript: Choose an item.

Declaration of Competing Interests

Please complete a declaration of competing interests, considering the following questions:

- Have you in the past five years received reimbursements, fees, funding, or salary from an organisation that may in any way gain or lose financially from the publication of this manuscript, either now or in the future?
- Do you hold any stocks or shares in an organisation that may in any way gain or lose financially from the publication of this manuscript, either now or in the future?
- Do you hold or are you currently applying for any patents relating to the content of the manuscript?
- Have you received reimbursements, fees, funding, or salary from an organization that holds or has applied for patents relating to the content of the manuscript?
- Do you have any other financial competing interests?
- Do you have any non-financial competing interests in relation to this paper?

If you can answer no to all of the above, write 'I declare that I have no competing interests' below. If your reply is yes to any, please give details below.

I declare that I have no competing interests.

I agree to the open peer review policy of the journal. I understand that my name will be included on my report to the authors and, if the manuscript is accepted for publication, my named report including any attachments I upload will be posted on the website along with the authors' responses. I agree for my report to be made available under an Open Access Creative Commons CC-BY license (http://creativecommons.org/licenses/by/4.0/). I understand that any comments which I do not wish to be included in my named report can be included as confidential comments to the editors, which will not be published.

Choose an item.

To further support our reviewers, we have joined with Publons, where you can gain additional credit to further highlight your hard work (see: https://publons.com/journal/530/gigascience). On publication of this paper, your review will be automatically added to Publons, you can then choose whether or not to claim your Publons credit. I understand this statement.

Yes Choose an item.
