# Supplementary material for: Draft genome sequence of Solanum aethiopicum provides insights into disease resistance, drought tolerance, and the evolution of the genome
Source: Gigascience. 2019 Oct 1;8(10):giz115. doi: 10.1093/gigascience/giz115 (PMC6771550; doi:10.1093/gigascience/giz115)
Supplement: giz115_Reviewer_3_Report_Original_Submission — Lorenzo Barchi -- 4/8/2019 Reviewed [file giz115_reviewer_3_report_original_submission.pdf]

Reviewer Report

Title: Draft genome sequence of the Solanum aethiopicum provides insights into disease resistance, drought tolerance and the evolution of the genome

Version: Original Submission **Date:** 4/8/2019

Reviewer name: Lorenzo Barchi

Reviewer Comments to Author:

The manuscript describes a draft assembly and annotation for S. aethiopicum genome.
Authors estimated the repetitive elements content and proposed that two amplifications of LTR-Rs occurred around 1.25 and 3.5 million years ago, resulting in the expansion of resistance genes. Authors carried out also comparative genomics study in the Solanaceae family and inferred phylogenetic studies as well as the domestication history of S. aethiopicum and LD.
Although S. aethiopicum is an orphan species and therefore I do not expect the use of the most advanced technologies for assembly such as PacBio and chromosome scaffolding with HiC, I would have expected at least the anchoring of scaffolds and contigs to pseudomolecules. I think that generating an F2 mapping population for S. aethiopicum is easy to obtain, which could be thus genotyped using any GBS approach authors want.
Although a pan genome of the species was also provided, I think that this paper is not suitable for the publication on this journal.
Furthermore, the language needs tightening up and editing for English sense.
More detailed comments
Abstract:
it is reported that the pan-genome of S. aethiopicum contains 1,345 genes are missing in the reference genome. I cannot find this in the main text.
Background
Line 8-10: I would add some extra reference to this part "It is reported to have medicinal value and its roots and fruits have been used to treat colic, high blood pressure and uterine complications in Africa" or clearly highlighted the information got from FAO. Furthermore, FAO should be added to reference list
Line 24 is (mansfeld.ipk-gatersleben.de). is it a reference for disease resistance? The link send to a database. I would change it with some references from literature.
line 28: please provide at least a reference for this part:"S. aethiopicum is the second most cultivated eggplant, as an "orphan crop"
Line 40 : the sentence on genome editing sound to me a little bit out of place, as no information on genome editing in scarlet aethiopicum is available. I would point out that genome editing might be used for breeding.
Data description:
I would modify "with a genome size of 1.17 Gb" with "expected genome size". You would get a more precise estimate using flow-cytometry.
Furthermore, authors generated more than 242Gb of data, but after cleaning, about 50% of the data

(128GB) were used for assembly, which is a quite high percentage. This presumably may explain the number of scaffolds obtained (more than 162k). Did the authors filter for scaffolds' size? Did the authors try to assembly the genome sequence with other tools, like SOAP? Any comments?
Line 33-39. This sentence "Among these annotated TEs, LTR-Rs were extraordinarily abundant and occupied 719 Mbp, accounting for approximately 70% of the genome, followed by LINEs and SINEs (Supplementary Table S4)." is a repetition of what said at the beginning of the paragraph. I will combine the two sentences.
Line 42 Section protein coding. From table S5 gene features are not so similar to other genomes, especially Pepper and Arabidopsis. Furthermore, why pepper has more than 45k genes? The gene number from Kim et al. 2017 is 35,884
Section Amplification of LTR-Rs:
*   please add references here "The proportion of Ty3/Gypsy and Ty1/Copia LTR-Rs in S. aethiopicum is also comparable to those reported in other Solanaceae genomes."
*   Line 19: In this part "they occurred separately in each genome since S. aethiopicum and hot pepper had split about 20 MYA (Figure 1A), and about 4 MYA between S. aethiopicum and tomato (Figure 1A)." authors stated that S. aethiopicum separated from tomato 4 million years ago. This sound strange. S.aethiopicum did not separated from tomato 4 MYA, but only the ancestors of tomato/potato and eggplant/scarlet eggplant, which occurred around 16MYA. Furthermore, the second LTR burst occurred 1.25MYA was also shared by eggplant?
Polymorphisms in different S. aethiopicum groups section:
Concerning the ADMIXTURE analysis and results, I wonder why authors did not define accessions belonging for less than, let's say 70%, to a group as admixed.
Artificially selected genes in S. aethiopicum
I would have expected, at least for the 12 genes in common between Gilo and Shum (and maybe for the 36 selected genes in Shum), some more information. What genes are they?
Go enrichments are nice but sometimes it would be better to provide some more details, especially if the number of genes involved are limited.
Pan-genome section
*   Why did the authors get less contigs for Anguivi? The sequencing performance are quite good for the 5 accessions of this species.
*   I am quite confused on the metrics (Supplementary table S20). In the text, it is reported that 41,626, 22,942 and 17,726 protein-coding genes for "Shum", "Gilo" and "S. anguivi, respectively were predicted, among which accessory gene sets of 29,389, 23,726 and 12,829 for "Shum", "Gilo" and "S. anguivi", respectively were found.
These numbers are not the same in S20 table, presumably two columns were switched.
Furthermore in the table S22 for Gilo, a total of 33,194 gene are reported, while in the text the number is 22,942. Accessory genes in the text for Gilo are less than the ones predicted (as reported in the text).
*   Table S20, I will add the unit of measurement for length
*   I cannot find Supplementary Table S21 and S22
Methods
Gene family analysis: References for the 5 proteomes used are missing, as well as the version used
NLR genes: it is not clear to me how the NLR genes were identified. In methods is reported that specific

NB-ARC HMM model was constructed, but in the text it is reported that NBS-LRR genes were identified. How did the authors performed the identification of other Motifs (TIR, CC and LRR)?
SNP calling: which parameters did the authors use for SNP identification? Besides MAF and GENO parameters, I would also have considered sequencing depth as a key parameter for the final SNPs set.
Population analyses. I would add bootstrap values to the figure 5A
Furthermore, is the reference for Itools (80) correct?

Methods

Are the methods appropriate to the aims of the study, are they well described, and are necessary controls included? Choose an item.

Conclusions

Are the conclusions adequately supported by the data shown? Choose an item.

Reporting Standards

Does the manuscript adhere to the journal's guidelines on minimum standards of reporting? Choose an item.

Choose an item.

Statistics

Are you able to assess all statistics in the manuscript, including the appropriateness of statistical tests used? Choose an item.

Quality of Written English

Please indicate the quality of language in the manuscript: Choose an item.

Declaration of Competing Interests

Please complete a declaration of competing interests, considering the following questions:

- Have you in the past five years received reimbursements, fees, funding, or salary from an organisation that may in any way gain or lose financially from the publication of this manuscript, either now or in the future?
- Do you hold any stocks or shares in an organisation that may in any way gain or lose financially from the publication of this manuscript, either now or in the future?
- Do you hold or are you currently applying for any patents relating to the content of the manuscript?

- Have you received reimbursements, fees, funding, or salary from an organization that holds or has applied for patents relating to the content of the manuscript?
- Do you have any other financial competing interests?
- Do you have any non-financial competing interests in relation to this paper?

If you can answer no to all of the above, write 'I declare that I have no competing interests' below. If your reply is yes to any, please give details below.

I declare that I have no competing interests

I agree to the open peer review policy of the journal. I understand that my name will be included on my report to the authors and, if the manuscript is accepted for publication, my named report including any attachments I upload will be posted on the website along with the authors' responses. I agree for my report to be made available under an Open Access Creative Commons CC-BY license (http://creativecommons.org/licenses/by/4.0/). I understand that any comments which I do not wish to be included in my named report can be included as confidential comments to the editors, which will not be published.

Choose an item.

To further support our reviewers, we have joined with Publons, where you can gain additional credit to further highlight your hard work (see: https://publons.com/journal/530/gigascience). On publication of this paper, your review will be automatically added to Publons, you can then choose whether or not to claim your Publons credit. I understand this statement.

Yes Choose an item.
